# Cardiac transcriptional and metabolic changes following thoracotomy

**DOI:** 10.1038/s41598-020-66721-3

**Published:** 2020-06-15

**Authors:** Markus B. Heckmann, Ashraf Yusuf Rangrez, Daniel Finke, Andreas Jungmann, Julia S. Kreußer, Alexandra Rosskopf, Nesrin Schmiedel, Hugo A. Katus, Norbert Frey, Oliver J. Müller

**Affiliations:** 1 Department of Internal Medicine III, Cardiology, Angiology & Pulmonology, Heidelberg University Hospital, Im Neuenheimer Feld 669, 69120 Heidelberg, Germany; 2 DZHK (German Center for Cardiovascular Research), partner site Heidelberg/Mannheim, Heidelberg, Germany; 3 Department of Internal Medicine III, University of Kiel, Arnold-Heller-Str. 3, 24105 Kiel, Germany; 4 DZHK (German Centre for Cardiovascular Research), Partner Site Hamburg/Kiel/Lübeck, Kiel, Germany

**Keywords:** Cardiovascular biology, Experimental models of disease, Cardiology, Molecular medicine

## Abstract

Non-cardiac surgery is associated with significant cardiovascular complications. Reported mortality rate ranges from 1.9% to 4% in unselected patients. A postoperative surge in pro-inflammatory cytokines is a well-known feature and putative contributor to these complications. Despite much clinical research, little is known about the biomolecular changes in cardiac tissue following non-cardiac surgery. In order to increase our understanding, we analyzed whole-transcriptional and metabolic profiling data sets from hearts of mice harvested two, four, and six weeks following isolated thoracotomy. Hearts from healthy litter-mates served as controls. Functional network enrichment analyses showed a distinct impact on cardiac transcription two weeks after surgery characterized by a downregulation of mitochondrial pathways in the absence of significant metabolic alterations. Transcriptional changes were not detectable four and six weeks following surgery. Our study shows distinct and reversible transcriptional changes within the first two weeks following isolated thoracotomy. This coincides with a time period, in which most cardiovascular events happen.

## Introduction

Non-cardiac surgery is associated with significant. Reported mortality rate ranges from 1.9% to 4% in unselected patients^1,2^. With 45% of reported cases, cardiovascular death is a major contributor^2^, with most cardiovascular events occurring within the first two weeks after surgery^3^.

Following surgery, increased levels of IL-1, IL-6 and TNF-alpha have been reported, and increased C-reactive protein levels and elevated white blood cell counts indicative of a systemic inflammatory response are frequently observed^4^.

These findings prompted clinical trials investigating the effect of perioperative statin and betablocker-therapy in non-cardiac surgery^3,5^. Despite these high impact clinical trials, little is known about the biomolecular changes in cardiac tissue following non-cardiac surgery. Aim of this study was to improve our understanding of the effect of isolated thoracotomy (ITH) on the cardiac metabolome and transcriptome over time.

## Methods

### Animal handling

As previously described^6^, eight-week-old male mice (C57BL/6 N—Charles River, Sulzfeld, Germany) were randomly subjected to isolated thoracotomy (ITH) or transaortic banding (TAC)^7^. Healthy littermates were used as controls. Mice were sacrificed after 2 weeks (TAC n = 10, ITH n = 7, controls n = 5), 4 weeks (TAC n = 18, ITH n = 8, controls n = 5), and 6 weeks (TAC n = 11, ITH n = 10, controls n = 5). One TAC animal of the 6 weeks group survived for 10 weeks and was included in the TAC analysis as previously reported^6^. All mice were assessed by echocardiography as previously described^8^. A confirmatory cohort was also subjected to TAC or ITH and sacrificed 2 weeks after surgery (TAC n = 7, ITH n = 7, controls n = 7). In this cohort, controls were also subjected to sedoanalgesia. Animals were fed ad libitum with Rod 16-A (LASvendi, Soest, Germany) and housed in a specific pathogen free environment as previously described. All procedures involving the use and care of animals were performed according to the Directive 2010/63/EU of the European Parliament and the German animal protection code. Permission was granted by local authorities (Regierungspräsidium Karlsruhe, Germany, (G122/12 and A16/09) and Ministerium für Energiewende, Landwirtschaft, Umwelt, Natur und Digitalisierung (MELUND) Kiel, Germany (129-10/17)).

### Microarray analysis and metabolite profiling

RNA was purified from total heart tissue and cDNA expression data were generated in the microarray unit of the German Cancer Research Center (DKFZ, Heidelberg) using the Illumina TotalPrep RNA Amplification kit (Ambion) and Illumina’s MouseWG-6 v1.1 array as previously described^6^. Unbiased metabolite profiling comprising 450 different metabolites was performed as previously described^6^.

### Quantitative reverse transcriptase PCR

RNA was isolated from samples using the RNeasyFibrous Tissue Mini Kit (Qiagen), 0.6 μg RNA was transcribed into cDNA with the help of the Superscript III Kit (Invitrogen) and RNA digestion was performed using RNase H. The following primers were used: ART3 - For AAATGGTCACCACGCTGCT Rev CTCCTCCCTCTTCATCTGCG; COX7B - For ACCAGAAGAGGGCACCTAGT Rev TTCCTTTGGGGTGACTCTGC; FH1 - For GACAACTGTGTGGTCGGGAT Rev CGTTCTTGTGTGCGGTCTTG; LMO7 - For GAGGCTCAGAGATGGGTGGA Rev TCTTCTTAACGACGCCAGGTT; NDUFA5 - For CGGGCTTGCTGAAAAAGACAA Rev TCCCATGGCTTCCACTTCAA; NDUFS4 - For GGCGGTCTCAATGTCAGTGT Rev TGTCCCGAGTCTGGTTGTCT; NR1D2 - For CAACGGCAATCCCAAGAACG Rev AATCCTGATGCCACATCCCC; PAIP2 - For AGCAGTACTAGCCCAAGCATC Rev CCAGCATTTCTTGGAAACAGC; PDHB - For AAAGGCAAGGGACCCACATC Rev CCTCCTTCCACAGTCACGAG; PMPCB - For TTACACGAAGGCTTCCGCTT Rev CACGTTGAGAGCCCAGAGTT; SDHD - For GTGACCTTGAGCCCTCGAAA Rev GCTGGTCCTGGAGAAATGCT; TJP1—For CGGCCGCTAAGAGCACAG, Rev TGGAGGTTTCCCCACTCTGA; YWHAE—For ACCGGCAAATGGTTGAAACTG, Rev TGTGGCAAACTCAGCCAGAT; and RPL32 (as an endogenous control) - For GGTGGCTGCCATCTGTTTTACG Rev CCGCACCCTGTTGTCAATGC. cDNA was quantified using iTaq Universal SYBR Green Supermix (BioRad) on a CFX96 Touch Real-Time PCR detection system (BioRad) applying a common two-step protocol (60 °C; 95 °C; 25 cycles). Two technical replicates were run for each reaction. ddCt method for qrtPCR data analysis was used.

### Protein preparation and western blotting

Total protein from mouse heart was extracted in RIPA buffer (20 mM Tris, 10 mM DTT, 500 mM Sodium chloride, 1% NP40, 12,5% Glycerol) supplemented with phosphatase and protease inhibitor cocktails (Roche, Germany). Cell debris was removed by centrifuging the protein lysate at 12,000 × g for 20 min. and supernatant was used for protein concentration measurement by DC-assay (Bio-Rad Laboratories). Proteins (25 µg) were resolved on commercially available 4–12% gradient gels (Life Technologies), and transferred to PVDF (polyvinylidenefluoride) membrane. Membranes were blocked for 2 h in 5% (YAP) or 1% (OXPHOS) dry-milk prepared in 0.1% TBST at room temperature (RT), followed by the incubation with respective primary antibodies (anti YAP (1:1000, polyclonal, rabbit, Cell Signaling); anti OXPHOS (1:500, monoclonal, mouse, Abcam); anti-GAPDH (1:2000, polyclonal, donkey, Santa Cruz); anti p-YAP1-Ser127 (1:1000, monoclonal, rabbit, Cell Signaling); anti p-YAP1-Ser397 (1:1000, monoclonal, rabbit, Cell Signaling); anti MST1 (1:1000, polyclonal, rabbit, Cell Signaling); anti MST1-Thr183 (1:1000, monoclonal, rabbit, Cell Signaling); anti LATS1 (1:1000, monoclonal, rabbit, Cell Signaling); anti p-LATS-Thr1079 (1:1000 monoclonal, rabbit, Cell Signaling); anti MOB1 (1:1000, monoclonal, rabbit, Cell Signaling); anti p-MOB1-Thr35 (1:1000, monoclonal, rabbit, Cell Signaling) and anti α-Tubulin (1:5000, monoclonal, mouse, Sigma)) overnight at 4 °C. Membranes were then washed 4x times with 0.1% TBST and incubated with a suitable HRP-coupled secondary antibody (1:10000) (Santa Cruz, Germany) for 1 h. Protein signals were visualized using ECL-select chemiluminescence kit (GE Healthcare) on Fluorchem Q imaging system (Biozym). Quantitative densitometry was performed using ImageJ/Fiji version 1.46.

### Statistics and bioinformatics

Ratios of medians were calculated for metabolite and gene expression values. An ANOVA was conducted using the statistical software R Version 3.4.4^9^. P-values were calculated and false discovery rates (FDR/q-values) were determined using the Benjamini Hochberg procedure. An FDR < 0.05 and a p-value <0.01 was considered significant. Clustered heat maps were created using the gplots package in R^10^. Data was reported as mean ± standard deviation. Normally distributed data was compared using a student’s t test.

KEGG, Reactome, and gene ontology (GO) pathway enrichment was performed for the set of significant genes using the DOSE, ReactomePA, and clusterProfiler packages^11–13^.

Promoter motif enrichment was performed with Homer analyzing the sequences 1000 bp upstream the transcription start site of all significant genes^14^. De novo motifs which were not possibly false positive and known motifs with a false discovery rate below 0.05 were considered significant. The default mouse promoter set was used as background. Interactions were annotated using the mm9 mouse genome. Interaction networks were displayed using Cytoscape 3.4^15^.

Transcription factor pathways were investigated using Reactome and KEGG pathways. MicroRNA interactions were analyzed with the multiMir package combining data from DIANA-microT, ElMMo, MicroCosm/miRBase, miRanda, miRDB, PicTar, PITA, TargetScan, miRecords, miRTarBase and TarBase^16^. Only strong validated interactions were considered relevant. For predicted interactions, the top 20% of all microRNAs binding to conserved sites of the target were considered in each database. Predicted interactions were considered relevant when confirmed by three or more databases.

Expression patterns were compared by creating similarity ratio, namely the number of the number of genes, which were significantly up- or downregulated in the same direction or which were not significant in both datasets, divided by the number of genes of interest. P-values were calculated by comparing the calculated ratios to ratios established by comparing randomly selected genes of the same dataset to the reference dataset. The p-value was the number of random patterns, which reached higher similarity ratios than the pattern analyzed, divided by the number of permutations. 100.000 permutations were performed. A p-value below 0.05 was considered significant.

qrtPCT and western blot data was analyzed by one-way ANOVA using SigmaPlot version 13.0. A two-sided Student’s t test was performed, when only two groups were compared.

## Results

Transcriptional and metabolic changes following TAC surgery compared to isolated thoracotomy have been described previously^6^. As expected, isolated thoracotomy did not change left ventricular function compared to controls. Left ventricular fractional shortening was 72 ± 6% in ITH animals and 75 ± 3% in controls 2 weeks (p = 0.21), 70 ± 8% in ITH mice 74 ± 1% in controls 4 weeks (p = 0.22), and 65 ± 7% in ITH animals 64 ± 6% in controls (p = 0.55) 6 weeks after surgery. We did not observe any major cardiovascular or cerebral events in our animals following surgery (0.0% [0%;16%], 95% confidence interval (Wilson), n = 25). The event rate is comparable to clinical data published reporting an event rate of 6.5% following thoracic non-cardiac surgery in humans (p = 0.36, Yate’s Χ^2^ test)^17^.

**Figure 1:**
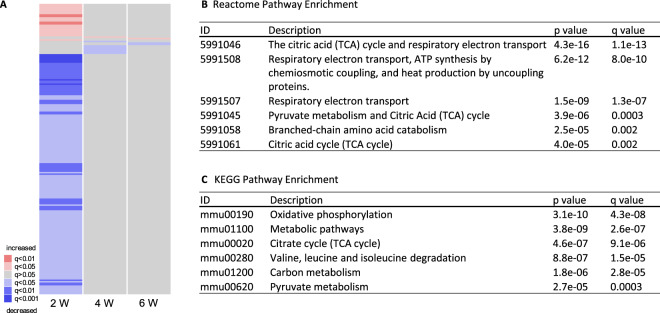
Transcriptional patterns and gene enrichment analysis in cardiac tissue. (**a**) Heatmap of all significant genes 2 weeks (W), 4 weeks, and 6 weeks following isolated thoracotomy (ITH). Note the distinct pattern two weeks after surgery, which is not present any more 4 and 6 weeks after surgery. (**b**) Reactome and (**c**) KEGG pathway enrichment analysis two weeks after surgery shows significant downregulation of mitochondrial pathways involved in energy production and branched-chain amino acid metabolism. GO pathways enrichment analysis is reported in Table 1. Animal numbers at 2/4/6 weeks: ITH n = 7/8/10, control=5/5/5. Heatmaps were created using R and gplots^9,10^. Gene enrichment analysis was performed using ReactomePA and clusterProfiler^12,13^.

Transcriptional profiling of cardiac tissue revealed 186 significantly altered mRNAs 2 weeks after surgery, while only 8 mRNAs were altered 4 weeks, and only 3 mRNAs 6 weeks after surgery (see Fig. 1a). Transcriptional data can be accessed at ArrayExpress: Acc.# E-MTAB-8910. KEGG, Reactome and GO enrichment 2 weeks after surgery showed significant downregulation of primarily mitochondrial pathways involving energy production (see Fig. 1b,c and Table 1). Oxidative phosphorylation and citrate cycle were significantly downregulated. Furthermore, metabolic pathways including branched-chain amino acid degradation were significantly altered.

**Table 1:** GO enrichment analysis 2 weeks after ITH.

ID	Description	P value	Q value
GO:0045333	cellular respiration	6.6e-12	1.1e-08
GO:0051186	cofactor metabolic process	1.3e-10	9.4e-08
GO:0006091	generation of precursor metabolites and energy	1.8e-10	9.4e-08
GO:0015980	energy derivation by oxidation of organic compounds	2.7e-10	1.1e-07
GO:0022900	electron transport chain	4.8e-10	1.5e-07
GO:0006732	coenzyme metabolic process	5.1e-09	1.4e-06
GO:0046128	purine ribonucleoside metabolic process	2.5e-08	5.0e-06
GO:1901657	glycosyl compound metabolic process	2.5e-08	5.0e-06
GO:0042278	purine nucleoside metabolic process	3.0e-08	5.3e-06
GO:0006733	oxidoreduction coenzyme metabolic process	4.6e-08	7.3e-06
GO:0009119	ribonucleoside metabolic process	5.5e-08	7.9e-06
GO:0009116	nucleoside metabolic process	9.6e-08	1.3e-05
GO:0022904	respiratory electron transport chain	1.3e-07	1.6e-05
GO:0051188	cofactor biosynthetic process	2.3e-07	2.6e-05
GO:0046034	ATP metabolic process	6.1e-07	6.5e-05
GO:0006099	tricarboxylic acid cycle	1.1e-06	0.0001
GO:0009205	purine ribonucleoside triphosphate metabolic process	1.4e-06	0.0001
GO:0009199	ribonucleoside triphosphate metabolic process	1.6e-06	0.0001
GO:0009167	purine ribonucleoside monophosphate metabolic process	1.9e-06	0.0001
GO:0009126	purine nucleoside monophosphate metabolic process	2.0e-06	0.0001
GO:0009144	purine nucleoside triphosphate metabolic process	2.0e-06	0.0001
GO:0009161	ribonucleoside monophosphate metabolic process	2.1e-06	0.0002
GO:0009108	coenzyme biosynthetic process	2.4e-06	0.0002
GO:0046496	nicotinamide nucleotide metabolic process	2.5e-06	0.0002
GO:0019362	pyridine nucleotide metabolic process	2.8e-06	0.0002
GO:0009123	nucleoside monophosphate metabolic process	2.8e-06	0.0002
GO:0006979	response to oxidative stress	2.8e-06	0.0002
GO:0009060	aerobic respiration	3.0e-06	0.0002
GO:0072524	pyridine-containing compound metabolic process	3.1e-06	0.0002
GO:0009141	nucleoside triphosphate metabolic process	4.0e-06	0.0002
GO:0042773	ATP synthesis coupled electron transport	9.7e-06	0.0005
GO:0072521	purine-containing compound metabolic process	1.6e-05	0.0008
GO:0006086	acetyl-CoA biosynthetic process from pyruvate	6.1e-05	0.003
GO:0006119	oxidative phosphorylation	6.8e-05	0.003
GO:0006635	fatty acid beta-oxidation	0.0001	0.005
GO:0042775	mitochondrial ATP synthesis coupled electron transport	0.0001	0.006
GO:0006085	acetyl-CoA biosynthetic process	0.0002	0.006
GO:0043648	dicarboxylic acid metabolic process	0.0002	0.006

In congruence with the fact that most genes were downregulated, upregulated genes included genes mostly involved in transcription inhibition (PCGF1, ZNFX1, PUF60), splicing modulation (RBM42, PUF60), inhibition of autophagy (TMEM208, ATP13A2) and protein synthesis and degradation (DNAJB10, B3GALT1, COG1, MTIF3). A complete list of all significantly deregulated genes can be found in supplementary Table 1.

**Figure 2:**
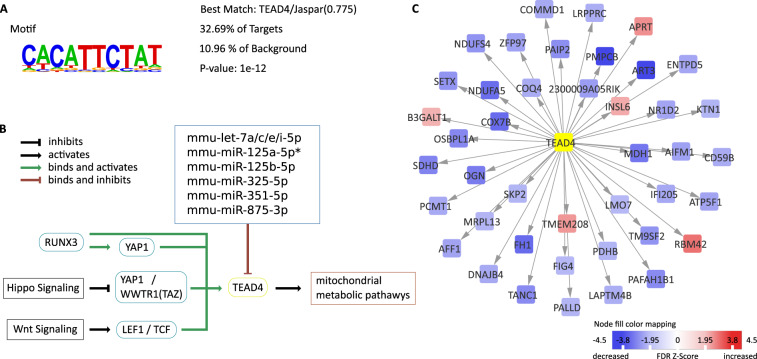
TEAD4, its targets and regulatory pathways. (**a**) promoter enrichment analysis showed an enrichment in the CACATTCTAT motif—a binding motif of TEAD4. (**b**) displays TEAD4 signaling and significant predicted (*and validated) microRNA binding. As most target genes of TEAD4 are downregulated in our dataset (**c**), altered upstream signaling, miR binding or inhibition of TEAD4 might have led to downregulation of mitochondrial pathways in the heart. Animal numbers at 2 weeks: ITH n = 7, control=5.

We further performed a de novo motif enrichment analysis on the promoter regions of the involved genes, which showed a significant enrichment of the CACATTCTAT-Motif, a binding site for TEAD4 (see Fig. 2a). According to a pathway analysis using interactions annotated in Reactome and KEGG, TEAD4 is activated by the RUNX3, Hippo and Wnt pathways by RUNX3, YAP1, TCF, and LEF1 (see Fig. 2b). It plays a crucial role in energy hemostasis during embryogenesis^18^. In our dataset, the target genes of TEAD4 were significantly downregulated compared to background (p < 2 × 10^−5^, Fisher’s exact test). The vast majority of significantly altered genes 2 weeks after ITH controlled by TEAD4 were downregulated (see Fig. 2c). As this might be due to microRNA binding as previously described for TEAD4 and miR-125a-5p, we searched for known and predicted miR interactions^19^. Searching multiple databases as described above, we identified let-7a/c/e/i-5p, miR-125a-5p, miR-125b-5p, miR-325-5p, miR-351-5p, and miR-875-3p as the most probable microRNAs binding to TEAD4.

**Figure 3:**
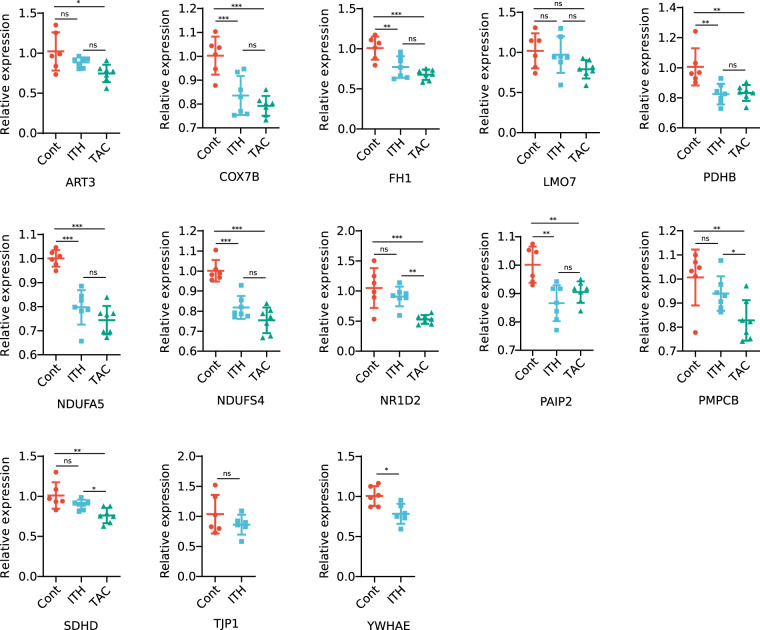
Cardiac gene expression measured by qPCR in a second cohort. Transcriptional changes of significantly deregulated genes targeted by TEAD4 measured in a second cohort 2 weeks after surgery. Expression levels are displayed relative to the mean expression of the control group. Mean and standard deviation are depicted. Key mitochondrial genes involved in the respiratory chain are downregulated 2 weeks after surgery. Animal numbers: control n = 6, ITH n = 7, TAC n = 7. See Table 2 for a direct comparison of microarray and qPCR data between the two cohorts.

In order to validate our microarray results in an independent cohort, we analyzed the expression of some of the significantly deregulated genes harboring the investigated TEAD motif in their promoter regions by qrtPCR. ART3, COX7B, FH1, LMO7, NDUFA5, NDUFS4, NR1D2, PAIP2, PDHB, PMPCB and SDHD constituted the most regulated genes in our microarray analysis with ratios ranging from 0.58 and 0.69. Most of these genes also showed a decreased expression following surgery in our second cohort (see Table 2). COX7B, FH1, NDUFA5, NDUSF5, PAIP2 and PDHB were also significantly downregulated not only in TAC but also following ITH (Fig. 3). Comparatively little is known about the exact function of the repressor PAIP2 in cardiomyocytes, while COX7B, FH1, NDUFA5, NDUSF5 and PDHB are all well-known essential parts of the mitochondrial respiratory chain.

**Table 2:** Comparison of microarray findings in cohort one with qPCR results in cohort 2.

gene symbol	microarray ratio	microarray FDR	qPCR ratio	qPCR P value
ART3	0.58	<0.001	0.87	0.11
COX7B	0.66	0.002	0.83	<0.001
FH1	0.63	0.002	0.77	0.002
LMO7	0.63	0.04	0.95	0.71
NDUFA5	0.58	0.001	0.80	<0.001
NDUFS4	0.65	0.01	0.82	<0.001
NR1D2	0.69	0.02	0.87	0.34
PAIP2	0.70	0.008	0.86	0.007
PDHB	0.64	0.04	0.82	0.002
PMPCB	0.63	<0.001	0.93	0.23
SDHD	0.64	0.005	0.89	0.12
TJP1	0.79	0.03	0.86	0.26
YWHAE	0.89	0.32	0.78	0.01

FDR: false discovery rate.

**Figure 4:**
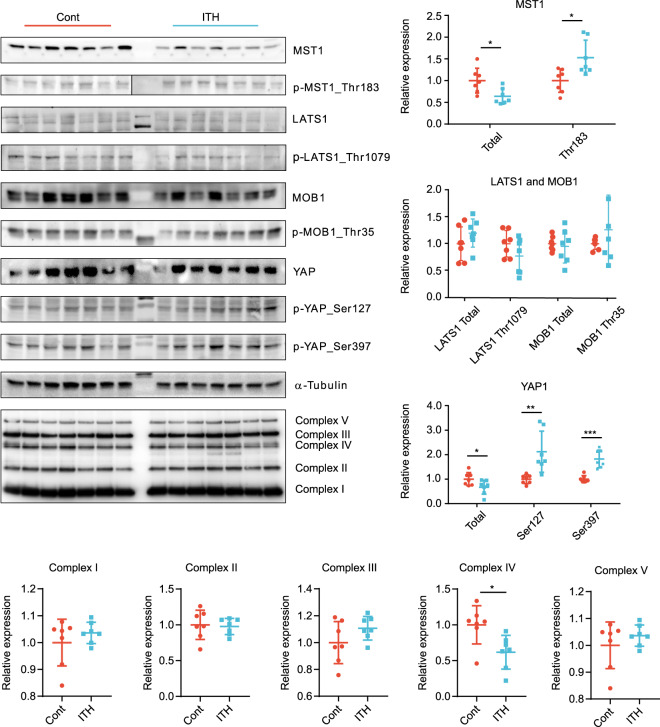
MST1 and YAP1 protein expression and phosphorylation as well as oxidative phosphorylation following isolated thoracotomy. MST1 protein expression was notably reduced, while phosphorylation was significantly increased. YAP1 protein expression was also decreased and Ser127 and Ser397 phosphorylation (LATS1 phosphorylation sites) was significantly increased following isolated thoracotomy. Complexes I-III and V do not show any significant changes after thoracotomy, while decreased levels of complex IV were noted two weeks after ITH. For uncropped immunoblots see supplementary Fig. 1. ITH n = 7, controls n = 7. *p < 0.05, **p < 0.01, ***p < 0.001.

To further investigate potential upstream alterations that might be involved in creating the transcriptomic signature, we measured MST1 and YAP1 protein expression and phosphorylation (see Fig. 4). MST1 was moderately decreased two weeks after ITH. We also found an increase in MST1 phosphorylation which is considered a key initiating event in Hippo signaling. Consistent with activated Hippo signaling, YAP protein levels were reduced while YAP1 phosphorylation was significantly increased on both LATS1 phosophorylation sites (Ser 127 and Ser 397) although LATS1 and MOB1 phosphorylation was not significantly altered following surgery. YAP phosphorylation likely contributed to enhanced YAP1 degradation. As TJP1 has been closely related to Hippo signaling activity and 14-3-3 (gene name: YWHAE) is known to protect cytosolic YAP1 from degradation, we measured both TJP1 and YWHAE gene expression and found decreased levels two weeks following ITH (see Fig. 3 and Table 2). Taken together, these findings suggest an increase in Hippo signaling leading to increased YAP1 degradation and decreased TJP1 expression.

Interestingly, these significant changes in core metabolic pathways did not lead to changes in the metabolome. We analyzed a panel of 450 metabolites in the heart and the liver 2 weeks after ITH (see supplementary tables 2–5). Even when lowering the threshold of the false discovery rate to 0.2, no metabolite measured was depleted or accumulated in the heart or the liver 2 weeks after ITH. Although this does not rule out a change in metabolic flux, the transcriptome is apparently much more prone to significant alterations after subtle stress than the metabolome.

**Figure 5:**
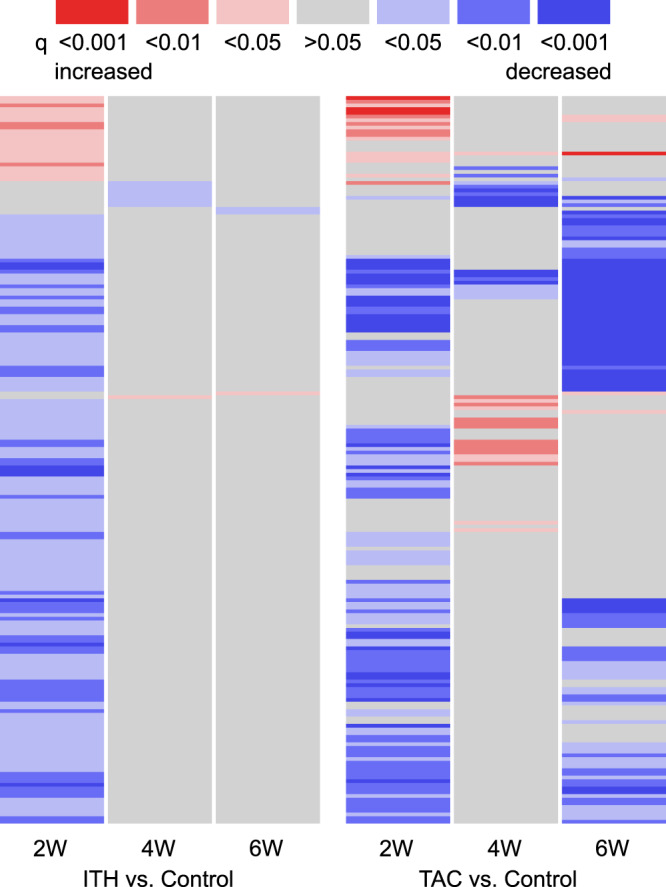
Transcriptional changes after ITH and TAC. Transcriptional changes noted after ITH are also seen 2 weeks (W) after TAC. The pattern disappears four weeks after TAC and reappears with decompensated heart failure 6 weeks after TAC. Interestingly, transcriptional changes do not lead to metabolite accumulation or depletion 2 weeks after ITH, while significant metabolic perturbations are noted following TAC. Animal numbers at 2/4/6 weeks: ITH n = 7/8/10, control=5/5/5, TAC n = 10/18/11. Heatmaps were created using R and gplots^9,10^.

As previously published, metabolic changes following TAC are significantly more pronounced even as early as two weeks after surgery (0/450 ITH vs. 110/450 TAC, p < 1 × 10^−16^, Fisher’s exact test)^6^. Considering all genes significantly altered two weeks after ITH, TAC animals show similar alterations two weeks and six weeks after TAC (see Fig. 5). 72.58% of the genes significantly expressed two weeks after ITH were expressed in the same manner two weeks after TAC (p < 0.00001, permutation test). Interestingly, this pattern disappeared four weeks after TAC (similarity: 4.84% similarity [ITH 2 weeks vs. TAC 4weeks], p = 0.36, permutation test), and re-appeared after six weeks (similarity: 51.61% [ITH 2 weeks vs. TAC 6weeks], p < 0.00001, permutation test).

## Discussion

Cardiovascular events are a major complication following non-cardiac surgery^2^. Vast clinical trials have evaluated different perioperative management strategies to reduce the incidence of cardiovascular complications following cardiac and non-cardiac surgery^3,20–22^. Despite these trials, comparatively little is known about the metabolic and transcriptional changes following non-cardiac surgery.

In this study, we demonstrated that isolated thoracotomy surgery alters the cardiac transcriptome within the first two weeks after surgery (Fig. 1). In the absence of additional stressors, these changes disappear within 4 weeks after the procedure. Pathway enrichment analyses show a decrease in oxidative phosphorylation and energy supply (Fig. 1b,c). These changes might be mediated by decreased TEAD4 activity. Interestingly, the transcriptional changes within the first two weeks after surgery in our murine study coincide with clinical data reporting most cardiovascular events within the first two weeks^3^.

To our surprise, we also noted similarities in gene expression between the early phase after isolated thoracotomy surgery and decompensated heart failure (Fig. 5). Transcriptional similarities between heart failure and postoperative stress response may advocate perioperative heart failure therapy. Beta-blocker therapy in heart failure was associated with a partial reversal of transcriptional alterations^23^. Clinical studies on perioperative beta-blocker use, however, yielded ambiguous results^3,20^. This might be due to differences between substances regarding their pleiotropic effects and inter-individual response to treatment as well as to the difficulty of administering individualized medical treatment in the setting of a randomized controlled trial^24,25^.

Despite the above-mentioned transcriptional cardiac similarities between heart failure and postoperative stress-response, there are also apparent differences. Heart failure has a more pronounced impact on the cardiac transcriptome causing significant perturbation in multiple pathways^6,26^. This leads to profound metabolic changes in the heart as well as the liver, skeletal muscle and plasma. On the other hand, transcriptional changes following ITH seem to be well compensated as there was no significant metabolite accumulation or depletion detectable.

Conceptually, the downregulation of cardiac mitochondrial pathways might be a protective mechanism in response to systemic inflammatory stress in the absence of hypertrophic stimuli. Although sedoanalgesia changes cardiac gene expression for several days, to our knowledge, there is no evidence that this effect might last up to two weeks^27^. In addition, we observed the same changes in gene expression in both of our cohorts although controls of the second cohort were treated with sedoanalgesia and controls of the first cohort were not. Therefore, systemic effects associated with wound healing might be the most likely cause of these transcriptional alterations. The observed changes, however, do not lead to upregulation of cardiac inflammatory pathways.

This short-lived but well reproducible transcriptomic signature might prime the heart in order to reduce the harmful effects of cardiovascular events. Further research is required to improve our understanding of this novel finding. It would also be interesting to study whether laparotomy has the same transient effect after surgery.

Although microarray and metabolic panels represent an unbiased approach, there are some limitations to the study. Assumptions on different deregulated pathways are drawn primarily from transcriptional data. Protein expression might show different results. Furthermore, whole hearts were analyzed consisting mostly of cardiomyocytes. Thus, signals from other cardiac cells such as fibrocytes and fibroblasts might be underrepresented.

In conclusion, distinct transcriptional changes in the absence of significant metabolic alterations characterized by downregulated mitochondrial pathways are detectable 2 weeks following ITH and disappear within 4 weeks. These alterations are likely caused by increased Hippo signaling. To our knowledge, this work represents the first study investigating cardiac metabolic and transcriptional changes at different time points following isolated thoracotomy.

### Translational perspective

Thoracotomy is associated with significant cardiovascular complications. Despite clinical trials, little is known about the biomolecular mechanism leading to an increased cardiac vulnerability following surgery. Our study shows that post surgery transcriptional changes are characterised by a downregulation of mitochondrial pathways associated with increased Hippo signaling.

## Supplementary information

Supplementary information Supplementary Figure 1.

Supplementary Table 1.

Supplementary Table 1.

Supplementary Table 2.

Supplementary Table 2.

Supplementary Table 3.

Supplementary Table 4.

Supplementary Table 4.

Supplementary Table 5.
