# Supplementary material for: Cardiac transcriptional and metabolic changes following thoracotomy
Source: Sci Rep. 2020 Jun 15;10:9673. doi: 10.1038/s41598-020-66721-3 (PMC7295769; doi:10.1038/s41598-020-66721-3)
Supplement: Supplementary file 1 — Supplementary information Supplementary Figure 1. [file 41598_2020_66721_MOESM1_ESM.pdf]

Cardiac transcriptional and metabolic changes following thoracotomy

Markus B. Heckmann1, Ashraf Yusuf Rangrez2, Daniel Finke1, Andreas Jungmann1, Julia S. Kreußer1, Alexandra Rosskopf2, Nesrin Schmiedel2, Hugo A. Katus1, Norbert Frey2, Oliver J. Müller2*

1 Department of Internal Medicine III, Cardiology, Angiology & Pulmonology, Heidelberg University Hospital, Im Neuenheimer Feld 669, 69120 Heidelberg, Germany, and DZHK (German Center for Cardiovascular Research), partner site Heidelberg/Mannheim, Germany

2 Department of Internal Medicine III, University of Kiel, Arnold-Heller-Str. 3, 24105, Kiel, Germany, and DZHK (German Centre for Cardiovascular Research), Partner Site Hamburg/Kiel/Lübeck, Germany

Supplementary Figure

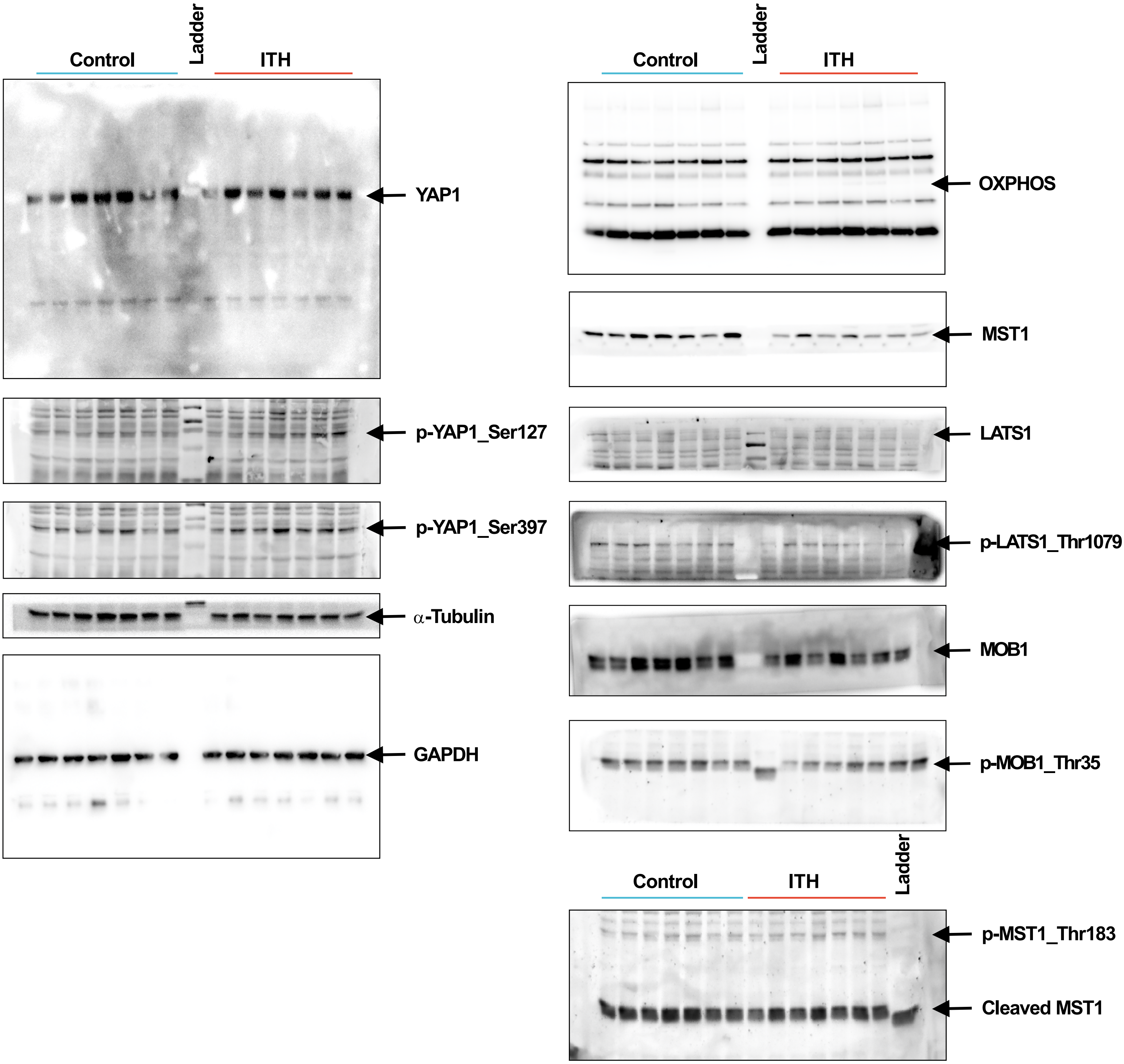

Supplementary Figure 1. Uncropped western blots with marked lanes.
