# Supplementary material for: Cardiac transcriptional and metabolic changes following thoracotomy
Source: Sci Rep. 2020 Jun 15;10:9673. doi: 10.1038/s41598-020-66721-3 (PMC7295769; doi:10.1038/s41598-020-66721-3)
Supplement: Supplementary file 2 — Supplementary Table 1. [file 41598_2020_66721_MOESM2_ESM.pdf]

Cardiac transcriptional and metabolic changes following thoracotomy

Markus B. Heckmann1, Ashraf Yusuf Rangrez2, Daniel Finke1, Andreas Jungmann1, Julia S. Kreußer1, Alexandra Rosskopf2, Nesrin Schmiedel2, Hugo A. Katus1, Norbert Frey2, Oliver J. Müller2*

1 Department of Internal Medicine III, Cardiology, Angiology & Pulmonology, Heidelberg University Hospital, Im Neuenheimer Feld 669, 69120 Heidelberg, Germany, and DZHK (German Center for Cardiovascular Research), partner site Heidelberg/Mannheim, Germany

2 Department of Internal Medicine III, University of Kiel, Arnold-Heller-Str. 3, 24105, Kiel, Germany, and DZHK (German Centre for Cardiovascular Research), Partner Site Hamburg/Kiel/Lübeck, Germany

Supplementary Information

Supplementary Table 1: Significantly deregulated genes 2 weeks after ITH surgery

ILMN_ID	Symbol	ratio 2W	ratio 4W	ratio 6W	p value 2W	p value 4W	p value 6W	FDR 2W	FDR 4W	FDR 6W
ILMN_2630946	APRT	1.3419	0.8782	0.9439	1.30E-05	0.162806472	0.41619315	0.010014458	0.687561628	0.87087984
ILMN_2725428	DNAJB10	1.2919	0.8623	0.9371	2.51E-05	0.079938727	0.31276326	0.0151705	0.563734891	0.824757365
ILMN_2683675	LOC100044324	1.257	1.0054	1.1317	1.64E-06	0.93285883	0.014277554	0.003033513	0.991384973	0.423250023
ILMN_1249905	DBNDD2	1.2468	0.8613	0.9254	0.000146681	0.068812499	0.214537084	0.040855963	0.535546797	0.755257434
ILMN_2709456	RBM42	1.2223	1.1337	1.0963	2.33E-06	0.031765009	0.040070111	0.003594584	0.426581092	0.513040918
ILMN_2853508	TMEM208	1.2139	0.9684	0.9006	2.06E-05	0.607919009	0.032018943	0.013438053	0.92452961	0.492435206
ILMN_1247099	SETD1A	1.2015	0.9085	1.0172	8.47E-06	0.091411473	0.693211465	0.008817138	0.584833578	0.955635614
ILMN_1218959	D11WSU99E	1.1978	0.9259	0.9957	5.73E-06	0.157987705	0.917955985	0.006618284	0.68436538	0.99106441
ILMN_1215147	LOC100044829	1.1893	1.034	1.0402	0.000148467	0.59985394	0.421356896	0.041105867	0.922076398	0.872416893
ILMN_1239698	PUF60	1.1884	0.9696	0.9641	1.40E-05	0.570455038	0.383171084	0.010273927	0.912384711	0.860138289
ILMN_1254562	ATP13A2	1.1683	0.8204	0.9308	9.49E-05	0.000636921	0.095060121	0.031554708	0.154996488	0.635729749
ILMN_2716719	B230333C21RIK	1.167	1.1014	1.1298	0.000153549	0.093113891	0.006594913	0.041762726	0.587586117	0.36595348
ILMN_1224651	2810405J04RIK	1.1642	0.9937	1.0132	0.000179015	0.91066862	0.762803373	0.045593278	0.987867656	0.967789384
ILMN_2737192	EIF3S4	1.1628	1.0138	0.9704	1.23E-05	0.771597624	0.408608992	0.010014458	0.963377673	0.869793817
ILMN_2850849	COG1	1.1614	1.0079	1.0478	9.10E-05	0.882310426	0.25453428	0.03092399	0.983269128	0.785798323
ILMN_2606130	PCGF1	1.1584	1.092	1.0352	0.000161136	0.108966187	0.408552546	0.04356974	0.617889331	0.869793817
ILMN_1219468	ZNFX1	1.1403	1.0761	0.9807	0.00010491	0.122826589	0.589983682	0.033970759	0.638272131	0.928017538
ILMN_1234177	9530050F08RIK	1.1325	1.0593	0.9292	4.60E-05	0.174822221	0.025982959	0.02091199	0.702060137	0.467480685
ILMN_2741231	ODZ3	1.1183	1.0321	0.9777	6.35E-05	0.416531801	0.449319309	0.025466617	0.859347434	0.885197311
ILMN_2677859	INSL6	1.1182	0.9542	0.9857	5.80E-05	0.225449442	0.625576632	0.024363735	0.751441122	0.938020065
ILMN_2656679	RYR3	1.1055	0.9522	1.0419	6.08E-05	0.160752631	0.125539459	0.024678054	0.686844579	0.672492062
ILMN_2701875	B3GALT1	1.0979	0.9313	1.0325	9.23E-05	0.035106457	0.21108397	0.03092399	0.440254211	0.751107399
ILMN_2493815	MTIF3	1.0955	0.9976	1.024	0.000191247	0.944140092	0.366054804	0.047798319	0.993125161	0.851755224
ILMN_1250020	MURR2	0.9244	0.9591	0.934	7.98E-05	0.133913417	0.001912817	0.029038624	0.655616765	0.311915454
ILMN_2653747	GABRA1	0.9095	1.0049	0.9991	9.12E-05	0.88450952	0.972315282	0.03092399	0.98356038	0.997523198
ILMN_2647972	COQ4	0.9068	1.0229	1.0323	7.20E-05	0.508621778	0.22866116	0.027077986	0.894392073	0.766130873
ILMN_1243441	SKP2	0.9057	0.996	0.9584	0.000139555	0.911270566	0.129540587	0.040049219	0.988144875	0.675489807
ILMN_1247372	LOC235971	0.893	1.0572	1.0215	2.30E-08	0.038079808	0.295243045	0.000212663	0.448600056	0.814485838
ILMN_2729834	CLYBL	0.8929	0.9927	0.973	3.15E-05	0.844561935	0.344080717	0.017051759	0.97771683	0.841669706
ILMN_2769844	BC006662	0.8838	0.9791	0.9787	3.21E-05	0.605745049	0.494242318	0.017075237	0.923574651	0.897047383
ILMN_2754074	LAPTM4B	0.8705	1.1212	0.9264	8.84E-05	0.022447507	0.045648498	0.030837293	0.382970862	0.530343147
ILMN_3144820	LOC329575	0.8658	0.985	1.0129	1.22E-05	0.736845228	0.710033895	0.010014458	0.956546631	0.959354349
ILMN_1245200	IFI205	0.8637	1.0673	1.0275	5.99E-05	0.200635287	0.484975519	0.024678054	0.726166243	0.893663841
ILMN_1260441	2610044O15RIK	0.8579	0.9454	0.9343	0.000123242	0.313968963	0.114688892	0.037988892	0.809279862	0.655345764
ILMN_1240683	CHMP2A	0.8568	1.1189	1.0471	3.05E-05	0.030774667	0.24284571	0.01681379	0.421969537	0.776787069
ILMN_1257503	TM9SF2	0.8559	1.0374	0.9756	1.27E-05	0.45268576	0.510230743	0.010014458	0.874827364	0.902532889
ILMN_1249407	MRPL39	0.8545	1.0178	0.9277	7.34E-05	0.748495463	0.078666627	0.027375855	0.958685209	0.604916403
ILMN_2647484	PRKD1	0.8502	0.9723	0.9444	1.83E-06	0.540870567	0.107647411	0.003140173	0.902889464	0.651467225
ILMN_2955104	NACA	0.8441	0.9922	0.989	0.000116512	0.898550409	0.813364235	0.036178269	0.985723528	0.972758364
ILMN_1236357	4732479N06RIK	0.8394	0.8927	0.9741	1.21E-06	0.021770443	0.481124756	0.002424129	0.379177282	0.892374477
ILMN_2890238	KTN1	0.838	1.1416	1.004	0.000141957	0.044045499	0.936695278	0.040049219	0.472513167	0.99325744
ILMN_2503188	PPL	0.8364	0.9455	0.9609	9.18E-05	0.378438019	0.41447868	0.03092399	0.844632947	0.870785656
ILMN_1246340	9330185J12RIK	0.836	1.0299	1.0166	0.000116585	0.649205432	0.740677684	0.036178269	0.935701731	0.964344649
ILMN_2504686	MT-ATP6	0.8313	0.8827	1.0608	1.81E-05	0.037871372	0.19613448	0.012095774	0.448134081	0.739876801
ILMN_1256668	FIG4	0.8302	1.0659	0.8182	0.000167912	0.357078308	0.000313626	0.044876108	0.835036856	0.238655319
ILMN_1234781	APIP	0.8283	0.9959	0.9113	0.00011478	0.951380531	0.078306456	0.036102646	0.994277667	0.604450016
ILMN_2506757	LOC100048280	0.8271	0.9834	0.8257	0.00013741	0.809841686	0.000586289	0.040049219	0.972033332	0.268398387
ILMN_2650502	GDAP10	0.8258	0.8611	0.908	4.86E-07	0.004306641	0.015408097	0.001478233	0.246023465	0.426632288
ILMN_3005058	ETFDH	0.8251	1.1009	0.9742	3.63E-06	0.091040633	0.545887191	0.004938671	0.583900513	0.914395513
ILMN_2608613	D10WSU52E	0.8238	1.0826	1.015	4.72E-05	0.230569607	0.768929861	0.021182313	0.75555258	0.968627749
ILMN_1239002	PAFAH1B1	0.8233	1.0826	1.0196	5.44E-06	0.175475219	0.663886314	0.006618284	0.702060137	0.948716534
ILMN_2498779	MSL2L1	0.8222	0.9323	0.9312	1.26E-05	0.255575594	0.133579476	0.010014458	0.774118059	0.680210817
ILMN_1213808	LOC270186	0.8214	1.1244	1.1064	0.000112275	0.101482741	0.066388567	0.035801797	0.606618846	0.575241311
ILMN_2684378	4930534B04RIK	0.811	0.9842	0.9738	0.000127381	0.834320702	0.649702152	0.038748236	0.975883598	0.944644747
ILMN_2426691	MT-CO2	0.8084	0.8685	0.9946	0.000177098	0.078499068	0.929168177	0.045491659	0.5606272	0.99283309
ILMN_1224589	TMEM77	0.8079	1.0744	1.0464	6.39E-05	0.333623864	0.424568805	0.025466617	0.822304055	0.874074075

ILMN_2625167	EIF4G2	0.8058	1.0142	1.0972	3.89E-05	0.845855062	0.098652972	0.018560079	0.978013967	0.641549573
ILMN_3145782	ZFP97	0.8054	1.0254	0.9304	3.62E-05	0.729095974	0.195991529	0.018376213	0.955133636	0.739699644
ILMN_1221592	SEC11C	0.805	0.78	0.86	0.000104282	0.002057856	0.013463917	0.033970759	0.205757726	0.421331255
ILMN_1215639	COMMD1	0.8045	1.2213	1.0489	6.54E-05	0.009874612	0.410878573	0.025612726	0.301998913	0.869868095
ILMN_1218605	NFKBIA	0.8022	0.866	0.9522	8.71E-06	0.036382823	0.346506669	0.008817138	0.444682156	0.842202572
ILMN_2596761	1110005A23RIK	0.8008	0.9771	0.8807	9.00E-05	0.768897121	0.038675643	0.03092399	0.962968426	0.509040864
ILMN_1241268	SNX3	0.797	1.1279	1.037	6.66E-05	0.13032438	0.548445435	0.025715871	0.650664977	0.915112787
ILMN_2678400	C920006C10RIK	0.7969	1.1418	1.0413	3.03E-06	0.047239789	0.424569709	0.004520468	0.483022146	0.874074075
ILMN_2702857	OSBPL1A	0.7962	1.1561	0.8838	4.63E-06	0.034827929	0.019609172	0.005781381	0.439046021	0.447737927
ILMN_2469264	AFF1	0.7962	0.908	0.8772	4.52E-05	0.213830722	0.029846694	0.02091199	0.739826456	0.487456239
ILMN_2621304	MTERFD1	0.7961	0.9615	0.9548	2.84E-05	0.601465465	0.422699998	0.016191381	0.922289433	0.873101621
ILMN_2701093	JAK1	0.7942	1.1394	0.863	3.17E-05	0.090748149	0.01396659	0.017051759	0.583386965	0.423250023
ILMN_1247947	SQRDL	0.7932	0.9649	0.9546	0.000149959	0.675382124	0.477845767	0.041271788	0.941272103	0.891945663
ILMN_2637630	BC013529	0.7927	0.9709	0.9638	0.000164993	0.731870211	0.576599291	0.044353307	0.955569218	0.924706789
ILMN_1223496	LOC100046025	0.7926	1.0086	1.0428	3.84E-05	0.912103903	0.484034764	0.018560079	0.988144875	0.893115251
ILMN_2706587	TJP1	0.7914	0.8945	0.7875	8.70E-05	0.18101856	0.000346726	0.030837293	0.707084155	0.238655319
ILMN_2661733	HADHSC	0.7905	1.0612	1.0401	3.37E-08	0.287666746	0.358331211	0.000260056	0.795457649	0.847424517
ILMN_2698494	NFU1	0.79	0.9795	0.8867	2.47E-05	0.78749852	0.044682431	0.0151705	0.967427575	0.526121397
ILMN_2675464	ANKRD17	0.7896	1.0543	0.9658	2.59E-05	0.49424192	0.55732678	0.0151705	0.8909837	0.918137207
ILMN_2740622	OGN	0.7879	1.0745	0.9367	1.82E-06	0.288019105	0.208097811	0.003140173	0.795732163	0.750290428
ILMN_2531572	LOC225927	0.7876	0.9643	0.9533	1.00E-05	0.623042829	0.400154632	0.009823123	0.92740819	0.866484501
ILMN_2739287	DAZAP2	0.7876	0.973	0.9111	0.000142389	0.754074262	0.168703962	0.040049219	0.959629575	0.715759217
ILMN_2671601	SELK	0.7874	1.0792	0.9398	0.000188685	0.395274311	0.366582916	0.047414266	0.849979819	0.851980214
ILMN_1225118	LOC100045280	0.7866	0.8374	0.9839	8.63E-05	0.039691441	0.80370753	0.030837293	0.454485676	0.972402737
ILMN_2433685	ENTPD5	0.7851	0.908	0.8418	5.12E-05	0.245330512	0.008160558	0.022128779	0.766258768	0.380693281
ILMN_1215246	MCCC1	0.7827	1.0861	0.8467	6.07E-05	0.331110735	0.012368943	0.024678054	0.820492366	0.412130808
ILMN_2754636	LOC639931	0.7827	1.2978	1.0293	0.000142918	0.004871632	0.674976044	0.040049219	0.257405491	0.950492265
ILMN_1214918	LOC546015	0.7826	0.8996	0.7269	0.00018403	0.250156938	2.29E-05	0.046497273	0.768543428	0.109518476
ILMN_2589039	COX6C	0.7826	0.8145	0.8942	5.76E-05	0.017373327	0.087497048	0.024363735	0.353267849	0.62328024
ILMN_2802611	TM9SF2	0.7801	1.0455	0.9742	0.000169842	0.629555832	0.711737717	0.044876108	0.929764844	0.959464669
ILMN_2937320	SCHIP1	0.7791	1.195	1.024	5.68E-05	0.041040999	0.718820431	0.024307621	0.460250232	0.961282116
ILMN_2513570	AW123240	0.7787	1.1393	0.955	8.82E-05	0.144466285	0.499129421	0.030837293	0.668376799	0.898004797
ILMN_2706827	SETX	0.7771	1.0279	0.8999	1.38E-05	0.72959596	0.087125094	0.010273927	0.955310767	0.622198066
ILMN_2771074	HNRNPA2B1	0.7767	1.0975	0.9726	4.61E-05	0.27979014	0.673211453	0.02091199	0.789746012	0.950479518
ILMN_2881296	TMEM66	0.7753	0.9566	0.8195	0.000172997	0.639633694	0.007589879	0.045041861	0.932868912	0.376695519
ILMN_2635976	GLTSCR2	0.7745	0.8593	0.7907	4.93E-05	0.085023435	0.00076406	0.021723398	0.572596335	0.281928804
ILMN_1243273	PCMT1	0.7744	1.1335	0.9718	6.73E-05	0.161810505	0.675460812	0.025715871	0.687246806	0.950691942
ILMN_2680262	POLR2G	0.7744	0.9942	0.8598	1.77E-05	0.943596836	0.018359779	0.012095774	0.993037144	0.440573994
ILMN_2997382	MTERFD3	0.7734	1.2122	0.893	1.28E-05	0.019403964	0.070716332	0.010014458	0.365520504	0.586705735
ILMN_1214664	GLRX2	0.7733	1.0508	0.9238	2.12E-05	0.551682869	0.216540054	0.013631883	0.906504393	0.756027076
ILMN_2707245	RFWD2	0.7721	0.989	0.9691	1.08E-05	0.890068817	0.6104944	0.009823123	0.984336193	0.933747797
ILMN_2743668	LIAS	0.7713	1.0596	0.8537	2.95E-05	0.499953483	0.018427518	0.01664561	0.891777967	0.440573994
ILMN_2881272	AIFM1	0.7703	1.0188	0.9084	3.50E-05	0.830249335	0.153347475	0.018173304	0.975595452	0.7033357
ILMN_2717599	ATP5F1	0.7633	0.8113	0.7922	4.44E-05	0.024792328	0.001400847	0.020722249	0.391637464	0.307970453
ILMN_2596297	DDT	0.762	0.9451	0.7614	0.000136705	0.570184771	0.000629258	0.040049219	0.912270056	0.279759789
ILMN_1222174	NDUFA12L	0.7616	0.9299	0.7755	0.000173064	0.473326896	0.001623494	0.045041861	0.883213234	0.309816685
ILMN_2696620	RWDD4A	0.7576	1.0231	0.9407	1.38E-06	0.767645166	0.305315662	0.002651142	0.962590772	0.820319616
ILMN_2985282	PECI	0.7553	1.0023	0.8575	0.000114482	0.981893521	0.051078137	0.036102646	0.997753428	0.542875702
ILMN_2982200	MRPL13	0.7513	0.9609	0.8683	0.000129977	0.701997962	0.080464344	0.039048455	0.947579482	0.60900896
ILMN_1233665	2610019N19RIK	0.7507	1.1604	0.9792	1.16E-05	0.099852263	0.759010737	0.009934852	0.602331258	0.967351548
ILMN_1252431	OSBP	0.7484	1.2252	1.1114	9.95E-05	0.053077566	0.186296102	0.032864126	0.49870909	0.731137722
ILMN_1243095	VBP1	0.7461	0.9771	0.8371	1.80E-05	0.804974249	0.015625799	0.012095774	0.971156792	0.426632288
ILMN_2677882	TANC1	0.7443	0.918	0.8533	5.91E-06	0.336382406	0.022302995	0.006660298	0.823231529	0.458034038
ILMN_2551741	0610010I05RIK	0.7434	1.0121	0.9667	3.66E-05	0.903556548	0.656673292	0.018406946	0.986917221	0.946707639
ILMN_2632509	PPP1R14C	0.7408	1.1125	0.8482	0.000141094	0.333489727	0.054127256	0.040049219	0.822304055	0.550887508
ILMN_2590950	AGTRAP	0.7406	0.7666	0.9079	0.000126343	0.017133326	0.250638138	0.038686878	0.352536448	0.783609459
ILMN_2623859	ABCC9	0.7401	1.032	0.829	0.000131746	0.773751208	0.028602117	0.039048455	0.963962898	0.480951633
ILMN_2775381	TDE2	0.739	0.9023	0.8758	1.33E-07	0.178078078	0.025210654	0.00077136	0.704642544	0.464544507
ILMN_2699028	BC043118	0.7388	0.9595	0.7639	6.51E-05	0.693900345	0.001317176	0.025612726	0.945093081	0.304163155
ILMN_2660766	CD59B	0.7387	1.1849	1.0885	7.57E-05	0.113427165	0.299794902	0.027992813	0.623766359	0.817119599
ILMN_1219002	USMG5	0.7359	0.9297	0.8998	0.000169849	0.522853224	0.229261481	0.044876108	0.898200601	0.766531427
ILMN_1258789	YTHDF3	0.7336	1.073	0.9274	1.20E-06	0.412651759	0.25448578	0.002424129	0.857687721	0.785793212
ILMN_2421751	KIF1C	0.7328	1.0831	1.031	1.77E-05	0.423010533	0.689398151	0.012095774	0.861290405	0.954563584
ILMN_2891176	MDH1	0.7325	1.2376	1.1177	9.45E-07	0.014466859	0.09207476	0.002299212	0.33545358	0.630838122
ILMN_2507810	MTDNA_ND5	0.7322	1.0478	1.0145	3.79E-05	0.655274616	0.857816512	0.018560079	0.93722997	0.980873131
ILMN_2432886	LOC100043126	0.7315	1.1479	0.9442	3.56E-05	0.189207183	0.474055812	0.018272645	0.714904436	0.891526577
ILMN_1225386	2300009A05RIK	0.7314	0.8856	0.758	2.56E-05	0.23714702	0.000726691	0.0151705	0.762383064	0.281928804
ILMN_1232951	EG434404	0.731	0.8865	0.8824	2.03E-05	0.235488725	0.109842675	0.013399256	0.760754684	0.65296937
ILMN_2431965	PALLD	0.7294	1.2991	1.0648	0.000131318	0.025553394	0.477149033	0.039048455	0.395022492	0.891798825
ILMN_1239860	1810035L17RIK	0.7264	0.8642	0.8518	8.87E-05	0.200477103	0.068290505	0.030837293	0.725934671	0.580645109
ILMN_2690203	R3HDM2	0.7254	1.0567	0.8062	5.00E-05	0.614472026	0.012284586	0.021824062	0.925316825	0.411803379
ILMN_2434853	MTDNA_ND2	0.7247	0.9064	0.8896	3.48E-05	0.361007522	0.158466571	0.018173304	0.836933497	0.7078408
ILMN_2845208	LRPPRC	0.7244	1.2757	0.9627	8.56E-05	0.035738033	0.663763599	0.030837293	0.44253333	0.948699769
ILMN_2500997	GLRX3	0.7237	1.07	0.8428	3.27E-06	0.473318356	0.020367765	0.004582185	0.883213234	0.453055484
ILMN_1236079	GM129	0.7219	1.1066	1.3887	0.000173399	0.404485854	0.000723061	0.045041861	0.854173628	0.281928804

ILMN_2614246	DNAJB4	0.716	1.0513	1.0168	6.68E-05	0.666758148	0.851822343	0.025715871	0.939201285	0.979444782
ILMN_1247054	CLASP1	0.7159	1.0936	0.6744	1.07E-05	0.389823331	5.09E-06	0.009823123	0.84799679	0.058876662
ILMN_2733330	RPS3A	0.7153	1.0042	0.7586	4.40E-05	0.970451541	0.002177591	0.020722249	0.99619839	0.319122295
ILMN_2774690	LOC677317	0.7138	0.9188	0.8659	2.17E-05	0.438608129	0.088924991	0.013750874	0.86723143	0.62514474
ILMN_2428506	AHCYL1	0.7104	1.0147	0.8936	8.27E-09	0.849019362	0.059292119	9.56E-05	0.979004995	0.561053663
ILMN_1247553	SEPP1	0.7081	0.9373	0.6905	0.000153299	0.610753432	0.000306801	0.041762726	0.924911709	0.238655319
ILMN_1259127	BCAS2	0.7079	1.0034	0.9331	2.39E-07	0.969505278	0.311070088	0.001229123	0.996164509	0.824010367
ILMN_1215212	RHOB	0.7075	0.8667	0.9794	2.48E-05	0.207575243	0.809619017	0.0151705	0.733924183	0.972758364
ILMN_2776056	RASSF3	0.7057	1.3017	0.8938	0.000105063	0.037991684	0.244284719	0.033970759	0.448600056	0.777635566
ILMN_2937261	MOD1	0.7052	1.2656	0.8918	5.92E-05	0.053813221	0.217803896	0.024645954	0.500832131	0.756920663
ILMN_2706468	2310002F18RIK	0.702	1.1863	1.0196	1.20E-06	0.084560961	0.796440064	0.002424129	0.572065013	0.971235711
ILMN_1231096	2310036D22RIK	0.7012	1.122	1.0579	2.83E-05	0.326175736	0.530500266	0.016191381	0.817440845	0.909788439
ILMN_2526572	LOC545369	0.7006	0.8318	0.7954	1.45E-05	0.105034034	0.009697676	0.010503393	0.613160682	0.390280392
ILMN_2622761	PCCB	0.6985	1.1247	0.9666	6.18E-07	0.224666244	0.645249725	0.001586252	0.75065551	0.943330985
ILMN_1227012	NDUFB4	0.6977	0.96	0.9278	0.000108161	0.752123319	0.451196337	0.034729355	0.959131026	0.885704774
ILMN_1258515	PAIP2	0.6976	0.9435	0.7523	7.31E-06	0.595359344	0.00113958	0.007977333	0.92055469	0.287832333
ILMN_2591440	CCRN4L	0.6968	0.8895	1.0317	2.33E-06	0.25948828	0.694313754	0.003594584	0.777472757	0.95583521
ILMN_2798993	NR1D2	0.6944	0.8736	0.8596	4.79E-05	0.278071267	0.115292426	0.021284293	0.789671588	0.655934925
ILMN_2454710	PPM1K	0.6933	1.2792	1.0003	1.74E-05	0.03839846	0.997119931	0.012095774	0.449241103	0.999858077
ILMN_2592881	JAM2	0.691	0.9024	0.7555	6.94E-05	0.42640629	0.005858366	0.026285209	0.862285194	0.356860889
ILMN_2614728	ACADSB	0.6896	0.8913	0.7253	0.000179466	0.407266959	0.003443679	0.045593278	0.855708858	0.333260382
ILMN_1230454	HADHB	0.6867	1.3957	0.9136	3.86E-05	0.00993949	0.351831961	0.018560079	0.302151341	0.84412862
ILMN_3132666	OSBPL8	0.685	1.0383	0.9247	1.07E-05	0.749248274	0.386537069	0.009823123	0.958685209	0.861693848
ILMN_2447373	ABCD3	0.6806	0.901	0.7944	0.000192449	0.470650692	0.040547074	0.047840249	0.882585389	0.514126669
ILMN_2591917	AKR1B3	0.6781	1.1322	0.9462	4.50E-06	0.282343913	0.531428922	0.005781381	0.79146139	0.91020946
ILMN_1214703	NME7	0.677	0.8728	0.9245	0.000138213	0.341878668	0.474131007	0.040049219	0.825497766	0.891589205
ILMN_1221290	LOC636952	0.6728	0.9037	0.7705	1.15E-05	0.413814775	0.007465217	0.009934852	0.85855426	0.376695519
ILMN_2670713	CYFIP2	0.6703	0.929	0.7533	7.73E-05	0.60029669	0.010341818	0.028371017	0.922092853	0.393601171
ILMN_1230043	LRRC39	0.6671	1.3734	1.1893	3.04E-05	0.020177907	0.094702366	0.01681379	0.368523396	0.634994237
ILMN_2494747	HUWE1	0.6631	0.6863	0.7146	8.77E-06	0.00384613	0.000876346	0.008817138	0.241951413	0.283353911
ILMN_2605004	NDUFB9	0.6621	1.1096	0.7653	1.13E-05	0.419032619	0.008205526	0.009934852	0.860383532	0.380693281
ILMN_2671689	COX7B	0.6616	0.9707	0.9975	2.84E-07	0.780712559	0.976075251	0.0012323	0.966237833	0.997750603
ILMN_2527341	LOC381365	0.6608	0.9362	0.8471	2.57E-05	0.626546981	0.113761055	0.0151705	0.928850828	0.655112092
ILMN_2663694	NDUFS4	0.6534	0.9188	0.8091	1.39E-05	0.528704843	0.042767676	0.010273927	0.89904479	0.520959797
ILMN_1258158	ALDH6A1	0.6527	1.1323	0.8454	3.86E-05	0.386487822	0.129373231	0.018560079	0.846697753	0.675318474
ILMN_1239143	SDHD	0.6436	1.1789	0.9468	3.17E-06	0.201487219	0.578508659	0.004577692	0.726803289	0.925290477
ILMN_3116570	PDHB	0.6428	1.7132	1.0499	0.000142638	0.001392771	0.695087955	0.040049219	0.186301493	0.955969998
ILMN_2773099	PPA2	0.6393	1.361	1.0697	2.03E-06	0.017612591	0.490514691	0.003347589	0.354838072	0.895789159
ILMN_2617335	NDUFB5	0.6358	0.9246	0.797	3.20E-07	0.506866023	0.014215033	0.0012323	0.893960528	0.423250023
ILMN_1217159	LMO7	0.6333	0.8651	0.8356	0.000175069	0.39524214	0.171289028	0.045221715	0.849979819	0.716676541
ILMN_1252470	SORBS1	0.6323	0.8425	0.8836	1.20E-06	0.179769179	0.206199475	0.002424129	0.705977166	0.748719654
ILMN_2771754	FH1	0.6319	0.8465	0.8192	3.81E-07	0.169298492	0.033783624	0.001257386	0.693931446	0.496520481
ILMN_2640122	PMPCB	0.6282	1.0799	0.7689	1.97E-10	0.402282526	0.000369914	9.12E-06	0.853570158	0.238655319
ILMN_2821850	NAMPT	0.6163	0.7018	0.7646	0.000130673	0.047870738	0.050453295	0.039048455	0.484177373	0.541488164
ILMN_1237846	LOC100044087	0.6063	1.2355	0.9164	1.08E-05	0.176610533	0.464835496	0.009823123	0.703172411	0.888534508
ILMN_3155380	CYCS	0.5895	0.9964	0.8266	4.30E-06	0.981601752	0.115015099	0.005678835	0.997720823	0.65583437
ILMN_2836607	ART3	0.5845	1.1647	1.1792	6.79E-09	0.204781684	0.075726002	9.56E-05	0.730859345	0.600669874
ILMN_2587859	LOC668837	0.5831	0.8783	0.8366	1.33E-07	0.33838578	0.088656	0.00077136	0.824381858	0.62514474
ILMN_2691460	NDUFA5	0.583	1.2307	0.9482	5.44E-07	0.152103271	0.629955684	0.001478233	0.679223017	0.939197088
ILMN_1227815	IDH3A	0.5788	1.8013	1.1275	7.42E-06	0.000712302	0.348552783	0.007977333	0.160657161	0.843048151
ILMN_2703657	DLD	0.5666	1.1948	0.9847	3.53E-07	0.233313614	0.892660438	0.001256752	0.758259938	0.986567099
ILMN_1227412	HISPPD1	0.5614	0.8528	0.7528	5.67E-06	0.359021231	0.035466743	0.006618284	0.836095478	0.500769704
ILMN_2419660	MTDNA_ND4L	0.535	0.8899	0.9098	3.18E-07	0.474208444	0.449790351	0.0012323	0.8839279	0.885365277
ILMN_2633229	ATP5A1	0.5338	0.9145	0.8265	1.45E-09	0.499968716	0.063694508	3.35E-05	0.891777967	0.571477679
ILMN_2512204	MT-ND4L	0.4503	0.8936	0.9424	5.13E-07	0.596469915	0.715577056	0.001478233	0.92085836	0.960639236
