# Supplementary material for: Cardiac transcriptional and metabolic changes following thoracotomy
Source: Sci Rep. 2020 Jun 15;10:9673. doi: 10.1038/s41598-020-66721-3 (PMC7295769; doi:10.1038/s41598-020-66721-3)
Supplement: Supplementary file 4 — Supplementary Table 2. [file 41598_2020_66721_MOESM4_ESM.pdf]

Cardiac transcriptional and metabolic changes following thoracotomy

Markus B. Heckmann1, Ashraf Yusuf Rangrez2, Daniel Finke1, Andreas Jungmann1, Julia S. Kreußer1, Alexandra Rosskopf2, Nesrin Schmiedel2, Hugo A. Katus1, Norbert Frey2, Oliver J. Müller2*

1 Department of Internal Medicine III, Cardiology, Angiology & Pulmonology, Heidelberg University Hospital, Im Neuenheimer Feld 669, 69120 Heidelberg, Germany, and DZHK (German Center for Cardiovascular Research), partner site
2 Department of Internal Medicine III, University of Kiel, Arnold-Heller-Str. 3, 24105, Kiel, Germany, and DZHK (German Centre for Cardiovascular Research), Partner Site Hamburg/Kiel/Lübeck, Germany

Supplementary Information

Supplementary Table 2: Metabolic Profile 2,4 and 6 weeks after ITH surgery

MET_CHEM_NO	Effect_name	ratio 2W	ratio 4W	ratio 6W	p value 2W	p value 4W	p value 6W	FDR 2W	FDR 4W	FDR 6W
39210072	Aspartate	1.3474	0.8441	1.0666	0.03493671	0.215552043	0.640632501	0.6838209	0.568491235	0.909415222
39210088	Glutamate	1.0119	1.1373	1.1153	0.904389696	0.186356411	0.268469351	0.987924779	0.557497683	0.720167768
39210073	Phenylalanine	1.0735	0.8058	0.8627	0.402550586	0.011020848	0.082168565	0.963288743	0.234208529	0.519630104
19210035	Tryptophan	1.1859	0.8537	1.0784	0.060277237	0.074702148	0.397432625	0.723327965	0.400279356	0.806425954
39210160	Tyrosine	1.094	0.789	0.7386	0.536615019	0.099172857	0.038961017	0.963288743	0.429133622	0.47122359
19210043	Arginine	1.2492	0.8489	1.2678	0.055541021	0.147575229	0.040379169	0.723327965	0.484186328	0.47122359
39210145	Asparagine	1.1203	0.8721	0.6054	0.306288606	0.227474613	3.55E-05	0.963288743	0.581611226	0.015994329
39210045	Glutamine	0.9029	0.7672	1.0003	0.84183454	0.586757401	0.999445465	0.987924779	0.854501069	0.999445465
39210466	Histidine	0.9865	0.8568	0.7357	0.938010399	0.368728258	0.081270213	0.987924779	0.700145731	0.519630104
39210013	Lysine	1.146	0.9789	0.7394	0.383586746	0.888789332	0.054769924	0.963288743	0.980282352	0.47122359
39210074	Isoleucine	1.2495	0.9099	1.4507	0.03617157	0.357951699	0.000625991	0.6838209	0.694282674	0.14084789
39210075	Leucine	1.1278	0.857	1.1972	0.147409593	0.059111671	0.030758905	0.881182388	0.380003601	0.47122359
39210070	Valine	1.1584	0.9723	1.2901	0.071200994	0.721575651	0.002143674	0.723327965	0.927740122	0.20879708
39210003	Alanine	1.1157	1.0081	0.9712	0.097752029	0.900148369	0.653752015	0.785507374	0.980876989	0.91933877
39210004	Glycine	1.0443	0.9172	0.9525	0.458242304	0.133890862	0.40287932	0.963288743	0.47818165	0.809355777
39210079	Proline	1.0095	0.9069	0.8448	0.940530891	0.431825111	0.183172397	0.987924779	0.756114008	0.67102404
39210052	Serine	1.1503	1.1046	0.7737	0.164623253	0.311652724	0.011779006	0.881182388	0.659016186	0.378610908
39210006	Threonine	1.2676	0.943	0.8705	0.007366204	0.48643327	0.108262539	0.414348997	0.78976672	0.572592799
39210010	Cysteine (additional: Cystine)	1.0374	0.8701	1.0048	0.742978511	0.207208162	0.96534479	0.967341023	0.568491235	0.995804743
39210008	Methionine	1.1936	1.0713	0.9077	0.037566293	0.40168096	0.246574465	0.6838209	0.730903574	0.711272496
19210230	Taurine	1.0656	0.9653	1.0441	0.099852117	0.345899822	0.257367401	0.788306188	0.693783042	0.713352247
19215429	1-Methylhistidine (additional: 3-Methylhistidine)	1.1558	0.6664	0.8408	0.362532338	0.006626549	0.241417738	0.963288743	0.234208529	0.709739666
19215421	2-Methylserine	1.3423	1.0002	0.8336	0.009457015	0.998352634	0.079386489	0.472850759	0.998352634	0.517737973
19215433	epsilon-Acetyllysine	1.0383	1.0071	0.8761	0.744387559	0.946428183	0.218853181	0.967341023	0.989177255	0.693716712
19215431	N-Acetylaspartate	0.8909	0.8857	1.2529	0.276976221	0.212170666	0.024455174	0.957338027	0.568491235	0.47122359
19215436	N-Phenylacetylglycine	1.0776	1.2958	0.889	0.842649959	0.452710158	0.736329222	0.987924779	0.777555616	0.949421632
39211701	2-Hydroxyglutaric acid	1.0579	0.9583	0.9019	0.605713427	0.690042302	0.341967091	0.963288743	0.910613009	0.749017353
39210009	5-Oxoproline (additional: Folic acid, Glutamate, Glutamine)	1.0246	0.9836	0.9487	0.68301235	0.776009747	0.374802361	0.963288743	0.959389312	0.780838252
39211135	beta-Alanine (additional: Pantothenic acid)	1.2543	1.2307	1.0462	0.042732105	0.057528855	0.679838801	0.70111745	0.375188182	0.927488888
39210071	Homoserine	0.9659	1.0376	1.0158	0.651595829	0.62358903	0.837202908	0.963288743	0.876734562	0.96811619
19215422	Pipecolic acid	1.1287	1.0189	0.8404	0.277480455	0.85393562	0.095277357	0.957338027	0.978653902	0.549677061
39210437	trans-4-Hydroxyproline	1.1405	1.545	1.3619	0.380881177	0.004058779	0.040983699	0.963288743	0.234208529	0.47122359
19210325	Creatine	1.0288	0.9883	1.0313	0.483346718	0.766615901	0.443838355	0.963288743	0.958269877	0.834441552
19210321	Creatinine	1.0324	1.0038	1.0986	0.838613714	0.98021215	0.546295753	0.987924779	0.998352634	0.877975318
19210362	Phosphocreatine	1.2288	1.6457	1.2025	0.32104123	0.016250865	0.371076657	0.963288743	0.265637387	0.778587307
19215451	Betaine	1.3418	1.277	0.8781	0.227195916	0.271835564	0.563468889	0.951497416	0.641212968	0.882941802
39211980	N,N-Dimethylglycine	1.2933	1.2277	0.7002	0.064933617	0.13101023	0.011001631	0.723327965	0.475440351	0.378610908
19215423	Proline betaine	0.9536	1.1525	1.07	0.611805116	0.100873515	0.437216224	0.963288743	0.429133622	0.834441552
19215386	S-Adenosylhomocysteine	0.8654	0.743	1.0225	0.242513496	0.010175399	0.845843326	0.951497416	0.234208529	0.96811619
39211926	S-Adenosylmethionine	1.0758	1.0728	1.0576	0.382531777	0.390976428	0.500246692	0.963288743	0.730903574	0.859202334
19215468	Indole-3-lactic acid	1.2551	0.8121	1.0837	0.076867382	0.076497832	0.495664158	0.723327965	0.400279356	0.857880273
19210316	O-Phosphotyrosine	1.043	1.0913	0.6887	0.882717948	0.754641716	0.191549734	0.987924779	0.95658809	0.675776419
19210159	Citrulline	1.0896	0.9026	1.0127	0.310588823	0.217289983	0.880220172	0.963288743	0.568491235	0.969056278
39210046	Ornithine (additional: Arginine, Citrulline)	1.112	1.0468	0.8425	0.443482327	0.735676168	0.214727205	0.963288743	0.940495101	0.693716712
29210428	Urea	0.983	1.0131	1.0736	0.854048785	0.886256576	0.443806922	0.987924779	0.980282352	0.834441552
39210021	Maltose	1.1793	1.872	0.476	0.646993613	0.063331036	0.03121008	0.963288743	0.390396799	0.47122359
39210036	Glucose	0.928	1.5668	1.0407	0.863957423	0.295200762	0.92657813	0.987924779	0.651178151	0.988057247
59210966	Hexoses (additional: Glucose, myo-Inositol)	0.8259	1.0008	0.946	0.305377467	0.996678788	0.763813297	0.963288743	0.998352634	0.954126516
39210028	Mannose	1.1129	1.3939	0.9374	0.708552386	0.238311159	0.820081555	0.963288743	0.599106266	0.963542296
39211909	1,2-Anhydroribose (additional: Nucleosides)	1.1865	0.754	0.9475	0.169764153	0.022287713	0.660852019	0.881182388	0.288796737	0.924125868
39210100	Maltitol	1.045	0.9423	1.028	0.625498192	0.501345563	0.757935155	0.963288743	0.791598258	0.954126516
39210014	myo-Inositol	1.0555	1.0783	1.0862	0.555282818	0.400902029	0.364171242	0.963288743	0.730903574	0.773702424
39210620	scyllo-Inositol	1.0092	1.0011	1.0501	0.925238171	0.990929755	0.616111447	0.987924779	0.998352634	0.9003228
59210792	Pentose acids	0.9308	0.9352	1.1322	0.391770539	0.413854284	0.138080405	0.963288743	0.73878449	0.629175857
39210044	Maltotriose	1.242	2.402	0.4507	0.619500274	0.043445815	0.069488131	0.963288743	0.342993275	0.510504979
29210176	Hentriacontane	1.1059	1.0162	1.0204	0.256480771	0.841502316	0.804694075	0.951497416	0.975969181	0.963285466
269210292	Cholic acid	0.8246	1.4553	0.7397	0.602516673	0.30222146	0.413322591	0.963288743	0.654211684	0.818944971
19210396	Taurocholic acid	1.5913	0.0933	0.9413	0.707033812	0.053354952	0.960666749	0.963288743	0.375188182	0.995804743
69210614	Ceramide (d18:1,C24:0)	0.9956	1.0567	1.2412	0.97618142	0.685703742	0.121984301	0.999882889	0.910613009	0.610107695
69210115	Ceramide (d18:1,C24:1) (additional: Ceramide (d18:2,C24:0))	0.9522	0.9231	1.0932	0.600053256	0.35011885	0.305696793	0.963288743	0.693783042	0.732507549
29210522	Cholestenol No 02	0.9865	1.0468	1.0261	0.885297043	0.620022789	0.783027648	0.987924779	0.876734562	0.958891859
19210401	Cholesterol, free	1.085	1.0154	1.0751	0.169656246	0.776345393	0.188909094	0.881182388	0.959389312	0.674675335
29210019	Cholesterol, total	1.0857	1.037	1.0624	0.29849802	0.637932301	0.440089438	0.963288743	0.888760171	0.834441552
29210850	Dihydrocholesterol	1.0951	0.8448	1.0048	0.40533645	0.117456586	0.964465682	0.963288743	0.451756099	0.995804743
19210405	Cholesterylester C18:2	1.4921	0.7415	0.8626	0.073996259	0.14227749	0.472512746	0.723327965	0.484186328	0.850522943
19210406	Cholesterylester C20:4	1.8279	0.6429	0.7346	0.036883015	0.092334697	0.244465885	0.6838209	0.424094386	0.709739666
269210548	Sphingomyelin (d18:1,C18:0) (additional: Sphingomyelin (d16:1,C20:0))	1.0213	0.7395	1.0827	0.875917842	0.025494042	0.554888957	0.987924779	0.288796737	0.882332264
69210114	DAG (C18:1,C18:2)	0.7619	1.143	1.0176	0.170361928	0.45857196	0.92392999	0.881182388	0.780967006	0.988057247
29210478	16-Methylheptadecanoic acid	1.1798	1.053	1.2073	0.079831642	0.571900396	0.045441507	0.723327965	0.846563087	0.47122359
29210485	Eicosaenoic acid (C20:1) No 02	0.987	1.1714	1.1214	0.941821623	0.368743418	0.520528409	0.987924779	0.700145731	0.866490959
29210070	Eicosaenoic acid (C20:cis[11]1)	0.8444	1.3115	1.0653	0.205063129	0.039969821	0.631618613	0.951323797	0.338224166	0.907279022
29210159	Nervonic acid (C24:cis[15]1)	1.0943	0.9676	0.9381	0.254161849	0.669295967	0.414932119	0.951497416	0.910613009	0.818944971
29210006	Oleic acid (C18:cis[9]1)	0.8185	1.1436	1.178	0.096965165	0.253167487	0.170195136	0.785507374	0.619159614	0.668073386
29210011	Arachidonic acid (C20:cis[5,8,11,14]4)	0.9844	0.8921	1.0834	0.827913746	0.111004854	0.268862633	0.987924779	0.438177056	0.720167768
29210482	dihomo-gamma-Linolenic acid (C20:cis[8,11,14]3)	1.0221	1.0273	1.0666	0.817355444	0.771464118	0.49467852	0.987924779	0.959002356	0.857880273
29210015	Docosahexaenoic acid (C22:cis[4,7,10,13,16,19]6)	1.1092	0.9497	0.9964	0.199890066	0.512533289	0.964221617	0.951323797	0.796265337	0.995804743
259210493	Docosapentaenoic acid (C22:cis[7,10,13,16,19]5)	1.1777	1.0724	1.067	0.346908952	0.680560773	0.70646223	0.963288743	0.910613009	0.946154773
29210012	Eicosapentaenoic acid (C20:cis[5,8,11,14,17]5)	1.1508	1.2605	1.183	0.438513911	0.194326262	0.35153218	0.963288743	0.559066507	0.760526352
259210004	Linoleic acid (C18:cis[9,12]2)	1.0825	1.1634	1.0903	0.800489188	0.622515931	0.781615071	0.987683097	0.876734562	0.958891859
29210005	Linolenic acid (C18:cis[9,12,15]3)	0.9051	1.2357	1.0048	0.55141557	0.198859752	0.976839962	0.963288743	0.562810619	0.995804743
29210152	Behenic acid (C22:0)	1.0908	1.0495	0.9893	0.250405765	0.513148773	0.886142057	0.951497416	0.796265337	0.969056278
29210069	Eicosanoic acid (C20:0)	1.08	1.0715	0.9759	0.380105822	0.421278784	0.77860558	0.963288743	0.743433147	0.958891859
29210058	Heneicosanoic acid (C21:0)	1.0616	1.0124	1.0882	0.455856179	0.874617251	0.289637024	0.963288743	0.980282352	0.72813777
29210068	Heptadecanoic acid (C17:0)	1.019	1.0363	1.1117	0.83578108	0.688042246	0.242431012	0.987924779	0.910613009	0.709739666
29210470	Isopalmitic acid (C16:0)	1.1148	0.9356	1.1162	0.290577966	0.507439302	0.282172732	0.963288743	0.795636537	0.720313821
29210052	Lignoceric acid (C24:0)	1.1005	1.1023	1.0579	0.222296784	0.2052806	0.468830442	0.951497416	0.568491235	0.849302258
29210105	Myristic acid (C14:0)	0.828	0.958	1.1704	0.074453991	0.675274022	0.133055691	0.723327965	0.910613009	0.623698552
29210003	Palmitic acid (C16:0)	1.0311	0.9938	1.0396	0.743108831	0.945661986	0.675455911	0.967341023	0.989177255	0.926692561
259210007	Stearic acid (C18:0)	1.1423	0.9846	1.0197	0.669903116	0.959547993	0.949867379	0.963288743	0.990359167	0.995804743
29210072	Tricosanoic acid (C23:0)	1.1116	1.0074	1.0651	0.243904103	0.933851475	0.482897996	0.951497416	0.989177255	0.852586871
29210063	Dodecanol	0.9334	1.027	0.8256	0.643274129	0.854691074	0.197657718	0.963288743	0.978653902	0.678976895
29210002	Glycerol, lipid fraction	0.835	1.2252	1.0835	0.164722249	0.111004266	0.531750688	0.881182388	0.438177056	0.870137489
29210109	Tetradecanol	0.9658	1.0869	1.0206	0.764832132	0.465440646	0.859928705	0.967341023	0.781523472	0.969056278
29210045	Galactose, lipid fraction	1.2632	0.8751	0.892	0.0055707	0.099391072	0.162672278	0.414348997	0.429133622	0.668073386
29211194	myo-Inositol, lipid fraction	1.0431	0.9947	1.0323	0.492496123	0.929296646	0.603031731	0.963288743	0.989177255	0.9003228
29210098	Rhamnose, lipid fraction (additional: Ribose, lipid fraction)	1.1001	0.785	1.249	0.599867115	0.176897426	0.221005413	0.963288743	0.537863796	0.693716712
29211385	Ribose, lipid fraction	1.0349	0.8294	1.2337	0.840690017	0.265810214	0.218591225	0.987924779	0.636247854	0.693716712
19215432	Choline phosphate	0.9573	1.0047	1.0511	0.56683074	0.946496265	0.481416408	0.963288743	0.989177255	0.852586871
39210053	Glycerol-3-phosphate, polar fraction	0.9705	1.1869	1.0135	0.769573525	0.089998424	0.894408088	0.967341023	0.424094386	0.969056278
39211739	O-Phosphoethanolamine	1.0554	1.0203	0.9559	0.628167755	0.853964979	0.683858721	0.963288743	0.978653902	0.929717294
269210094	Lysophosphatidylcholine (C16:0)	1.1643	0.6781	1.2027	0.473627934	0.064664656	0.381918835	0.963288743	0.391264324	0.788364568
69210036	Lysophosphatidylcholine (C17:0)	1.0511	0.9102	0.8872	0.600922342	0.28128988	0.178483756	0.963288743	0.651178151	0.669510821
69210096	Lysophosphatidylcholine (C18:0)	1.1862	0.9615	0.8893	0.14891985	0.713945824	0.283323436	0.881182388	0.92751463	0.720313821
69210005	Lysophosphatidylcholine (C18:1)	0.9833	0.901	1.0104	0.843592777	0.184844013	0.895838692	0.987924779	0.557497683	0.969056278
69210100	Lysophosphatidylcholine (C20:4)	1.0249	0.7199	0.7255	0.893376699	0.053707953	0.063355832	0.987924779	0.375188182	0.491553865
69210093	Lysophosphatidylethanolamine (C18:0)	1.0561	0.8763	0.8432	0.58616927	0.152582049	0.070220609	0.963288743	0.490442302	0.510504979
19210073	Isopentenyl pyrophosphate (IPP)	1.2163	0.9908	0.8965	0.033348745	0.916484379	0.226338395	0.6838209	0.989011919	0.697618339
269210129	Phosphatidylcholine (C16:0,C18:2)	0.9451	1.171	1.2352	0.670058298	0.226317404	0.112085793	0.963288743	0.581611226	0.579754101
269210135	Phosphatidylcholine (C16:0,C20:4) (additional: Phosphatidylcholine (C18:2,C18:2))	1.0515	0.9818	0.9941	0.533313496	0.816394311	0.94065292	0.963288743	0.962101508	0.993647451
269210142	Phosphatidylcholine (C16:0,C22:6) (additional: Phosphatidylcholine (C18:2,C20:4))	1.063	0.9927	0.9016	0.483466403	0.931041811	0.233253592	0.963288743	0.989177255	0.700738381
69210622	Phosphatidylcholine (C16:1,C18:2)	0.9875	1.1406	1.0771	0.941496572	0.400703524	0.639932127	0.987924779	0.730903574	0.909415222
269210136	Phosphatidylcholine (C18:0,C18:1)	0.9171	0.9497	1.356	0.486368738	0.671391966	0.01577095	0.963288743	0.910613009	0.455937798
269210628	Phosphatidylcholine (C18:0,C18:2)	0.933	1.333	0.8134	0.603310106	0.030698654	0.122604713	0.963288743	0.294839309	0.610107695
269210144	Phosphatidylcholine (C18:0,C20:4)	0.9066	0.9012	0.9925	0.329904675	0.291530036	0.940075543	0.963288743	0.651178151	0.993647451
269210155	Phosphatidylcholine (C18:0,C22:6)	1.1406	1.017	1.0125	0.060877103	0.803621811	0.857275941	0.723327965	0.960582746	0.969056278
269210014	Phosphatidylcholine (C18:1,C18:2) (additional: Phosphatidylcholine (C16:0,C20:3))	0.9912	1.0846	1.4121	0.930606508	0.414380383	0.001033101	0.987924779	0.73878449	0.154965164
269210143	Phosphatidylcholine No 02	1.1767	1.001	0.9145	0.051012257	0.98985219	0.276099539	0.723327965	0.998352634	0.720313821
259210044	Glycerol phosphate, lipid fraction	1.1371	1.1061	1.093	0.751035039	0.799206641	0.82499877	0.967341023	0.960582746	0.964284276

29210845	myo-Inositol-1-phosphate, lipid fraction (myo-Inositolphospholipids)	0.9429	1.1975	1.0712	0.82537247	0.490141961	0.795088406	0.987924779	0.78976672	0.963285466
29210148	myo-Inositol-2-phosphate, lipid fraction (myo-Inositolphospholipids)	0.8823	1.0951	0.9168	0.687274621	0.765499421	0.778481721	0.963288743	0.958269877	0.958891859
29210086	Phosphate, lipid fraction	1.0874	1.0401	0.962	0.358151189	0.658736209	0.668294997	0.963288743	0.904088827	0.925331535
269210018	Choline plasmalogen (C18,C20:4)	1.1472	1.1681	0.971	0.160203825	0.105613501	0.760622152	0.881182388	0.435526588	0.954126516
29211620	1-Hydroxy-2-amino-(cis,trans)-3,5-octadecadiene (from sphingolipids)	1.2889	1.0423	0.878	0.119380175	0.788846947	0.437068983	0.823138534	0.960582746	0.834441552
29210489	3-O-Methylsphingosine (d18:1) (additional: Sphingolipids, erythro-Sphingosine (d18:1), threo-Sphingosine (d18:1))	1.1668	1.196	0.845	0.34433342	0.262463562	0.312156007	0.963288743	0.631596807	0.732507549
29210158	4-Hydroxysphinganine (t18:0, Phytosphingosine), total	1.0137	1.0502	0.7893	0.892922931	0.620166236	0.020870108	0.987924779	0.876734562	0.466889852
29210494	5-O-Methylsphingosine (d18:1) (additional: Sphingolipids, erythro-Sphingosine (d18:1), threo-Sphingosine (d18:1))	1.107	1.1846	0.9161	0.389706382	0.14564444	0.455667871	0.963288743	0.484186328	0.849302258
29211289	erythro-Dihydrosphingosine (d18:0)	1.1425	1.0987	0.8922	0.154367243	0.302391178	0.218678644	0.881182388	0.654211684	0.693716712
29210495	erythro-Sphingosine (d18:1) (additional: Sphingolipids)	1.1047	1.1881	0.902	0.362845424	0.110186167	0.343013804	0.963288743	0.438177056	0.749017353
29211391	erythro-Sphingosine-1-phosphate (d18:1)	1.1798	1.3091	0.6977	0.527731622	0.294885699	0.168957666	0.963288743	0.651178151	0.668073386
29210491	threo-Sphingosine (d18:1) (additional: Sphingolipids)	1.137	1.1564	0.8907	0.203680052	0.142521513	0.261159029	0.951323797	0.484186328	0.713352247
269210122	Sphingomyelin (d18:1,C16:0)	1.124	1.0085	0.8334	0.436210861	0.954006509	0.223530941	0.963288743	0.989177255	0.693716712
69210140	Sphingomyelin (d18:1,C23:0)	1.0206	0.976	1.0025	0.852273321	0.808066534	0.980370614	0.987924779	0.960582746	0.995804743
69210145	Sphingomyelin (d18:1,C24:0)	1.0864	0.9987	1.1653	0.371903574	0.9880741	0.078679005	0.963288743	0.998352634	0.517737973
69210031	TAG (C16:0,C18:1,C18:2)	0.8143	1.613	1.504	0.559789557	0.140515343	0.214279529	0.963288743	0.484186328	0.693716712
69210057	TAG (C16:0,C18:1,C18:3) (additional: TAG (C16:0,C18:2,C18:2), TAG (C16:1,C18:1,C18:2))	0.8002	2.288	1.5016	0.548642848	0.017118854	0.24065533	0.963288743	0.265637387	0.709739666
69210030	TAG (C16:0,C18:2)	0.6409	1.6362	1.6262	0.164307729	0.093300765	0.102527502	0.881182388	0.424094386	0.564118143
69210058	TAG (C18:1,C18:2,C18:3) (additional: TAG (C16:0,C18:1,C20:5), TAG (C16:0,C18:2,C20:4))	0.8324	2.621	1.3207	0.69368005	0.026460619	0.520499559	0.963288743	0.288796737	0.866490959
69210032	TAG (C18:1,C18:2)	0.6438	1.7481	1.7889	0.227082976	0.095473662	0.087598646	0.951497416	0.427676573	0.525591877
39210012	Citrate	1.4126	2.0124	0.7803	0.058660651	0.000178412	0.168892564	0.723327965	0.080285625	0.668073386
39210005	Fumarate	1.3197	0.9256	1.0131	0.005376586	0.415361058	0.892379497	0.414348997	0.73878449	0.969056278
39210007	Malate	1.313	0.9241	0.9858	0.007311948	0.415070535	0.883793932	0.414348997	0.73878449	0.969056278
39210086	Succinate	1.4538	1.7455	1.2592	0.03799005	0.002008691	0.193722573	0.6838209	0.204866561	0.675776419
19215125	Carnitine	1.1968	0.8909	1.0555	0.080309234	0.216036729	0.567141482	0.723327965	0.568491235	0.882941802
19215452	O-Acetylcarnitine	0.8491	1.3289	1.124	0.08519196	0.001501041	0.183413237	0.723327965	0.204866561	0.67102404
19215175	Choline	0.9727	0.8305	1.0512	0.752695068	0.023383208	0.540487917	0.967341023	0.288796737	0.87542665
39211741	3-Phosphoglycerate (3-PGA)	1.0342	1.7994	0.8155	0.919453045	0.075115558	0.538019702	0.987924779	0.400279356	0.87542665
39210034	Fructose	0.8519	0.9303	0.938	0.385717787	0.688922155	0.726656849	0.963288743	0.910613009	0.949244757
19210411	Fructose-1,6-diphosphate	0.7031	3.6463	0.6532	0.512603706	0.016242897	0.426060422	0.963288743	0.265637387	0.829987835
39211920	Fructose-6-phosphate	1.0448	2.3983	0.5334	0.944304498	0.15816006	0.315792143	0.988225638	0.501211458	0.732507549
39210994	Glucose-6-phosphate	1.0416	2.727	0.4846	0.956320796	0.171377737	0.328644389	0.993866878	0.528219053	0.741148315
39210076	Glycerate	0.9988	1.1183	1.2456	0.994073339	0.47058995	0.164723501	0.999882889	0.785372289	0.668073386
39210085	Lactate	0.9911	0.8438	1.2096	0.933334623	0.109699448	0.078116218	0.987924779	0.438177056	0.517737973
39210002	Pyruvate (additional: Phosphoenolpyruvate (PEP))	1.1545	1.1786	0.981	0.262328889	0.191287484	0.87997838	0.951497416	0.559066507	0.969056278
39210393	3-Hydroxybutyrate	0.9448	0.72	2.1466	0.860615527	0.302017426	0.020068403	0.987924779	0.654211684	0.466889852
39210930	Ribose-5-phosphate	1.243	1.4313	1.0475	0.05823591	0.001861017	0.680158518	0.723327965	0.204866561	0.927488888
19210304	Ribulose-5-phosphate	1.1855	0.6792	0.9505	0.307465726	0.019924471	0.758331645	0.963288743	0.288796737	0.954126516
19215334	Acetylcholine	1.3592	1.1617	0.957	0.203024239	0.494808077	0.843461824	0.951323797	0.78976672	0.96811619
39210068	gamma-Aminobutyrate (GABA)	0.8783	1.2506	1.3805	0.460867418	0.196226424	0.068156462	0.963288743	0.559066507	0.510504979
19215394	Guanosine monophosphate, cyclic (cGMP)	1.0496	0.8377	0.9518	0.87513329	0.530725798	0.862926984	0.987924779	0.8173892	0.969056278
19210305	N,N-Dimethylarginine (ADMA)	0.9508	0.8899	1.0581	0.663536074	0.306396383	0.624223808	0.963288743	0.659016186	0.9003228
19215465	Serotonin (5-HT)	1.9422	1.194	1.2494	0.072893168	0.596137508	0.512189923	0.723327965	0.862578388	0.866490959
29210021	beta-Sitosterol	1.2384	0.8819	0.7263	0.084001709	0.284643944	0.008711579	0.723327965	0.651178151	0.326684201
29210053	Campesterol	1.1799	0.9421	0.8772	0.109388853	0.552157646	0.200242685	0.82236035	0.83100649	0.682645518
19215440	Ergothioneine	0.9678	1.0213	0.7699	0.805448319	0.862342242	0.037393082	0.987683097	0.980282352	0.47122359
29210857	Adenine, lipid fraction	0.6891	1.0814	1.1823	0.431255394	0.869948119	0.661263399	0.963288743	0.980282352	0.924125868
39210171	bis-Glyceryl phosphate, polar fraction	1.2178	0.9188	0.6753	0.118526033	0.489990456	0.002319968	0.823138534	0.78976672	0.20879708
29210078	Ethanolamine	1.1431	0.9993	1.0268	0.147661398	0.993411909	0.771684273	0.881182388	0.998352634	0.958891859
39210113	Glycerol-2-phosphate	1.0455	1.1314	0.9803	0.58835907	0.12820175	0.807449981	0.963288743	0.472813368	0.963285466
39210051	Phosphate (inorganic and from organic phosphates)	0.9928	0.8877	1.0291	0.90038611	0.03821895	0.616894587	0.987924779	0.336487529	0.9003228
39211737	Pyrophosphate (PPi)	0.8633	1.6708	0.6744	0.614068499	0.075383368	0.17644523	0.963288743	0.400279356	0.669510821
29210852	Serine, lipid fraction	1.0996	0.981	0.9819	0.159771078	0.7702631	0.783992718	0.881182388	0.959002356	0.958891859
29210856	Taurine, lipid fraction	1.1321	0.4423	0.3737	0.785817126	0.065210721	0.03937097	0.979550434	0.391264324	0.47122359
19215418	Trimethylamine-N-oxide (TMAO)	1.0773	1.9069	0.7342	0.757593485	0.004638377	0.170729865	0.967341023	0.234208529	0.668073386
39210011	Putrescine (additional: Agmatine)	1.1216	0.9616	0.7362	0.458989795	0.796087267	0.049792785	0.963288743	0.960582746	0.47122359
19210205	Adenosine diphosphate (ADP) (additional: Adenosine triphosphate (ATP))	1.0295	2.9372	0.9396	0.93317308	0.009715319	0.86497009	0.987924779	0.234208529	0.969056278
39211750	Adenosine monophosphate (AMP)	0.9247	1.2505	0.9094	0.553594124	0.087264918	0.46994725	0.963288743	0.424094386	0.849302258
19215445	1-Methyladenosine	1.1199	0.7488	1.0991	0.258573827	0.002276295	0.309469874	0.951497416	0.204866561	0.732507549
19215448	7-Methylguanosine	1.0822	0.9457	0.9299	0.567756592	0.658980301	0.571291602	0.963288743	0.904088827	0.88321389
39211752	Adenine	0.9741	1.0655	0.9376	0.698473441	0.339488599	0.339557648	0.963288743	0.692628146	0.749017353
39210177	Adenosine	1.4107	1.0317	1.7218	0.286601721	0.921092209	0.092589695	0.962468465	0.989177255	0.541108605
19215461	Allantoin	1.0377	0.9751	1.0775	0.766571447	0.824951265	0.519391867	0.967341023	0.962192848	0.866490959
39211925	Guanosine	1.3703	0.7761	0.9309	0.069954529	0.134975192	0.674775613	0.723327965	0.478258554	0.926692561
39210619	Hypoxanthine (additional: Inosine)	0.9991	0.727	0.7809	0.994543825	0.013293622	0.055971363	0.999882889	0.256725822	0.47122359
39211762	Inosine	1.4363	0.658	0.8068	0.083408802	0.04190578	0.297851218	0.723327965	0.338224166	0.732507549
19210301	Uric acid	0.8715	0.625	0.7554	0.732018462	0.234373838	0.482733994	0.967341023	0.59586569	0.852586871
39210625	Xanthine	1.2282	0.7267	1.2546	0.254321862	0.072898784	0.206237764	0.951497416	0.400279356	0.693716712
19215415	2'-Deoxycytidine	1.0712	0.8081	0.991	0.507445634	0.027622576	0.924875122	0.963288743	0.288796737	0.988057247
19215469	2'-Deoxythymidine	1.2881	0.8769	0.8051	0.044309294	0.24889957	0.062977342	0.70111745	0.615411024	0.491553865
19215443	5-Methylcytidine	1.3592	0.9464	1.0564	0.022113663	0.646638795	0.65306838	0.6838209	0.895346023	0.91933877
39210441	Cytosine (additional: 2'-Deoxycytidine)	0.9434	1.012	0.8327	0.480236902	0.88554697	0.028373602	0.963288743	0.980282352	0.47122359
39211809	Pseudouridine	1.0427	1.1803	0.9478	0.674244236	0.092427272	0.587725212	0.963288743	0.424094386	0.891769314
19210407	Uridine	1.1904	0.9268	1.0194	0.109648047	0.472268657	0.857484337	0.82236035	0.785372289	0.969056278
269210047	Unknown lipid (269210047)	0.9676	1.0813	1.0627	0.749144684	0.439041419	0.552983654	0.967341023	0.759879378	0.882332264
269210085	Unknown lipid (269210085)	0.9188	1.4553	1.0541	0.7551622	0.161204985	0.845016011	0.967341023	0.503765578	0.96811619
269210127	Unknown lipid (269210127)	0.8614	0.9145	1.2851	0.243545065	0.474405731	0.050831734	0.951497416	0.785372289	0.47122359
269210146	Unknown lipid (269210146)	1.0188	1.0242	1.0674	0.921910922	0.897930404	0.730648355	0.987924779	0.980876989	0.949244757
269210154	Unknown lipid (269210154)	1.0854	0.896	1.1255	0.485922576	0.341142061	0.312904467	0.963288743	0.692628146	0.732507549
269210291	Unknown lipid (269210291)	1.2477	1.2533	0.5922	0.554891902	0.538573352	0.162238545	0.963288743	0.8173892	0.668073386
269210531	Unknown lipid (269210531)	1.2128	0.804	0.7339	0.219178824	0.156909273	0.049507593	0.951497416	0.500774275	0.47122359
269210561	Unknown lipid (269210561)	1.0748	1.0053	1.0368	0.309986111	0.939275881	0.60797775	0.963288743	0.989177255	0.9003228
269210620	Unknown lipid (269210620)	0.9107	0.9717	1.2339	0.596798385	0.868374686	0.23357946	0.963288743	0.980282352	0.700738381
269210624	Unknown lipid (269210624)	1.1019	0.9943	0.9564	0.330451569	0.952872629	0.651591305	0.963288743	0.989177255	0.91933877
269210625	Unknown lipid (269210625)	1.0431	0.899	1.3284	0.654273758	0.250523845	0.003355784	0.963288743	0.616042243	0.217550827
269210629	Unknown lipid (269210629)	1.1093	1.1122	1.0544	0.33403057	0.311827271	0.618384912	0.963288743	0.659016186	0.9003228
269210636	Unknown lipid (269210636)	1.1382	0.991	0.9632	0.140399555	0.915898629	0.665282615	0.881182388	0.989011919	0.925331535
269210642	Unknown lipid (269210642)	1.0917	0.8388	1.0147	0.419425021	0.101084809	0.892500677	0.963288743	0.429133622	0.969056278
269210802	Unknown lipid (269210802)	1.0102	0.9534	0.9132	0.897444897	0.53824028	0.249812346	0.987924779	0.8173892	0.712770869
269210805	Unknown lipid (269210805)	1.1194	1.1208	1.0433	0.369935553	0.354933373	0.734082612	0.963288743	0.694282674	0.949244757
269210818	Unknown lipid (269210818)	0.9131	0.7646	1.2531	0.537589317	0.066141501	0.126519073	0.963288743	0.391627306	0.612189063
269210819	Unknown lipid (269210819)	1.1397	0.8676	1.0585	0.154828853	0.115114659	0.531284847	0.881182388	0.449513079	0.870137489
269210820	Unknown lipid (269210820)	1.1861	0.9853	1.268	0.297162958	0.92612964	0.145951538	0.963288743	0.989177255	0.656781922
269210895	Unknown lipid (269210895)	1.0186	1.0264	0.8231	0.857977779	0.795581243	0.060179838	0.987924779	0.960582746	0.491553865
269210898	Unknown lipid (269210898)	1.1663	1.0303	0.9349	0.267501532	0.825347643	0.624180737	0.951497416	0.962192848	0.9003228
269210899	Unknown lipid (269210899)	0.9481	0.6754	1.1005	0.707271233	0.006099733	0.497658701	0.963288743	0.234208529	0.858032243
269210900	Unknown lipid (269210900)	1.0579	1.0915	0.9163	0.519614451	0.307742619	0.315563243	0.963288743	0.659016186	0.732507549
269210902	Unknown lipid (269210902)	0.9822	0.963	0.9025	0.857375859	0.699647473	0.302406126	0.987924779	0.915236521	0.732507549
269210910	Unknown lipid (269210910)	1.1483	0.9607	1.0267	0.069113685	0.58581822	0.724396943	0.723327965	0.854501069	0.949244757
29210077	Unknown lipid (29210077)	1.0464	0.9991	0.9606	0.33931155	0.984927091	0.393760611	0.963288743	0.998352634	0.805419432
29210093	Unknown lipid (29210093)	0.9039	0.947	0.9974	0.292981826	0.561461518	0.978117051	0.963288743	0.841403102	0.995804743
29210099	Unknown lipid (29210099)	1.0885	1.0195	0.9672	0.606620393	0.904586557	0.838236299	0.963288743	0.980876989	0.96811619
29210332	Unknown lipid (29210332)	1.1839	1.1233	1.1442	0.235642672	0.402809081	0.340205009	0.951497416	0.730903574	0.749017353
29210676	Unknown lipid (29210676)	1.099	0.9528	0.8667	0.118364112	0.410351589	0.018613337	0.823138534	0.73878449	0.465333418
29210686	Unknown lipid (29210686)	1.1876	0.9616	1.0716	0.03189988	0.611469338	0.377930419	0.6838209	0.873527625	0.783726676
29210969	Unknown lipid (29210969)	0.8797	0.9369	1.0096	0.350878306	0.627352286	0.944211937	0.963288743	0.876734562	0.995071128
29211283	Unknown lipid (29211283)	1.0577	0.8154	0.7714	0.788827422	0.351516741	0.212749939	0.980586574	0.693783042	0.693716712
29211296	Unknown lipid (29211296)	1.1022	0.9333	1.0005	0.352502442	0.499864709	0.995858666	0.963288743	0.791598258	0.998080593
29211312	Unknown lipid (29211312)	0.9165	1.1032	1.0651	0.365993041	0.298927612	0.51003454	0.963288743	0.654211684	0.866490959
29211361	Unknown lipid (29211361)	1.0778	0.9991	1.018	0.549004592	0.993811638	0.885405958	0.963288743	0.998352634	0.969056278
29211367	Unknown lipid (29211367)	0.94	1.1055	1.0067	0.738219654	0.580020232	0.971082142	0.967341023	0.850192523	0.995804743
29211389	Unknown lipid (29211389)	1.1007	1.0057	1.26	0.388022392	0.958031421	0.038993861	0.963288743	0.990359167	0.47122359
29211443	Unknown lipid (29211443)	1.1238	0.942	0.9097	0.242172284	0.539476872	0.33881455	0.951497416	0.8173892	0.749017353
29211617	Unknown lipid (29211617)	1.1018	0.9739	0.9751	0.376025348	0.804781212	0.816007835	0.963288743	0.960582746	0.963542296
29211621	Unknown lipid (29211621)	0.8997	1.098	1.0561	0.2640686	0.31271371	0.560319518	0.951497416	0.659016186	0.882941802
69210017	Unknown lipid (69210017)	1.0109	1.0579	1.2461	0.92182923	0.578148674	0.035343992	0.987924779	0.850192523	0.47122359
69210047	Unknown lipid (69210047)	0.9591	0.8805	1.0637	0.608789973	0.090808389	0.414866422	0.963288743	0.424094386	0.818944971
69210061	Unknown lipid (69210061)	0.9653	0.9183	0.9994	0.767758717	0.436530908	0.995862636	0.967341023	0.759201111	0.998080593
69210085	Unknown lipid (69210085)	1.1551	1.165	1.1556	0.406631551	0.336651229	0.370278629	0.963288743	0.692628146	0.778587307
69210104	Unknown lipid (69210104)	0.9552	1.0619	1.213	0.68747719	0.564550355	0.071643571	0.963288743	0.841403102	0.510504979
69210121	Unknown lipid (69210121)	1.0474	0.931	1.0027	0.671938458	0.474713917	0.978965303	0.963288743	0.785372289	0.995804743
69210123	Unknown lipid (69210123)	0.8713	1.2964	0.8866	0.456415382	0.127436209	0.48313256	0.963288743	0.472813368	0.852586871
69210141	Unknown lipid (69210141)	0.9927	1.0061	0.8312	0.948767589	0.9535735	0.085450284	0.990592611	0.989177255	0.519630104
69210149	Unknown lipid (69210149)	1.0928	0.8789	0.8158	0.565823003	0.36203948	0.159025455	0.963288743	0.694282674	0.668073386
69210153	Unknown lipid (69210153)	1.1375	0.9786	1.0823	0.410655315	0.879789203	0.586087947	0.963288743	0.980282352	0.891769314
69210157	Unknown lipid (69210157)	0.9853	1.0623	0.9095	0.935740319	0.719089273	0.578782062	0.987924779	0.92751463	0.88891443
69210158	Unknown lipid (69210158)	1.0862	0.9593	0.9198	0.641538382	0.797789958	0.612070773	0.963288743	0.960582746	0.9003228
69210187	Unknown lipid (69210187)	0.6891	1.3755	1.5475	0.124385378	0.149533413	0.053597677	0.823138534	0.484186328	0.47122359
69210233	Unknown lipid (69210233)	1.4925	2.6506	0.6982	0.278773927	0.005012717	0.295087521	0.957338027	0.234208529	0.732507549

69210234	Unknown lipid (69210234)	1.335	1.9578	0.685	0.435311593	0.050116511	0.272269464	0.963288743	0.375188182	0.720313821
69210236	Unknown lipid (69210236)	1.2589	1.0286	0.7699	0.278523592	0.883884824	0.18611972	0.957338027	0.980282352	0.674558256
69210293	Unknown lipid (69210293)	1.4134	1.8413	0.8093	0.247124742	0.027641571	0.444451177	0.951497416	0.288796737	0.834441552
69210321	Unknown lipid (69210321)	0.9525	1.0584	1.0181	0.638617114	0.549086714	0.851959953	0.963288743	0.829157789	0.969056278
69210327	Unknown lipid (69210327)	0.9351	1.0966	1.2175	0.533780845	0.350764847	0.052621712	0.963288743	0.693783042	0.47122359
69210442	Unknown lipid (69210442)	0.9957	0.8508	0.9933	0.97152302	0.143554015	0.951751154	0.999882889	0.484186328	0.995804743
69210457	Unknown lipid (69210457)	1.0331	1.1446	0.9859	0.863589537	0.436962417	0.935643379	0.987924779	0.759201111	0.993017738
69210505	Unknown lipid (69210505)	1.149	0.8301	1.0545	0.449072349	0.267968976	0.754980393	0.963288743	0.63802137	0.954126516
69210507	Unknown lipid (69210507)	1.0195	0.6877	0.789	0.902469312	0.011450195	0.109428846	0.987924779	0.234208529	0.572592799
69210510	Unknown lipid (69210510)	1.1037	1.1962	0.9488	0.380287214	0.084111146	0.613761317	0.963288743	0.422989828	0.9003228
69210512	Unknown lipid (69210512)	1.0583	0.9259	0.6804	0.678499289	0.538283508	0.003384124	0.963288743	0.8173892	0.217550827
69210519	Unknown lipid (69210519)	1.3946	0.7318	0.6458	0.101614575	0.092827094	0.021788193	0.788388942	0.424094386	0.466889852
69210527	Unknown lipid (69210527)	0.8497	1.0435	1.2831	0.282946572	0.757540603	0.078987718	0.957338027	0.957565369	0.517737973
69210558	Unknown lipid (69210558)	1.0801	0.7836	0.8425	0.466394422	0.013692044	0.084134478	0.963288743	0.256725822	0.519630104
69210564	Unknown lipid (69210564)	1.0005	1.2289	0.8144	0.998204078	0.272843857	0.28229142	0.999882889	0.641212968	0.720313821
69210568	Unknown lipid (69210568)	1.1206	1.5007	0.8888	0.584726795	0.036127994	0.542682409	0.963288743	0.325151942	0.87542665
69210576	Unknown lipid (69210576)	0.9253	1.0365	0.7956	0.598662982	0.790248756	0.09844346	0.963288743	0.960582746	0.560753886
69210579	Unknown lipid (69210579)	1.088	0.8062	0.9031	0.704797642	0.291602659	0.621986872	0.963288743	0.651178151	0.9003228
69210597	Unknown lipid (69210597)	1.0394	1.0143	1.1254	0.762847683	0.903552165	0.321768574	0.967341023	0.980876989	0.735140572
69210603	Unknown lipid (69210603)	0.5767	1.6357	1.4961	0.081465657	0.088218811	0.167658112	0.723327965	0.424094386	0.668073386
69210605	Unknown lipid (69210605)	0.6388	1.829	1.3708	0.192608251	0.056695639	0.321828206	0.951323797	0.375188182	0.735140572
69210607	Unknown lipid (69210607)	0.9	1.2967	1.0443	0.586358339	0.145150695	0.809159792	0.963288743	0.484186328	0.963285466
69210608	Unknown lipid (69210608)	0.5982	1.7355	1.2647	0.122722372	0.071016583	0.4440731	0.823138534	0.400279356	0.834441552
69210610	Unknown lipid (69210610)	0.9798	0.9367	1.0121	0.833195904	0.459902792	0.892967061	0.987924779	0.780967006	0.969056278
69210611	Unknown lipid (69210611)	0.8745	0.8249	1.3592	0.422384315	0.209338293	0.050879655	0.963288743	0.568491235	0.47122359
69210625	Unknown lipid (69210625)	0.8832	0.9858	1.0964	0.177842661	0.864566981	0.281184375	0.899204465	0.980282352	0.720313821
69210658	Unknown lipid (69210658)	1.0415	0.9742	1.053	0.694739015	0.782417858	0.591777491	0.963288743	0.959389312	0.893623728
69210803	Unknown lipid (69210803)	1.2299	1.0357	0.8694	0.153628982	0.789935412	0.296850726	0.881182388	0.960582746	0.732507549
69210806	Unknown lipid (69210806)	0.9143	0.9987	1.2259	0.535258667	0.992268175	0.131819436	0.963288743	0.998352634	0.623698552
69210813	Unknown lipid (69210813)	0.9604	1.2254	1.1636	0.729456263	0.06035734	0.165353964	0.967341023	0.382546521	0.668073386
69210814	Unknown lipid (69210814)	1.0735	0.8211	0.7983	0.662346192	0.187071445	0.138418689	0.963288743	0.557497683	0.629175857
69210822	Unknown lipid (69210822)	1.0775	0.8025	1.0634	0.54223462	0.052658638	0.588567748	0.963288743	0.375188182	0.891769314
69210825	Unknown lipid (69210825)	1.1102	1.0786	1.0125	0.399712833	0.504560358	0.914246616	0.963288743	0.793888676	0.981887774
69210826	Unknown lipid (69210826)	1.088	0.9105	1.1128	0.487996026	0.399616032	0.344547983	0.963288743	0.730903574	0.749017353
69210858	Unknown lipid (69210858)	0.9098	1.1207	0.9307	0.486510116	0.359715208	0.569006939	0.963288743	0.694282674	0.882941802
69210868	Unknown lipid (69210868)	0.9795	1.1111	1.1232	0.812521055	0.188938697	0.154545848	0.987924779	0.559066507	0.668073386
69210874	Unknown lipid (69210874)	1.0865	0.9732	1.083	0.281286855	0.698501128	0.265264886	0.957338027	0.915236521	0.719091559
69210875	Unknown lipid (69210875)	1.2141	1.4605	1.3008	0.583746545	0.243705147	0.424234322	0.963288743	0.609262867	0.829987835
69210876	Unknown lipid (69210876)	1.5289	2.0256	0.7699	0.143399698	0.009021384	0.329399251	0.881182388	0.234208529	0.741148315
69210877	Unknown lipid (69210877)	0.9196	0.8067	1.0848	0.705565812	0.291588036	0.692733594	0.963288743	0.651178151	0.933323704
69210878	Unknown lipid (69210878)	1.0775	1.8257	0.907	0.804119872	0.031654442	0.726719127	0.987683097	0.296760392	0.949244757
69210879	Unknown lipid (69210879)	0.9676	1.2171	1.0761	0.909143193	0.45694032	0.784160454	0.987924779	0.780967006	0.958891859
69210880	Unknown lipid (69210880)	1.0985	1.9517	0.6978	0.783654672	0.035612738	0.259468127	0.979550434	0.325151942	0.713352247
69210881	Unknown lipid (69210881)	1.1262	1.5685	0.8788	0.732596475	0.159878244	0.688980758	0.967341023	0.503113355	0.931402095
69210883	Unknown lipid (69210883)	0.7921	1.2	0.8818	0.2127734	0.285602055	0.467110083	0.951497416	0.651178151	0.849302258
69210884	Unknown lipid (69210884)	1.1469	1.6533	1.2479	0.675150567	0.095989631	0.466361706	0.963288743	0.427676573	0.849302258
69210885	Unknown lipid (69210885)	1.4357	1.8767	1.1308	0.344845457	0.074657857	0.728517836	0.963288743	0.400279356	0.949244757
69210886	Unknown lipid (69210886)	1.3984	2.1299	1.2136	0.446222984	0.062994606	0.635095315	0.963288743	0.390396799	0.907279022
69210887	Unknown lipid (69210887)	1.2431	1.2696	1.6859	0.48014303	0.397138553	0.071025853	0.963288743	0.730903574	0.510504979
69210888	Unknown lipid (69210888)	1.1815	0.7589	0.731	0.450877071	0.174627751	0.129645839	0.963288743	0.534574748	0.620644976
69210889	Unknown lipid (69210889)	0.9633	1.0559	1.16	0.644833039	0.464070837	0.052302633	0.963288743	0.781523472	0.47122359
69210890	Unknown lipid (69210890)	0.7277	0.9676	1.0198	0.217716288	0.8882995	0.934473972	0.951497416	0.980282352	0.993017738
69210891	Unknown lipid (69210891)	0.8346	1.2665	1.0472	0.06672002	0.009709699	0.610286981	0.723327965	0.234208529	0.9003228
69210892	Unknown lipid (69210892)	0.6025	2.4843	1.4566	0.265090406	0.030794328	0.371991713	0.951497416	0.294839309	0.778587307
69210893	Unknown lipid (69210893)	0.7455	2.4257	1.5177	0.486668993	0.024236297	0.288497772	0.963288743	0.288796737	0.72813777
69210894	Unknown lipid (69210894)	1.0949	0.8861	0.9546	0.43609723	0.256780429	0.666248167	0.963288743	0.624601044	0.925331535
69210895	Unknown lipid (69210895)	1.0042	0.9115	1.0312	0.964851743	0.292235995	0.730147891	0.998122493	0.651178151	0.949244757
69210896	Unknown lipid (69210896)	0.9209	1.0345	1.2571	0.587846469	0.806971701	0.108365612	0.963288743	0.960582746	0.572592799
69210897	Unknown lipid (69210897)	0.9623	1.0495	0.9674	0.713753528	0.613871219	0.732895087	0.967341023	0.874183698	0.949244757
69210898	Unknown lipid (69210898)	1.1193	0.9086	0.9708	0.294576793	0.329300194	0.765421494	0.963288743	0.682880586	0.954126516
69210899	Unknown lipid (69210899)	1.0145	1.0123	1.0646	0.890722724	0.899021101	0.521820111	0.987924779	0.980876989	0.866490959
69210900	Unknown lipid (69210900)	1.0345	1.0227	0.989	0.703035727	0.782435283	0.893843794	0.963288743	0.959389312	0.969056278
69210901	Unknown lipid (69210901)	1.0233	1.304	1.0244	0.85904534	0.028237903	0.84140697	0.987924779	0.288796737	0.96811619
69210902	Unknown lipid (69210902)	0.9722	0.9817	1.0211	0.761675257	0.828430196	0.808666899	0.967341023	0.963290926	0.963285466
69210903	Unknown lipid (69210903)	1.091	0.7904	1.1782	0.665118049	0.214582214	0.418085201	0.963288743	0.568491235	0.821564805
69210904	Unknown lipid (69210904)	0.8638	1.1166	0.9981	0.117119709	0.195294348	0.981878739	0.823138534	0.559066507	0.995804743
69210906	Unknown lipid (69210906)	0.9556	0.9099	1.1066	0.567580591	0.196294462	0.172678401	0.963288743	0.559066507	0.669476626
69210907	Unknown lipid (69210907)	0.9812	1.119	0.9406	0.886705866	0.356766534	0.620259166	0.987924779	0.694282674	0.9003228
69210908	Unknown lipid (69210908)	1.1623	0.9742	0.9053	0.245134645	0.82417366	0.406127717	0.951497416	0.962192848	0.812255435
69210909	Unknown lipid (69210909)	1.0161	1.0938	0.8498	0.925281981	0.56467497	0.304947172	0.987924779	0.841403102	0.732507549
69210910	Unknown lipid (69210910)	1.0986	0.9421	0.9183	0.034858058	0.139389531	0.039238099	0.6838209	0.484186328	0.47122359
69210911	Unknown lipid (69210911)	1.0064	0.8203	0.8697	0.95355793	0.051890545	0.174063923	0.99328951	0.375188182	0.669476626
69210912	Unknown lipid (69210912)	0.9604	1.0574	0.8851	0.706552731	0.569042641	0.222789622	0.963288743	0.845112833	0.693716712
69210913	Unknown lipid (69210913)	0.782	2.8499	1.2812	0.575005682	0.010834138	0.542764523	0.963288743	0.234208529	0.87542665
69210914	Unknown lipid (69210914)	0.7802	2.5372	1.3973	0.526582692	0.011211128	0.358666281	0.963288743	0.234208529	0.772247974
69210915	Unknown lipid (69210915)	1.0383	1.0937	0.781	0.841650589	0.602274088	0.159805985	0.987924779	0.863240341	0.668073386
69210916	Unknown lipid (69210916)	0.801	2.1179	1.0978	0.576843606	0.042090118	0.800239527	0.963288743	0.338224166	0.963285466
39210274	Unknown polar (39210274)	1.2753	0.8937	1.0364	0.013941446	0.237142868	0.709720538	0.570331883	0.599106266	0.947698048
39210389	Unknown polar (39210389)	0.8774	1.0644	0.9482	0.372480088	0.663024802	0.714221691	0.963288743	0.906872829	0.949244757
39210434	Unknown polar (39210434)	1.0093	0.9735	0.9433	0.893794132	0.694149113	0.399944754	0.987924779	0.913354096	0.807063406
39210618	Unknown polar (39210618)	0.8769	1.0987	1.0809	0.339885516	0.484215369	0.568660046	0.963288743	0.78976672	0.882941802
39211049	Unknown polar (39211049)	1.1934	0.908	0.85	0.045183125	0.259226159	0.063319381	0.70111745	0.627160063	0.491553865
39211139	Unknown polar (39211139)	1.0306	0.9956	0.8774	0.696193786	0.953488535	0.091818531	0.963288743	0.989177255	0.541108605
39211915	Unknown polar (39211915)	1.0485	0.993	0.9864	0.554193712	0.928811804	0.863366687	0.963288743	0.989177255	0.969056278
39211995	Unknown polar (39211995)	1.0904	1.4167	0.93	0.635292397	0.054628368	0.68923755	0.963288743	0.375188182	0.931402095
39212022	Unknown polar (39212022)	1.6253	0.9361	0.7153	0.010693743	0.716384355	0.072605153	0.481218454	0.92751463	0.510504979
39212289	Unknown polar (39212289)	1.1237	1.1506	1.0678	0.258831951	0.166403764	0.521322474	0.951497416	0.516425474	0.866490959
39212316	Unknown polar (39212316)	1.0032	0.974	1.1521	0.978168717	0.815673743	0.220030982	0.999882889	0.962101508	0.693716712
39212322	Unknown polar (39212322)	1.0056	0.9285	1.0706	0.935209247	0.2735842	0.320718042	0.987924779	0.641212968	0.735140572
39212325	Unknown polar (39212325)	1.0499	1.1851	0.9143	0.586932845	0.056062349	0.315160676	0.963288743	0.375188182	0.732507549
59210041	Unknown polar (59210041)	1.2333	0.9024	1.1499	0.091219377	0.393546099	0.254261749	0.760161478	0.730903574	0.713352247
59210179	Unknown polar (59210179)	1.0686	0.8044	0.9287	0.418533242	0.008242187	0.364499809	0.963288743	0.234208529	0.773702424
59210197	Unknown polar (59210197)	0.8361	0.8476	3.0313	0.750450254	0.764073767	0.05069785	0.967341023	0.958269877	0.47122359
59210252	Unknown polar (59210252)	1.1535	1.1963	0.855	0.598579251	0.500003381	0.561004453	0.963288743	0.791598258	0.882941802
59210378	Unknown polar (59210378)	1.2947	1.0315	1.0927	0.004519404	0.719339124	0.313223734	0.414348997	0.92751463	0.732507549
59210384	Unknown polar (59210384)	1.0325	0.9681	1.1733	0.694641035	0.684051285	0.051491668	0.963288743	0.910613009	0.47122359
59210813	Unknown polar (59210813)	1.2203	1.7671	1.1982	0.403666563	0.016841791	0.445035494	0.963288743	0.265637387	0.834441552
59210829	Unknown polar (59210829)	1.1006	0.9833	0.9198	0.416876991	0.886468504	0.475669008	0.963288743	0.980282352	0.852586871
59210835	Unknown polar (59210835)	1.0525	1.0233	1.1124	0.625017403	0.8222735	0.307799617	0.963288743	0.962192848	0.732507549
59210838	Unknown polar (59210838)	1.2499	0.9414	0.8022	0.176581456	0.706805036	0.179011388	0.899204465	0.921919612	0.669510821
59210845	Unknown polar (59210845)	1.0492	1.4701	0.9221	0.846420991	0.115874483	0.741987095	0.987924779	0.449513079	0.953983407
59210849	Unknown polar (59210849)	1.1885	0.6075	1.6779	0.607750711	0.133430814	0.124733129	0.963288743	0.47818165	0.610107695
59210937	Unknown polar (59210937)	0.9035	0.7342	1.1536	0.622725873	0.129235654	0.486238882	0.963288743	0.472813368	0.854716785
59210938	Unknown polar (59210938)	0.9304	0.7438	1.8263	0.771838915	0.227028092	0.017224317	0.967486105	0.581611226	0.455937798
59210939	Unknown polar (59210939)	1.2735	1.0285	0.8326	0.208155979	0.880339908	0.335853891	0.951497416	0.980282352	0.749017353
59210940	Unknown polar (59210940)	1.0999	1.1567	1.0613	0.622032108	0.441920943	0.756266464	0.963288743	0.76193266	0.954126516
59210942	Unknown polar (59210942)	0.9205	0.8161	1.0266	0.617515846	0.213306465	0.873172413	0.963288743	0.568491235	0.969056278
59210943	Unknown polar (59210943)	1.0009	1.0382	0.772	0.995471689	0.81671728	0.118423724	0.999882889	0.962101508	0.60557586
59210944	Unknown polar (59210944)	1.0408	1.0338	1.1412	0.622520439	0.676018924	0.104857144	0.963288743	0.910613009	0.568502587
59210945	Unknown polar (59210945)	0.9682	0.8397	0.986	0.74960781	0.081677659	0.888852649	0.967341023	0.417669845	0.969056278
59210946	Unknown polar (59210946)	1.0463	1.0153	1.05	0.81050142	0.93435666	0.794570076	0.987924779	0.989177255	0.963285466
59210947	Unknown polar (59210947)	1.2289	1.2075	1.0785	0.348091821	0.380565404	0.728430661	0.963288743	0.719556436	0.949244757
59210949	Unknown polar (59210949)	1.0688	0.8728	0.8777	0.662124951	0.362569841	0.389409739	0.963288743	0.694282674	0.800156998
59210950	Unknown polar (59210950)	1.1304	0.8746	1.0278	0.366153304	0.313398808	0.838348726	0.963288743	0.659016186	0.96811619
59210951	Unknown polar (59210951)	0.988	0.9134	2.9121	0.981710987	0.861013162	0.045094953	0.999882889	0.980282352	0.47122359
59210952	Unknown polar (59210952)	1.3818	0.8402	0.8999	0.000765946	0.056243181	0.250261772	0.344675883	0.375188182	0.712770869
59210953	Unknown polar (59210953)	1.3211	0.8814	0.9531	0.04253294	0.341696552	0.720698844	0.70111745	0.692628146	0.949244757
59210954	Unknown polar (59210954)	0.9816	0.778	1.397	0.905098457	0.10422811	0.034300311	0.987924779	0.434283794	0.47122359
59210955	Unknown polar (59210955)	0.9705	0.7967	2.2793	0.935887834	0.53326243	0.028799607	0.987924779	0.8173892	0.47122359
59210958	Unknown polar (59210958)	1.2048	0.9341	0.9068	0.367818765	0.735670054	0.633444421	0.963288743	0.940495101	0.907279022
59210961	Unknown polar (59210961)	1.0936	0.9956	1.2401	0.592090101	0.978236364	0.196972663	0.963288743	0.998352634	0.678976895
59210963	Unknown polar (59210963)	1.6256	0.9019	0.7781	0.002251365	0.492018451	0.102794862	0.414348997	0.78976672	0.564118143
59210964	Unknown polar (59210964)	1.3429	1.3632	1.2009	0.248158187	0.215587682	0.469171963	0.951497416	0.568491235	0.849302258
59215051	Unknown polar (59215051)	0.8833	1.1403	0.719	0.674199944	0.627115307	0.231228602	0.963288743	0.876734562	0.700738381
59215387	Unknown polar (59215387)	0.8807	1.2662	0.826	0.413931665	0.099894717	0.187377293	0.963288743	0.429133622	0.674558256
59215394	Unknown polar (59215394)	0.9238	0.8622	0.893	0.422450974	0.10375294	0.218855082	0.963288743	0.434283794	0.693716712
59215415	Unknown polar (59215415)	0.9042	1.3012	0.8912	0.730138706	0.32620045	0.671147631	0.967341023	0.67958427	0.926430779
59215478	Unknown polar (59215478)	0.9139	0.7633	1.0447	0.547967342	0.052149303	0.753105535	0.963288743	0.375188182	0.954126516
59215483	Unknown polar (59215483)	1.051	0.9538	0.9802	0.753320026	0.74414476	0.892028835	0.967341023	0.948626464	0.969056278

59215489	Unknown polar (59215489)	1.0022	0.841	0.9394	0.983553007	0.080866114	0.530010878	0.999882889	0.417669845	0.870137489
59215490	Unknown polar (59215490)	1.0632	0.9294	0.8787	0.372379665	0.245422594	0.045508042	0.963288743	0.610166671	0.47122359
59215500	Unknown polar (59215500)	1.0383	0.9596	0.9231	0.72997643	0.679238496	0.458419449	0.967341023	0.910613009	0.849302258
59215502	Unknown polar (59215502)	0.9802	0.8759	0.9988	0.889867768	0.317769529	0.993139839	0.987924779	0.665099015	0.998080593
59215503	Unknown polar (59215503)	1.2028	1.0252	0.9392	0.064362752	0.782248941	0.493490605	0.723327965	0.959389312	0.857880273
59215507	Unknown polar (59215507)	0.8722	1.2003	1.32	0.518979793	0.347556338	0.161091096	0.963288743	0.693783042	0.668073386
59215508	Unknown polar (59215508)	0.8307	1.5815	1.5797	0.400467055	0.025643139	0.028169948	0.963288743	0.288796737	0.47122359
59215510	Unknown polar (59215510)	1.2245	1.1183	0.7746	0.388913861	0.602349927	0.24324149	0.963288743	0.863240341	0.709739666
59215511	Unknown polar (59215511)	1.0365	1.0415	1.2077	0.920784941	0.901869938	0.57310768	0.987924779	0.980876989	0.88321389
59215514	Unknown polar (59215514)	1.3563	1.2119	0.7028	0.025018006	0.11855027	0.00571874	0.6838209	0.452098487	0.321679117
59215515	Unknown polar (59215515)	0.8653	1.0389	1.0143	0.519626111	0.852505958	0.946875626	0.963288743	0.978653902	0.995546803
59215516	Unknown polar (59215516)	1.6187	1.8844	0.9934	0.128354984	0.030368213	0.981770123	0.837097723	0.294839309	0.995804743
59215525	Unknown polar (59215525)	1.1061	0.7054	0.6244	0.589554578	0.044583059	0.008305745	0.963288743	0.345903048	0.326684201
59215539	Unknown polar (59215539)	0.9637	0.9953	1.2083	0.614801345	0.944139364	0.006998024	0.963288743	0.989177255	0.326684201
59215540	Unknown polar (59215540)	1.1279	1.041	0.903	0.216166178	0.650098448	0.25835215	0.951497416	0.897375158	0.713352247
59215595	Unknown polar (59215595)	0.8265	0.872	0.9303	0.122455057	0.223232036	0.52490256	0.823138534	0.580661365	0.86840497
59215596	Unknown polar (59215596)	0.9824	1.1172	1.1562	0.901138623	0.397708185	0.305847156	0.987924779	0.730903574	0.732507549
59215597	Unknown polar (59215597)	0.9365	0.8996	0.9809	0.471153671	0.206166476	0.819100915	0.963288743	0.568491235	0.963542296
59215598	Unknown polar (59215598)	1.0138	0.9815	1.0003	0.639132005	0.484420372	0.990879365	0.963288743	0.78976672	0.998080593
59215600	Unknown polar (59215600)	0.9607	0.9417	1.2355	0.667125469	0.481782446	0.016927446	0.963288743	0.78976672	0.455937798
59215602	Unknown polar (59215602)	1.1796	1.118	1.1437	0.030068522	0.106462055	0.056546831	0.6838209	0.435526588	0.47122359
59215603	Unknown polar (59215603)	1.1522	0.9102	0.7615	0.189268603	0.338663852	0.007810384	0.946343016	0.692628146	0.326684201
59215604	Unknown polar (59215604)	1.2164	0.9456	0.9156	0.021547309	0.464938309	0.257508304	0.6838209	0.781523472	0.713352247
59215605	Unknown polar (59215605)	1.1534	1.0281	0.8285	0.131480403	0.746336742	0.033785944	0.845231162	0.948733147	0.47122359
59215606	Unknown polar (59215606)	1.1702	1.0511	0.9417	0.060949346	0.510502466	0.435391044	0.723327965	0.796265337	0.834441552
59215607	Unknown polar (59215607)	1.2721	1.0495	1.012	0.018862005	0.598498639	0.898128103	0.6838209	0.863219191	0.969202988
59215608	Unknown polar (59215608)	0.851	0.7085	0.8219	0.238646956	0.007215022	0.124279327	0.951497416	0.234208529	0.610107695
59215610	Unknown polar (59215610)	1.7458	1.4047	1.0024	0.002864314	0.025791858	0.987297456	0.414348997	0.288796737	0.998080593
59215612	Unknown polar (59215612)	0.9483	1.0141	0.9937	0.673512312	0.903465167	0.956804235	0.963288743	0.980876989	0.995804743
59215613	Unknown polar (59215613)	0.8363	0.9226	0.8701	0.151870196	0.477661507	0.228459898	0.881182388	0.787354133	0.699367036
59215614	Unknown polar (59215614)	1.1867	1.0929	1.0058	0.328665077	0.578661628	0.971433427	0.963288743	0.850192523	0.995804743
59215615	Unknown polar (59215615)	0.9971	0.956	1.4428	0.991289541	0.851704637	0.137166254	0.999882889	0.978653902	0.629175857
59215616	Unknown polar (59215616)	2.1951	1.6766	1.0824	0.033621535	0.123650289	0.814404958	0.6838209	0.467585125	0.963542296
59215618	Unknown polar (59215618)	0.97	0.8965	1.0669	0.748495728	0.210204823	0.462742881	0.967341023	0.568491235	0.849302258
59215619	Unknown polar (59215619)	1.112	1.1061	1.0777	0.369006516	0.351155241	0.494934661	0.963288743	0.693783042	0.857880273
59215620	Unknown polar (59215620)	1.2109	1.3594	1.073	0.541389821	0.28579259	0.813186411	0.963288743	0.651178151	0.963542296
59215621	Unknown polar (59215621)	1.0291	0.9518	0.8513	0.823483664	0.674046377	0.180024021	0.987924779	0.910613009	0.669510821
59215622	Unknown polar (59215622)	1.1793	0.8116	1.003	0.265308086	0.124975069	0.982527346	0.951497416	0.468656508	0.995804743
59215623	Unknown polar (59215623)	0.9984	1.4275	1.0808	0.995281157	0.147584669	0.753384726	0.999882889	0.484186328	0.954126516
59215625	Unknown polar (59215625)	0.639	1.0227	1.4219	0.203635028	0.944079445	0.281005399	0.951323797	0.989177255	0.720313821
59215626	Unknown polar (59215626)	1.0574	0.8256	1.1293	0.630532792	0.074707238	0.26156249	0.963288743	0.400279356	0.713352247
59215627	Unknown polar (59215627)	1.0703	0.8165	0.8547	0.759236334	0.419541741	0.504753643	0.967341023	0.743282613	0.863646917
59215628	Unknown polar (59215628)	1.0964	1.1163	1.039	0.268533715	0.149559777	0.618909131	0.951497416	0.484186328	0.9003228
59215629	Unknown polar (59215629)	1.0796	1.0839	0.916	0.791556412	0.761206572	0.744414242	0.981268279	0.958269877	0.954126516
59215630	Unknown polar (59215630)	0.8095	1.2671	1.0365	0.51934545	0.430652545	0.906032773	0.963288743	0.756114008	0.975394134
59215631	Unknown polar (59215631)	1.0998	1.0644	0.8411	0.366168817	0.516571256	0.07936989	0.963288743	0.79882153	0.517737973
59215632	Unknown polar (59215632)	1.0548	1.537	0.9004	0.903902798	0.27864302	0.797182988	0.987924779	0.649685797	0.963285466
59215633	Unknown polar (59215633)	0.9765	2.9783	0.886	0.95994715	0.026195843	0.763703455	0.995336907	0.288796737	0.954126516
59215635	Unknown polar (59215635)	1.0519	1.0114	0.6786	0.861599883	0.96579569	0.153293927	0.987924779	0.994526454	0.668073386
59215636	Unknown polar (59215636)	0.8442	1.6886	0.7719	0.606775581	0.085290067	0.397836804	0.963288743	0.422989828	0.806425954
59215638	Unknown polar (59215638)	1.0632	1.1099	0.8744	0.643701357	0.390927543	0.277436253	0.963288743	0.730903574	0.720313821
59215639	Unknown polar (59215639)	1.2646	1.5515	0.5965	0.463153655	0.136153749	0.085072728	0.963288743	0.478665525	0.519630104
59215642	Unknown polar (59215642)	0.8622	1.9102	0.6724	0.706841931	0.076408787	0.280366142	0.963288743	0.400279356	0.720313821
59215643	Unknown polar (59215643)	0.9819	0.9996	0.9385	0.874067285	0.997029284	0.552871741	0.987924779	0.998352634	0.882332264
59215644	Unknown polar (59215644)	0.973	0.9474	1.0206	0.805510437	0.594866224	0.843190657	0.987683097	0.862578388	0.96811619
59215645	Unknown polar (59215645)	0.9569	1.0231	0.9523	0.669572125	0.809024135	0.610803863	0.963288743	0.960582746	0.9003228
59215646	Unknown polar (59215646)	1.1246	1.0648	0.8266	0.428794134	0.643266222	0.169117605	0.963288743	0.893425308	0.668073386
39211436	Pantothenic acid	1.163	1.0006	0.9174	0.115599246	0.994524791	0.362980767	0.823138534	0.998352634	0.773702424
19215427	Pyridoxal	1.0149	0.7445	0.7307	0.924187458	0.041568001	0.032961834	0.987924779	0.338224166	0.47122359
19215464	Pyridoxamine	0.7954	0.4993	1.0817	0.482375169	0.022342171	0.794878313	0.963288743	0.288796737	0.963285466
39210083	Threonic acid	1.1391	1.2175	0.8869	0.077109404	0.007267501	0.100578396	0.723327965	0.234208529	0.564118143
19210120	Coenzyme Q10	1.0496	0.7602	1.0261	0.768228795	0.071675363	0.865941234	0.967341023	0.400279356	0.969056278
19210279	Coenzyme Q6	0.8971	0.7233	0.8637	0.689653802	0.195295783	0.562051814	0.963288743	0.559066507	0.882941802
19210281	Coenzyme Q9	1.0279	0.9036	1.0124	0.603109471	0.038883003	0.800972506	0.963288743	0.336487529	0.963285466
19210306	Flavine adenine dinucleotide (FAD)	0.9712	0.9884	1.0145	0.698383776	0.874414872	0.847639509	0.963288743	0.980282352	0.96811619
19210044	Glutathione (GSH)	0.9936	0.8853	1.1947	0.971726842	0.494920478	0.32718073	0.999882889	0.78976672	0.741148315
19210412	Glutathione disulfide (GSSG)	0.9977	0.7884	1.3939	0.993210133	0.361275358	0.210162098	0.999882889	0.694282674	0.693716712
19210328	Nicotinamide	1.0001	0.8833	0.9701	0.999092107	0.02479949	0.581109614	0.999882889	0.288796737	0.889453491
19210372	Nicotineamide adenine dinucleotide (NAD)	1.2987	1.6624	1.1451	0.219095852	0.016441406	0.519669341	0.951497416	0.265637387	0.866490959
19210373	Nicotineamide adenine dinucleotide, reduced (NADH)	1.1782	1.2367	1.0394	0.346418405	0.214077342	0.822659929	0.963288743	0.568491235	0.964054604
29211199	Nicotinic acid	0.994	0.8732	0.9581	0.917389863	0.02006007	0.461079309	0.987924779	0.288796737	0.849302258
19210228	Riboflavin	1.1723	0.8119	0.7638	0.196551292	0.085537943	0.029583049	0.951323797	0.422989828	0.47122359
19215172	Thiamine	0.9999	0.3443	1.1515	0.999882889	0.003716465	0.695568667	0.999882889	0.234208529	0.93434597
29210018	alpha-Tocopherol	0.9687	0.7911	0.745	0.888298944	0.292691911	0.193691623	0.987924779	0.651178151	0.675776419
