# Supplementary material for: Cardiac transcriptional and metabolic changes following thoracotomy
Source: Sci Rep. 2020 Jun 15;10:9673. doi: 10.1038/s41598-020-66721-3 (PMC7295769; doi:10.1038/s41598-020-66721-3)
Supplement: Supplementary file 7 — Supplementary Table 4. [file 41598_2020_66721_MOESM7_ESM.pdf]

Cardiac transcriptional and metabolic changes following thoracotomy

Markus B. Heckmann[1], Ashraf Yusuf Rangrez[2], Daniel Finke[1], Andreas Jungmann[1], Julia S. Kreußer[1], Alexandra Rosskopf[2], Nesrin Schmiedel[2], Hugo A. Katus[1], Norbert Frey[2], Oliver J. Müller[2]*

1 Department of Internal Medicine III, Cardiology, Angiology & Pulmonology, Heidelberg University Hospital, Im Neuenheimer Feld 669, 69120 Heidelberg, Germany, and DZHK (German Center for Cardiovascular Research), partner site
2 Department of Internal Medicine III, University of Kiel, Arnold-Heller-Str. 3, 24105, Kiel, Germany, and DZHK (German Centre for Cardiovascular Research), Partner Site Hamburg/Kiel/Lübeck, Germany

Supplementary Information

Supplementary Table 4: Metabolic Profile Liver

MET_CHEM_NO	Effect_name	ratio 2W	ratio 4W	ratio 6W	p value 2W	p value 4W	p value 6W	FDR 2W	FDR 4W	FDR 6W
39300072	Aspartate	1.16	1.20	0.88	0.0591	0.0662	0.0570	0.8761	0.9690	0.6527
19300164	Glutamate	1.09	1.07	0.86	0.0604	0.0677	0.0583	0.9507	0.9754	0.6474
39300073	Phenylalanine	1.08	1.11	0.93	0.0488	0.0548	0.0471	0.9507	0.9690	0.7549
39300016	Tryptophan	0.99	1.13	0.99	0.0463	0.0519	0.0447	0.9790	0.9690	0.9857
19300173	Tyrosine	1.12	0.96	1.06	0.0618	0.0693	0.0597	0.9411	0.9778	0.8602
19300043	Arginine	0.92	0.92	1.16	0.0663	0.0744	0.0640	0.9507	0.9690	0.6527
39300045	Glutamine	1.14	1.19	0.84	0.1108	0.1242	0.1069	0.9507	0.9690	0.7549
39300466	Histidine	0.94	1.08	1.27	0.0534	0.0599	0.0515	0.9542	0.9690	0.3898
39300013	Lysine	0.78	0.86	1.61	0.0628	0.0704	0.0606	0.7775	0.9690	0.0624
39300074	Isoleucine	1.21	0.94	1.33	0.0613	0.0687	0.0592	0.8083	0.9754	0.3898
39300075	Leucine	1.12	1.02	1.17	0.0491	0.0551	0.0474	0.8893	0.9893	0.5588
39300070	Valine	1.13	0.91	1.34	0.0638	0.0715	0.0615	0.9409	0.9690	0.3898
39300003	Alanine	0.92	1.11	0.99	0.0604	0.0677	0.0582	0.9507	0.9690	0.9857
39300004	Glycine	0.80	1.10	1.31	0.0393	0.0440	0.0379	0.7775	0.9690	0.1026
39300079	Proline	0.97	0.98	1.24	0.0791	0.0887	0.0764	0.9788	0.9979	0.6039
39300052	Serine	1.17	1.42	0.96	0.0743	0.0833	0.0717	0.9144	0.8869	0.9296
39300006	Threonine	1.28	1.11	1.09	0.0673	0.0755	0.0649	0.7775	0.9690	0.8056
39300010	Cysteine (additional: Cystine)	0.87	1.02	1.27	0.0561	0.0630	0.0542	0.8847	0.9933	0.3898
39300008	Methionine	1.11	1.20	1.01	0.0546	0.0612	0.0527	0.9411	0.9581	0.9857
39300546	Taurine	0.77	0.53	1.34	0.0914	0.1025	0.0882	0.8116	0.4388	0.5559
39300559	N2-Acetyllysine	0.89	1.28	1.27	0.0966	0.1083	0.0932	0.9507	0.9690	0.6396
39301049	N-Ethylasparagine	1.07	1.10	1.03	0.0624	0.0700	0.0602	0.9510	0.9690	0.9421
39301135	beta-Alanine (additional: Pantothenic acid)	0.98	0.84	1.25	0.0714	0.0801	0.0689	0.9790	0.9690	0.5651
39300071	Homoserine	0.93	1.20	1.04	0.0439	0.0493	0.0424	0.9507	0.8869	0.8642
39300541	Hypotaurine	0.46	1.14	1.48	0.1431	0.1605	0.1381	0.7775	0.9754	0.6039
39300437	trans-4-Hydroxyproline	0.73	1.14	1.33	0.0661	0.0742	0.0638	0.7775	0.9690	0.3898
19300325	Creatine	0.80	0.89	0.98	0.0587	0.0658	0.0566	0.7775	0.9690	0.9728
19300321	Creatinine	1.24	1.14	1.28	0.1016	0.1140	0.0981	0.9144	0.9690	0.6476
19300362	Phosphocreatine	0.93	0.81	1.20	0.1086	0.1218	0.1048	0.9770	0.9690	0.7469
39301926	S-Adenosylmethionine	1.03	1.60	0.86	0.0775	0.0870	0.0748	0.9770	0.6081	0.7029
39301937	Sarcosine	1.14	0.58	0.93	0.1212	0.1359	0.1169	0.9542	0.8869	0.9296
19300316	O-Phosphotyrosine	0.92	1.23	0.94	0.0653	0.0733	0.0630	0.9507	0.9690	0.8745
19300159	Citrulline	1.05	0.96	1.72	0.0693	0.0778	0.0669	0.9770	0.9861	0.0575
39300046	Ornithine (additional: Arginine, Citrulline)	0.92	1.00	1.47	0.0667	0.0748	0.0644	0.9507	0.9982	0.2064
39300082	Urea	0.89	0.90	1.24	0.0477	0.0535	0.0460	0.8847	0.9690	0.3898
39300021	Maltose	0.90	0.78	0.68	0.2201	0.2468	0.2124	0.9770	0.9732	0.7432
39302122	3-Deoxyglucosone	1.01	1.32	1.38	0.1092	0.1225	0.1054	0.9886	0.9690	0.5866
39300036	Glucose	0.73	0.98	0.89	0.2027	0.2273	0.1956	0.9507	0.9982	0.9296
39300028	Mannose	0.77	0.72	1.10	0.1198	0.1531	0.1114	0.9119	0.9690	0.8841
59300824	Tetroses (additional: Dihydroxybutyrates)	1.04	0.97	1.11	0.0311	0.0349	0.0301	0.9507	0.9754	0.5477
39301909	1,2-Anhydroribose (additional: Nucleosides)	0.96	1.02	0.87	0.0669	0.0751	0.0646	0.9770	0.9979	0.6527
39300019	Ribose	0.92	1.06	0.91	0.0826	0.0927	0.0797	0.9653	0.9778	0.8353
39300014	myo-Inositol	0.93	1.08	1.18	0.0397	0.0445	0.0383	0.9438	0.9690	0.4022
19300140	Sorbitol (additional: Galactitol, Mannitol)	1.02	0.92	1.07	0.0696	0.0780	0.0672	0.9795	0.9690	0.8745
39301823	Glucuronic acid	0.69	1.13	0.92	0.1285	0.1442	0.1240	0.8116	0.9754	0.9296
59300832	Hexose acids	0.96	1.02	1.15	0.0617	0.0692	0.0595	0.9770	0.9979	0.6484
39300596	Saccharic acid	1.04	1.06	0.82	0.0660	0.0667	0.0595	0.9770	0.9754	0.5477
39300044	Maltotriose	0.73	0.92	0.51	0.2451	0.2748	0.2365	0.9507	0.9979	0.6039
29300176	Hentriacontane	1.06	0.86	0.91	0.0368	0.0413	0.0385	0.9507	0.8869	0.6484
59300851	Glycocholic acid	1.26	4.12	1.45	0.1849	0.2414	0.1851	0.9507	0.5102	0.7022
59300809	Taurochenodeoxycholic acid (additional: Taurodeoxycholic acid)	0.97	1.11	1.60	0.1020	0.1144	0.0985	0.9788	0.9754	0.3898
19300396	Taurocholic acid	1.59	0.76	0.88	0.1275	0.1430	0.1231	0.7775	0.9690	0.8642
269300614	Ceramide (d18:1,C24:0)	0.95	1.06	1.10	0.0430	0.0482	0.0415	0.9507	0.9690	0.6527
269300115	Ceramide (d18:1,C24:1) (additional: Ceramide (d18:2,C24:0))	0.93	0.88	0.98	0.0545	0.0611	0.0526	0.9507	0.9690	0.9680
29301295	Cholesta-1,3,5-triene	1.15	0.70	1.12	0.0508	0.0542	0.0541	0.8116	0.4388	0.6586
29301323	Cholesta-2,4-dien	1.00	0.87	0.80	0.0889	0.1055	0.0959	0.9894	0.9690	0.6527
29300522	Cholestenol No 02	1.02	0.91	0.88	0.0559	0.0626	0.0584	0.9788	0.9690	0.6527
19300401	Cholesterol, free	0.95	1.00	0.93	0.0366	0.0411	0.0354	0.9507	0.9982	0.6655
259300019	Cholesterol, total	1.10	0.94	0.84	0.0675	0.0730	0.0628	0.9507	0.9754	0.6120
29300850	Dihydrocholesterol	1.25	1.09	0.80	0.0524	0.0588	0.0548	0.7775	0.9690	0.4389
19300404	Cholesterylester C18:1	0.85	0.83	1.52	0.1146	0.1285	0.1106	0.9507	0.9690	0.5166
19300405	Cholesterylester C18:2	0.96	0.92	1.12	0.1970	0.2209	0.1901	0.9810	0.9924	0.9296
19300406	Cholesterylester C20:4	0.57	0.60	1.58	0.1972	0.2208	0.2057	0.8116	0.9690	0.6527
269300085	Cholesterylester, total	1.08	1.12	0.85	0.0998	0.1119	0.0963	0.9770	0.9732	0.7549
269300114	DAG (C18:1,C18:2)	1.13	1.07	0.92	0.0868	0.0973	0.0838	0.9507	0.9778	0.8745
29300474	14-Methylhexadecanoic acid	0.76	1.22	0.98	0.0898	0.0985	0.0919	0.8116	0.9690	0.9728
29301389	15-Methylhexadecanoic acid	0.94	0.89	1.14	0.0379	0.0425	0.0396	0.9507	0.9690	0.5588
29300478	16-Methylheptadecanoic acid	0.91	0.90	1.24	0.0573	0.0642	0.0598	0.9507	0.9690	0.5375
29300470	Isopalmitic acid (C16:0)	1.10	1.03	1.08	0.0557	0.0625	0.0582	0.9507	0.9832	0.8353
29300070	Eicoasenoic acid (C20:cis[11]1)	0.95	1.13	1.15	0.0857	0.0961	0.0896	0.9770	0.9690	0.7710
29300485	Eicosaenoic acid (C20:1) No 02	0.69	0.97	1.92	0.1019	0.1142	0.1065	0.7775	0.9979	0.1945
259300830	Elaidic acid (C18:trans[9]1)	0.60	0.82	1.53	0.1008	0.1089	0.0937	0.7775	0.9690	0.3898
29300159	Nervonic acid (C24:cis[15]1)	1.00	0.91	0.95	0.0388	0.0435	0.0405	0.9894	0.9690	0.8423
259300006	Oleic acid (C18:cis[9]1)	1.01	1.04	1.00	0.1064	0.1150	0.0990	0.9886	0.9942	0.9999
29300026	Palmitoleic acid (C16:cis[9]1)	0.83	1.01	1.65	0.1043	0.1169	0.1089	0.9411	0.9982	0.3898
259300011	Arachidonic acid (C20:cis[5,8,11,14]4)	1.00	0.92	0.88	0.0299	0.0323	0.0278	0.9886	0.9690	0.3898
29300519	conjugated Linoleic acid (C18:trans[9,11]2) (additional: conjugated Linoleic acid (C18:cis[9]trans[11]2))	1.05	1.26	1.21	0.0850	0.0953	0.0888	0.9770	0.9690	0.6584
259300015	Docosahexaenoic acid (C22:cis[4,7,10,13,16,19]6)	1.25	0.92	0.77	0.0488	0.0527	0.0453	0.7775	0.9690	0.2422
29300490	Docosapentaenoic acid (C22:cis[4,7,10,13,16]5)	0.48	0.81	1.57	0.2440	0.3087	0.2556	0.8116	0.9778	0.7469
259300493	Docosapentaenoic acid (C22:cis[7,10,13,16,19]5)	1.21	1.27	1.04	0.1005	0.1087	0.0935	0.9405	0.9690	0.9528
29300483	Eicosadienoic acid (C20:2) No 02	0.92	1.03	1.14	0.0515	0.0575	0.0566	0.9507	0.9808	0.6484
29300477	gamma-Linolenic acid (C18:cis[6,9,12]3)	1.67	1.08	0.55	0.0740	0.0829	0.0773	0.7775	0.9754	0.0705
259300004	Linoleic acid (C18:cis[9,12]2)	1.42	1.26	0.86	0.1024	0.1184	0.0960	0.7775	0.9690	0.7681
29300005	Linolenic acid (C18:cis[9,12,15]3)	1.49	1.35	0.98	0.1459	0.1635	0.1524	0.8146	0.9690	0.9857
29300013	Ricinoleic acid (12-OH-C18:cis[9]1)	1.09	0.71	0.73	0.1589	0.1781	0.1660	0.9770	0.9690	0.7217
29300152	Behenic acid (C22:0)	1.30	1.24	0.91	0.0563	0.0631	0.0589	0.7775	0.8869	0.7549
29300069	Eicosanoic acid (C20:0)	1.13	1.35	1.03	0.0656	0.0735	0.0685	0.9411	0.8869	0.9421
29300068	Heptadecanoic acid (C17:0)	1.04	1.08	0.78	0.0336	0.0376	0.0351	0.9507	0.9690	0.1026
29300052	Lignoceric acid (C24:0)	1.08	1.19	1.06	0.0373	0.0418	0.0390	0.9069	0.8869	0.8056
29300105	Myristic acid (C14:0)	0.75	0.87	1.37	0.1146	0.1284	0.1197	0.8822	0.9690	0.6245
259300003	Palmitic acid (C16:0)	1.06	1.07	0.97	0.0619	0.0669	0.0575	0.9669	0.9754	0.9296
259300007	Stearic acid (C18:0)	1.02	1.09	0.80	0.0363	0.0392	0.0338	0.9770	0.9690	0.1395
29300072	Tricosanoic acid (C23:0)	1.06	1.01	0.99	0.0325	0.0365	0.0340	0.9405	0.9979	0.9606
29300063	Dodecanol	1.02	1.01	0.88	0.0495	0.0555	0.0518	0.9770	0.9979	0.6474
259300002	Glycerol, lipid fraction	1.07	1.20	0.85	0.1213	0.1311	0.1128	0.9770	0.9690	0.7913
29300056	Hexadecanol	1.02	1.00	0.79	0.0348	0.0389	0.0364	0.9770	0.9983	0.1395
29300073	Pentadecanol	0.78	0.92	1.29	0.0873	0.0979	0.0912	0.8116	0.9754	0.6134
29300109	Tetradecanol	1.08	1.04	0.86	0.0303	0.0340	0.0317	0.8546	0.9690	0.3898
29300139	3-O-Galactoglycerolipids	0.99	0.91	0.87	0.0454	0.0508	0.0485	0.9810	0.9690	0.5973
29300045	Galactose, lipid fraction	1.08	0.96	0.99	0.0372	0.0417	0.0388	0.9346	0.9754	0.9728
29300122	Glucose, lipid fraction	1.15	1.07	0.63	0.1088	0.1220	0.1137	0.9507	0.9778	0.4621
29301369	myo-Inositol, lipid fraction	1.09	1.01	1.21	0.0554	0.0620	0.0578	0.9507	0.9979	0.5588
29300488	N-Acetylneuraminic acid, lipid fraction	1.29	0.92	0.87	0.0925	0.1036	0.0966	0.8146	0.9774	0.8053
69300556	Phosphatidylcholine hydroperoxide (C16:0,C18:2-OOH)	1.20	0.93	0.97	0.0450	0.0504	0.0434	0.7775	0.9690	0.9116
69300427	Phosphatidylcholine hydroperoxide (C18:0,C18:2-OOH)	1.00	1.18	1.07	0.0418	0.0469	0.0403	0.9886	0.8869	0.7469
69300431	Triacylglyceride hydroperoxide (C16:0,C18:1,C18:2-OOH)	1.18	1.00	1.02	0.0400	0.0426	0.0367	0.7775	0.9982	0.9296
39300038	Glycerol, polar fraction	0.88	0.80	0.95	0.1023	0.1147	0.0987	0.9507	0.9690	0.9421
39300053	Glycerol-3-phosphate, polar fraction	0.99	1.30	0.77	0.0608	0.0681	0.0586	0.9886	0.8869	0.3898
39301739	O-Phosphoethanolamine	0.93	1.30	0.93	0.0557	0.0624	0.0537	0.9507	0.8869	0.8353
269300094	Lysophosphatidylcholine (C16:0)	0.97	0.93	0.99	0.0288	0.0323	0.0278	0.9669	0.9690	0.9728
69300508	Lysophosphatidylcholine (C17:0)	0.96	1.00	0.94	0.0380	0.0427	0.0367	0.9507	0.9982	0.7543
269300096	Lysophosphatidylcholine (C18:0)	1.05	1.04	0.80	0.0457	0.0513	0.0441	0.9542	0.9754	0.3897
69300098	Lysophosphatidylcholine (C18:2)	0.75	1.04	1.32	0.0511	0.0573	0.0493	0.7775	0.9778	0.2472
269300100	Lysophosphatidylcholine (C20:4)	0.85	0.56	0.94	0.0768	0.0861	0.0741	0.9119	0.4388	0.8887
269300093	Lysophosphatidylethanolamine (C18:0)	1.22	1.14	0.79	0.0574	0.0643	0.0553	0.7775	0.9690	0.4368
69300505	Lysophosphatidylethanolamine (C18:1)	0.91	0.94	1.10	0.0480	0.0538	0.0463	0.9411	0.9690	0.6655
69300504	Lysophosphatidylethanolamine (C18:2)	1.01	1.19	1.17	0.0543	0.0609	0.0524	0.9810	0.9690	0.5973
19300073	Isopentenyl pyrophosphate (IPP)	0.97	1.22	0.81	0.0696	0.0781	0.0672	0.9770	0.9690	0.5834
19300248	Mevalonate	0.92	0.84	0.93	0.0435	0.0487	0.0419	0.9321	0.8869	0.7575
269300617	Phosphatidylcholine (C16:0,C16:0)	1.00	1.00	1.03	0.0211	0.0237	0.0204	0.9810	0.9979	0.8353
269300129	Phosphatidylcholine (C16:0,C18:2)	0.99	1.03	1.01	0.0112	0.0126	0.0108	0.9770	0.9690	0.9296
269300135	Phosphatidylcholine (C16:0,C20:4) (additional: Phosphatidylcholine (C18:2,C18:2))	1.00	1.05	1.00	0.0111	0.0125	0.0107	0.9886	0.8869	0.9857
269300142	Phosphatidylcholine (C16:0,C22:6) (additional: Phosphatidylcholine (C18:2,C20:4))	0.98	1.02	0.99	0.0105	0.0118	0.0101	0.9507	0.9690	0.8527
269300622	Phosphatidylcholine (C16:1,C18:2)	1.04	1.06	1.13	0.0320	0.0359	0.0309	0.9507	0.9690	0.4727
269300136	Phosphatidylcholine (C18:0,C18:1)	0.95	1.03	1.02	0.0114	0.0128	0.0110	0.7775	0.9690	0.7469
269300628	Phosphatidylcholine (C18:0,C18:2)	0.99	1.03	0.98	0.0107	0.0120	0.0103	0.9770	0.9690	0.6527
69300144	Phosphatidylcholine (C18:0,C20:4)	1.01	1.00	0.98	0.0096	0.0107	0.0092	0.9770	0.9992	0.6527
269300155	Phosphatidylcholine (C18:0,C22:6)	1.04	1.00	0.87	0.0170	0.0191	0.0164	0.8870	0.9979	0.0424
269300143	Phosphatidylcholine No 02	0.97	0.97	1.00	0.0089	0.0099	0.0085	0.7775	0.8869	0.9728
69300164	Di-docosahexaenoyl (C22:6)-bis(monoacylglycerol) phosphate	1.35	1.04	0.70	0.0810	0.0908	0.0782	0.7775	0.9893	0.3898
259300044	Glycerol phosphate, lipid fraction	1.07	0.78	1.24	0.1630	0.1761	0.1516	0.9770	0.9690	0.8045
29300843	myo-Inositol-1-phosphate, lipid fraction (myo-Inositolphospholipids)	0.88	1.24	0.99	0.0531	0.0594	0.0555	0.8870	0.8869	0.9728
29300148	myo-Inositol-2-phosphate, lipid fraction (myo-Inositolphospholipids)	0.90	0.98	1.08	0.0630	0.0707	0.0659	0.9507	0.9942	0.8466
259300086	Phosphate, lipid fraction	1.02	0.96	0.94	0.0354	0.0382	0.0329	0.9770	0.9732	0.7433
29300489	3-O-Methylsphingosine (d18:1) (additional: Sphingolipids, erythro-Sphingosine (d18:1), threo-Sphingosine (d18:1))	1.25	0.92	0.89	0.0494	0.0554	0.0517	0.7775	0.9690	0.6527
29300158	4-Hydroxysphinganine (t18:0, Phytosphingosine), total	1.12	0.91	0.84	0.0340	0.0382	0.0356	0.7775	0.9690	0.3898
29300494	5-O-Methylsphingosine (d18:1) (additional: Sphingolipids, erythro-Sphingosine (d18:1), threo-Sphingosine (d18:1))	1.23	0.90	0.92	0.0386	0.0433	0.0403	0.7775	0.9690	0.7050
29301289	erythro-Dihydrosphingosine (d18:0)	1.09	1.09	0.94	0.0380	0.0426	0.0397	0.8893	0.9690	0.7957
29300495	erythro-Sphingosine (d18:1) (additional: Sphingolipids)	1.04	1.01	1.05	0.0907	0.1017	0.0948	0.9770	0.9979	0.9429
29301391	erythro-Sphingosine-1-phosphate (d18:1)	1.23	0.95	0.89	0.0490	0.0550	0.0512	0.7775	0.9754	0.6527
259300491	threo-Sphingosine (d18:1) (additional: Sphingolipids)	1.06	1.08	0.94	0.0557	0.0602	0.0518	0.9608	0.9690	0.8466
269300122	Sphingomyelin (d18:1,C16:0)	1.00	0.86	0.87	0.0228	0.0255	0.0220	0.9894	0.4388	0.1663
269300126	Sphingomyelin (d18:1,C18:0) (additional: Sphingomyelin (d16:1,C20:0))	0.77	1.01	1.48	0.0460	0.0516	0.0444	0.7775	0.9979	0.0424
269300140	Sphingomyelin (d18:1,C23:0)	1.10	0.99	1.09	0.0347	0.0389	0.0334	0.8116	0.9979	0.6396
69300145	Sphingomyelin (d18:1,C24:0)	1.01	1.01	1.00	0.0122	0.0136	0.0117	0.9770	0.9778	0.9748
269300101	TAG (C16:0,C16:1)	0.84	0.96	0.90	0.2175	0.2439	0.2099	0.9770	0.9979	0.9421
269300162	TAG (C16:0,C18:1,C18:3) (additional: TAG (C16:0,C18:2,C18:2), TAG (C16:1,C18:1,C18:2))	1.59	1.68	0.55	0.1709	0.1917	0.1649	0.8313	0.9690	0.5302

269300602	TAG (C16:0,C18:2)	1.25	1.19	0.68	0.1844	0.2068	0.1779	0.9507	0.9754	0.6561
269300107	TAG (C18:1,C18:2)	1.26	1.46	0.52	0.1752	0.1965	0.1691	0.9507	0.9690	0.4727
269300648	TAG (C18:1,C18:2,C18:3) (additional: TAG (C16:0,C18:1,C20:5), TAG (C16:0,C18:2,C20:4))	1.91	1.47	0.66	0.1601	0.1795	0.1545	0.7775	0.9690	0.6163
269300604	TAG (C18:2,C18:2)	1.96	1.77	0.47	0.2276	0.2552	0.2196	0.8116	0.9690	0.5477
39300012	Citrate (additional: Isocitrate)	0.94	1.29	1.05	0.0568	0.0637	0.0549	0.9553	0.8869	0.8795
39300005	Fumarate	1.07	1.39	0.89	0.0845	0.0948	0.0816	0.9748	0.8869	0.7913
39300007	Malate	1.05	1.41	0.81	0.0927	0.1040	0.0895	0.9770	0.8869	0.6521
59300130	Succinate	1.02	1.21	0.95	0.0493	0.0553	0.0476	0.9770	0.8869	0.8527
39301832	2-Hydroxybutyrate	0.96	0.83	1.22	0.0784	0.0879	0.0757	0.9770	0.9690	0.6245
19300411	Fructose-1,6-diphosphate	0.77	1.88	0.91	0.0998	0.1120	0.0964	0.8469	0.5102	0.8642
39301920	Fructose-6-phosphate	0.80	1.55	1.01	0.1061	0.1190	0.1024	0.9144	0.8869	0.9857
19300212	Glucose-6-phosphate (additional: Fructose-6-phosphate, myo-Inositol-1-phosphate, myo-Inositol-2-phosphate, myo-Inositol-4-phosphate)	0.82	1.40	1.06	0.1054	0.1182	0.1017	0.9411	0.9690	0.9296
39300085	Lactate	0.74	1.12	1.14	0.0833	0.0892	0.0771	0.7775	0.9690	0.7549
39300002	Pyruvate (additional: Phosphoenolpyruvate (PEP))	0.85	1.19	1.09	0.0534	0.0598	0.0515	0.8116	0.9690	0.7549
39300393	3-Hydroxybutyrate	1.30	0.82	0.92	0.0782	0.0877	0.0754	0.7775	0.9690	0.8466
39300930	Ribose-5-phosphate	0.97	1.20	1.13	0.0480	0.0539	0.0464	0.9770	0.8869	0.6245
19300304	Ribulose-5-phosphate	0.94	0.99	1.04	0.0607	0.0680	0.0586	0.9608	0.9982	0.9296
39300995	Sedoheptulose-7-phosphate	0.61	1.15	1.25	0.1085	0.1217	0.1048	0.7775	0.9690	0.6530
39300068	gamma-Aminobutyrate (GABA)	0.72	1.50	0.72	0.1001	0.1204	0.0921	0.7775	0.8869	0.5375
29300021	beta-Sitosterol	0.98	0.91	0.68	0.0648	0.0726	0.0677	0.9790	0.9690	0.2296
29300053	Campesterol	1.17	0.93	0.86	0.0610	0.0684	0.0638	0.8761	0.9690	0.6484
39301553	1,4-Hydroquinone	1.03	1.02	1.11	0.0363	0.0407	0.0350	0.9770	0.9778	0.6039
59300825	Acetylpyruvate (additional: 2,5-Dioxovaleric acid, Itaconic acid)	1.12	1.09	1.13	0.0536	0.0601	0.0517	0.9144	0.9690	0.6484
29300078	Ethanolamine	1.08	1.16	0.98	0.0298	0.0334	0.0311	0.8469	0.8869	0.9259
39300113	Glycerol-2-phosphate	0.96	1.21	0.81	0.0432	0.0485	0.0417	0.9574	0.8869	0.3898
39300051	Phosphate (inorganic and from organic phosphates)	0.90	0.85	1.10	0.0330	0.0370	0.0319	0.7775	0.8869	0.6039
39301737	Pyrophosphate (PPi)	0.74	0.81	1.44	0.0910	0.1021	0.0878	0.7775	0.9690	0.4418
29300852	Serine, lipid fraction	1.06	0.91	0.92	0.0448	0.0502	0.0468	0.9507	0.9690	0.7469
39300011	Putrescine (additional: Agmatine)	0.85	0.67	1.05	0.1096	0.1229	0.1057	0.9507	0.8869	0.9494
39300039	Spermidine	1.01	0.99	0.88	0.0615	0.0690	0.0594	0.9810	0.9979	0.6601
39301750	Adenosine monophosphate (AMP)	0.86	1.02	0.85	0.0546	0.0612	0.0527	0.8116	0.9942	0.5777
39301763	Inosine monophosphate (IMP)	0.95	1.59	0.74	0.1077	0.1208	0.1040	0.9770	0.8869	0.6039
59300846	Uridine monophosphate (UMP)	1.03	1.15	1.02	0.0621	0.0696	0.0599	0.9770	0.9690	0.9728
39301752	Adenine	0.96	1.02	1.08	0.0302	0.0339	0.0292	0.9507	0.9884	0.6245
39300177	Adenosine	0.91	0.48	0.75	0.1342	0.1756	0.1295	0.9770	0.8869	0.6527
39300824	Allantoin	0.89	1.17	1.41	0.0953	0.1069	0.0919	0.9507	0.9690	0.5176
39301925	Guanosine	1.01	0.98	0.88	0.0572	0.0642	0.0552	0.9810	0.9979	0.6527
39300619	Hypoxanthine (additional: Inosine)	1.09	1.17	0.70	0.0979	0.1098	0.0945	0.9669	0.9690	0.4890
39301762	Inosine	1.09	1.37	0.75	0.0732	0.0821	0.0706	0.9507	0.8869	0.4621
39300625	Xanthine	1.29	1.07	0.74	0.1065	0.1195	0.1028	0.8847	0.9778	0.6039
39300441	Cytosine (additional: 2'-Deoxycytidine)	0.98	1.22	1.01	0.0472	0.0529	0.0456	0.9770	0.8869	0.9857
39300534	Uracil	0.92	1.27	0.91	0.0335	0.0375	0.0323	0.8847	0.4388	0.6039
19300407	Uridine	1.19	0.88	0.76	0.0802	0.0900	0.0774	0.9043	0.9690	0.5375
259300676	Unknown lipid (259300676)	0.91	1.03	0.99	0.0535	0.0578	0.0498	0.9507	0.9861	0.9728
269300072	Unknown lipid (269300072)	0.90	0.91	1.04	0.0704	0.0789	0.0679	0.9507	0.9690	0.9421
269300075	Unknown lipid (269300075)	1.03	1.00	0.85	0.0270	0.0303	0.0260	0.9507	0.9982	0.1996
269300103	Unknown lipid (269300103)	1.11	1.63	1.10	0.2434	0.2709	0.2433	0.9770	0.9690	0.9606
269300104	Unknown lipid (269300104)	1.08	1.11	0.93	0.1129	0.1266	0.1090	0.9770	0.9754	0.9259
269300106	Unknown lipid (269300106)	1.19	0.91	1.04	0.0834	0.0935	0.0805	0.9144	0.9732	0.9421
269300108	Unknown lipid (269300108)	1.40	1.24	0.75	0.0898	0.1007	0.0867	0.7775	0.9690	0.5477
269300109	Unknown lipid (269300109)	0.94	1.21	0.74	0.1978	0.2219	0.1909	0.9788	0.9754	0.7766
269300111	Unknown lipid (269300111)	1.27	1.37	0.71	0.2019	0.2281	0.2017	0.9507	0.9690	0.7543
269300112	Unknown lipid (269300112)	0.90	0.93	1.20	0.0476	0.0534	0.0459	0.8870	0.9690	0.4621
269300113	Unknown lipid (269300113)	0.85	2.31	1.09	0.1937	0.2250	0.1936	0.9669	0.8869	0.9569
269300118	Unknown lipid (269300118)	0.86	0.90	1.03	0.0449	0.0504	0.0434	0.7789	0.9690	0.9296
269300127	Unknown lipid (269300127)	0.94	1.00	1.06	0.0193	0.0216	0.0186	0.7775	0.9982	0.5680
269300130	Unknown lipid (269300130)	1.07	1.08	1.04	0.0248	0.0278	0.0239	0.8620	0.9690	0.7913
269300131	Unknown lipid (269300131)	1.15	1.13	0.71	0.0981	0.1101	0.0947	0.9507	0.9690	0.5375
269300137	Unknown lipid (269300137)	1.29	1.14	0.88	0.0396	0.0444	0.0382	0.7775	0.9581	0.5477
269300146	Unknown lipid (269300146)	1.02	0.97	1.02	0.0340	0.0381	0.0328	0.9770	0.9778	0.9421
269300147	Unknown lipid (269300147)	1.16	1.05	1.10	0.0474	0.0531	0.0457	0.7827	0.9743	0.6527
269300148	Unknown lipid (269300148)	1.02	0.96	1.01	0.0415	0.0465	0.0400	0.9770	0.9774	0.9728
269300150	Unknown lipid (269300150)	0.96	0.91	0.86	0.0494	0.0554	0.0476	0.9770	0.9690	0.5826
269300151	Unknown lipid (269300151)	1.07	1.00	1.21	0.0391	0.0438	0.0377	0.9438	0.9982	0.3898
269300152	Unknown lipid (269300152)	1.28	1.51	0.67	0.2122	0.2380	0.2048	0.9507	0.9690	0.7121
269300153	Unknown lipid (269300153)	1.03	1.08	1.34	0.0468	0.0524	0.0451	0.9770	0.9690	0.1525
269300157	Unknown lipid (269300157)	1.07	0.95	0.76	0.0428	0.0480	0.0413	0.9507	0.9732	0.1395
269300159	Unknown lipid (269300159)	0.95	1.30	1.00	0.1209	0.1356	0.1167	0.9770	0.9690	0.9973
269300160	Unknown lipid (269300160)	1.46	0.95	0.86	0.1546	0.1734	0.1492	0.8822	0.9979	0.8601
269300161	Unknown lipid (269300161)	2.18	1.78	0.57	0.2206	0.2474	0.2129	0.7775	0.9690	0.6298
269300163	Unknown lipid (269300163)	0.92	0.86	1.24	0.0553	0.0620	0.0533	0.9507	0.9690	0.4583
269300166	Unknown lipid (269300166)	0.99	1.02	0.94	0.0275	0.0308	0.0265	0.9770	0.9778	0.6527
269300167	Unknown lipid (269300167)	1.65	1.21	0.72	0.1211	0.1358	0.1169	0.7775	0.9690	0.6043
269300168	Unknown lipid (269300168)	1.71	1.47	0.56	0.1546	0.1734	0.1492	0.7775	0.9690	0.4727
269300170	Unknown lipid (269300170)	1.45	0.98	0.67	0.0986	0.1106	0.0952	0.7775	0.9979	0.4368
269300171	Unknown lipid (269300171)	1.67	0.89	0.58	0.1222	0.1371	0.1179	0.7775	0.9754	0.3898
269300517	Unknown lipid (269300517)	1.15	1.46	1.13	0.2194	0.2442	0.2193	0.9770	0.9690	0.9296
269300594	Unknown lipid (269300594)	0.98	1.01	0.82	0.0295	0.0330	0.0284	0.9770	0.9893	0.1026
269300595	Unknown lipid (269300595)	0.84	0.96	1.14	0.0500	0.0560	0.0482	0.7775	0.9754	0.6231
269300596	Unknown lipid (269300596)	1.18	1.12	1.00	0.0819	0.0919	0.0791	0.9144	0.9690	0.9946
269300597	Unknown lipid (269300597)	1.01	0.97	1.03	0.0338	0.0380	0.0327	0.9788	0.9778	0.9082
269300598	Unknown lipid (269300598)	1.02	1.03	0.81	0.0450	0.0504	0.0434	0.9770	0.9778	0.3898
269300600	Unknown lipid (269300600)	1.26	1.04	0.99	0.0512	0.0574	0.0494	0.7775	0.9778	0.9857
269300601	Unknown lipid (269300601)	1.17	1.00	0.67	0.1716	0.1924	0.1656	0.9669	0.9983	0.6484
269300603	Unknown lipid (269300603)	0.89	1.07	0.84	0.1787	0.2004	0.1725	0.9770	0.9942	0.8603
269300605	Unknown lipid (269300605)	0.89	1.18	0.83	0.1810	0.2030	0.1747	0.9770	0.9754	0.8527
269300606	Unknown lipid (269300606)	1.26	1.11	0.94	0.0747	0.0837	0.0721	0.8083	0.9690	0.8979
269300607	Unknown lipid (269300607)	1.63	1.21	0.79	0.1038	0.1164	0.1002	0.7775	0.9690	0.6484
269300608	Unknown lipid (269300608)	1.18	1.26	0.64	0.1880	0.2097	0.1812	0.9669	0.9691	0.6474
269300609	Unknown lipid (269300609)	1.00	0.99	0.64	0.1301	0.1459	0.1255	0.9996	0.9982	0.5375
269300610	Unknown lipid (269300610)	0.93	0.95	0.97	0.0607	0.0681	0.0586	0.9507	0.9776	0.9296
269300611	Unknown lipid (269300611)	1.09	0.97	1.01	0.0569	0.0638	0.0549	0.9507	0.9832	0.9857
269300612	Unknown lipid (269300612)	1.08	1.07	0.99	0.0513	0.0575	0.0495	0.9507	0.9690	0.9857
269300613	Unknown lipid (269300613)	1.58	1.25	0.77	0.1025	0.1149	0.0989	0.7775	0.9690	0.6396
269300615	Unknown lipid (269300615)	0.92	0.83	0.92	0.0335	0.0375	0.0323	0.8761	0.8585	0.6484
269300616	Unknown lipid (269300616)	1.30	1.47	1.25	0.0571	0.0640	0.0551	0.7775	0.4388	0.4621
269300618	Unknown lipid (269300618)	1.03	1.04	0.76	0.0876	0.0983	0.0846	0.9770	0.9893	0.5588
269300619	Unknown lipid (269300619)	0.95	0.99	1.08	0.0228	0.0256	0.0220	0.8847	0.9861	0.5477
269300620	Unknown lipid (269300620)	1.23	1.37	0.98	0.0474	0.0531	0.0457	0.7775	0.4388	0.9646
269300621	Unknown lipid (269300621)	0.84	0.88	1.43	0.0518	0.0580	0.0500	0.7775	0.9690	0.1026
269300623	Unknown lipid (269300623)	0.96	1.00	0.99	0.0096	0.0107	0.0092	0.7775	0.9979	0.7913
269300624	Unknown lipid (269300624)	1.04	1.03	0.96	0.0124	0.0139	0.0120	0.8116	0.9690	0.5375
269300625	Unknown lipid (269300625)	0.99	1.03	1.03	0.0141	0.0158	0.0136	0.9768	0.9690	0.6527
269300626	Unknown lipid (269300626)	1.02	1.01	0.94	0.0107	0.0120	0.0103	0.8962	0.9690	0.1945
269300627	Unknown lipid (269300627)	0.99	1.01	0.99	0.0101	0.0113	0.0097	0.9770	0.9754	0.7856
269300629	Unknown lipid (269300629)	1.15	1.16	1.14	0.0417	0.0468	0.0403	0.7775	0.9176	0.5503
269300630	Unknown lipid (269300630)	0.95	0.92	1.24	0.0394	0.0442	0.0381	0.9507	0.9690	0.2625
269300631	Unknown lipid (269300631)	0.99	1.01	0.98	0.0103	0.0115	0.0099	0.9770	0.9732	0.6527
269300632	Unknown lipid (269300632)	0.98	0.93	0.97	0.0202	0.0226	0.0195	0.9507	0.9581	0.8152
269300633	Unknown lipid (269300633)	1.07	1.07	1.07	0.0238	0.0267	0.0229	0.8116	0.9690	0.5826
269300634	Unknown lipid (269300634)	1.01	0.90	1.06	0.0619	0.0694	0.0597	0.9886	0.9690	0.8745
269300635	Unknown lipid (269300635)	1.91	1.28	0.74	0.2061	0.2312	0.1989	0.7938	0.9692	0.7913
269300636	Unknown lipid (269300636)	1.03	0.99	1.09	0.0359	0.0402	0.0346	0.9684	0.9942	0.6484
269300637	Unknown lipid (269300637)	1.84	1.46	0.70	0.2149	0.2409	0.2073	0.8116	0.9690	0.7469
269300638	Unknown lipid (269300638)	0.96	0.98	1.07	0.0381	0.0427	0.0367	0.9542	0.9893	0.7469
269300639	Unknown lipid (269300639)	1.13	1.19	1.15	0.0433	0.0486	0.0418	0.8116	0.8869	0.5477
269300640	Unknown lipid (269300640)	1.30	1.45	0.69	0.2251	0.2524	0.2172	0.9507	0.9690	0.7549
269300641	Unknown lipid (269300641)	1.10	1.05	0.80	0.0278	0.0311	0.0268	0.7775	0.9690	0.0424
269300642	Unknown lipid (269300642)	0.87	1.02	1.03	0.0373	0.0418	0.0360	0.7775	0.9778	0.8745
269300643	Unknown lipid (269300643)	1.75	1.81	0.48	0.1997	0.2239	0.1927	0.8116	0.9690	0.4949
269300644	Unknown lipid (269300644)	1.34	1.30	0.62	0.1779	0.1995	0.1716	0.9507	0.9690	0.6039
269300645	Unknown lipid (269300645)	1.36	1.04	0.92	0.1118	0.1254	0.1079	0.8251	0.9933	0.9116
269300646	Unknown lipid (269300646)	1.55	1.52	0.78	0.1076	0.1206	0.1038	0.7775	0.8869	0.6484
269300647	Unknown lipid (269300647)	1.69	1.28	0.77	0.1260	0.1413	0.1216	0.7775	0.9690	0.6530
269300649	Unknown lipid (269300649)	1.90	1.39	0.68	0.1696	0.1902	0.1637	0.7775	0.9690	0.6484
269300650	Unknown lipid (269300650)	1.73	1.45	0.57	0.1743	0.1955	0.1683	0.7828	0.9690	0.5477
269300651	Unknown lipid (269300651)	1.29	1.36	0.63	0.1646	0.1846	0.1589	0.9507	0.9690	0.6039
269300652	Unknown lipid (269300652)	1.03	1.09	0.72	0.1858	0.2083	0.1793	0.9886	0.9893	0.7290
269300653	Unknown lipid (269300653)	1.69	1.20	0.69	0.1236	0.1386	0.1192	0.7775	0.9690	0.5687
269300654	Unknown lipid (269300654)	1.63	1.39	0.58	0.1536	0.1722	0.1482	0.7828	0.9690	0.5302
269300655	Unknown lipid (269300655)	1.79	1.50	0.53	0.1660	0.1861	0.1602	0.7775	0.9690	0.4621
269300656	Unknown lipid (269300656)	1.24	1.26	0.65	0.1675	0.1878	0.1616	0.9507	0.9690	0.6245
269300657	Unknown lipid (269300657)	1.28	1.28	0.69	0.1607	0.1802	0.1550	0.9507	0.9690	0.6484
269300658	Unknown lipid (269300658)	1.08	1.09	1.03	0.0381	0.0428	0.0368	0.9144	0.9690	0.9259
269300659	Unknown lipid (269300659)	0.98	0.81	1.19	0.0411	0.0461	0.0397	0.9770	0.8869	0.3946
269300660	Unknown lipid (269300660)	1.40	1.10	0.67	0.1385	0.1553	0.1337	0.8839	0.9778	0.5944
269300661	Unknown lipid (269300661)	1.48	0.78	0.77	0.1141	0.1280	0.1101	0.7775	0.9690	0.6521
29300099	Unknown lipid (29300099)	0.94	1.06	1.02	0.0542	0.0867	0.0675	0.9507	0.9778	0.9728
29300472	Unknown lipid (29300472)	0.91	1.97	1.09	0.1314	0.1719	0.1372	0.9770	0.8869	0.9296
29300473	Unknown lipid (29300473)	0.87	0.85	0.90	0.0457	0.0513	0.0478	0.7828	0.8869	0.6527
29300518	Unknown lipid (29300518)	1.03	1.13	1.12	0.0577	0.0645	0.0603	0.9770	0.9690	0.7125
29300972	Unknown lipid (29300972)	0.98	1.09	1.44	0.1344	0.1504	0.1404	0.9886	0.9778	0.6396
29301279	Unknown lipid (29301279)	0.99	0.90	0.95	0.0427	0.0434	0.0516	0.9788	0.9690	0.8751
29301283	Unknown lipid (29301283)	1.09	0.95	1.00	0.0945	0.1059	0.0987	0.9669	0.9832	0.9999
29301312	Unknown lipid (29301312)	1.17	1.19	0.82	0.0621	0.0697	0.0649	0.8805	0.9690	0.5834
29301355	Unknown lipid (29301355)	0.95	0.93	1.00	0.0480	0.0538	0.0502	0.9608	0.9690	0.9999
29301361	Unknown lipid (29301361)	0.88	1.25	0.99	0.0552	0.0619	0.0577	0.8847	0.8869	0.9857
29301366	Unknown lipid (29301366)	1.03	0.90	0.90	0.0566	0.0634	0.0591	0.9770	0.9690	0.7368
29301374	Unknown lipid (29301374)	0.98	0.95	0.74	0.0404	0.0453	0.0422	0.9770	0.9690	0.1026
29301386	Unknown lipid (29301386)	1.08	0.86	0.66	0.0474	0.0532	0.0496	0.9507	0.9690	0.0424
69300062	Unknown lipid (69300062)	0.97	0.95	0.91	0.0326	0.0366	0.0315	0.9669	0.9690	0.6039
69300063	Unknown lipid (69300063)	0.92	0.90	0.94	0.0271	0.0304	0.0262	0.8116	0.8869	0.6474
69300065	Unknown lipid (69300065)	0.99	1.36	0.72	0.1008	0.1131	0.0973	0.9886	0.9690	0.5477
69300069	Unknown lipid (69300069)	1.03	1.05	0.90	0.0277	0.0311	0.0268	0.9669	0.9690	0.4621
69300070	Unknown lipid (69300070)	0.99	1.14	0.88	0.0283	0.0318	0.0274	0.9788	0.8869	0.3898
69300071	Unknown lipid (69300071)	0.93	1.53	1.04	0.1246	0.1398	0.1203	0.9770	0.9581	0.9660
69300074	Unknown lipid (69300074)	0.97	1.08	0.95	0.0775	0.0869	0.0748	0.9770	0.9754	0.9296
69300076	Unknown lipid (69300076)	0.99	0.89	0.86	0.0350	0.0393	0.0338	0.9770	0.9690	0.3898
69300077	Unknown lipid (69300077)	0.91	1.05	1.04	0.0352	0.0395	0.0340	0.8116	0.9690	0.8353

69300078	Unknown lipid (69300078)	0.90	0.97	0.78	0.0906	0.1016	0.0875	0.9507	0.9979	0.6039
69300080	Unknown lipid (69300080)	0.91	0.99	0.92	0.1014	0.1137	0.0978	0.9608	0.9982	0.9076
69300081	Unknown lipid (69300081)	0.87	0.98	1.19	0.0505	0.0567	0.0488	0.8116	0.9893	0.5375
69300083	Unknown lipid (69300083)	1.05	0.92	0.97	0.0292	0.0327	0.0282	0.9507	0.9690	0.8353
69300086	Unknown lipid (69300086)	0.89	0.97	1.12	0.0339	0.0381	0.0327	0.7775	0.9754	0.5375
69300087	Unknown lipid (69300087)	1.13	1.03	0.98	0.0572	0.0642	0.0552	0.9069	0.9832	0.9606
69300088	Unknown lipid (69300088)	1.09	0.86	0.96	0.0673	0.0755	0.0650	0.9507	0.9690	0.9296
69300102	Unknown lipid (69300102)	1.35	1.35	0.72	0.1219	0.1367	0.1176	0.8805	0.9690	0.6039
69300105	Unknown lipid (69300105)	0.89	0.96	0.99	0.0591	0.0662	0.0570	0.9234	0.9778	0.9857
69300110	Unknown lipid (69300110)	0.89	1.05	1.05	0.0929	0.1042	0.0897	0.9507	0.9893	0.9421
69300116	Unknown lipid (69300116)	1.13	1.31	1.16	0.0742	0.0833	0.0716	0.9507	0.8869	0.6662
69300121	Unknown lipid (69300121)	1.04	0.81	0.85	0.0413	0.0463	0.0399	0.9608	0.8869	0.4368
69300124	Unknown lipid (69300124)	0.99	1.24	1.47	0.0404	0.0453	0.0390	0.9810	0.8869	0.0403
69300125	Unknown lipid (69300125)	1.06	0.97	1.04	0.0356	0.0399	0.0344	0.9507	0.9754	0.8527
69300128	Unknown lipid (69300128)	1.13	0.88	1.05	0.0380	0.0426	0.0366	0.7789	0.9581	0.7913
69300132	Unknown lipid (69300132)	1.17	0.95	0.94	0.0377	0.0423	0.0364	0.7775	0.9690	0.7469
69300138	Unknown lipid (69300138)	1.08	0.87	0.96	0.0375	0.0420	0.0362	0.9359	0.8869	0.8466
69300139	Unknown lipid (69300139)	1.04	0.79	0.89	0.0350	0.0392	0.0338	0.9542	0.4388	0.5477
69300141	Unknown lipid (69300141)	0.99	1.03	0.97	0.0100	0.0112	0.0097	0.9507	0.9690	0.5375
69300149	Unknown lipid (69300149)	1.21	0.82	0.84	0.0363	0.0407	0.0350	0.7775	0.8585	0.3897
69300154	Unknown lipid (69300154)	0.89	0.91	1.06	0.0271	0.0304	0.0262	0.7775	0.8869	0.6530
69300158	Unknown lipid (69300158)	1.04	0.74	0.94	0.0364	0.0409	0.0352	0.9574	0.4388	0.7549
69300165	Unknown lipid (69300165)	1.13	0.88	0.74	0.0528	0.0592	0.0509	0.8847	0.9690	0.2296
69300172	Unknown lipid (69300172)	1.01	0.99	0.96	0.0359	0.0403	0.0347	0.9822	0.9979	0.8234
69300173	Unknown lipid (69300173)	1.01	1.01	0.94	0.0382	0.0428	0.0368	0.9790	0.9942	0.7630
69300481	Unknown lipid (69300481)	0.93	1.00	0.97	0.1417	0.1589	0.1367	0.9770	0.9982	0.9728
69300483	Unknown lipid (69300483)	0.81	1.47	0.87	0.1636	0.1834	0.1579	0.9507	0.9690	0.8788
69300484	Unknown lipid (69300484)	1.02	1.24	0.95	0.0923	0.1035	0.0891	0.9875	0.9690	0.9296
69300485	Unknown lipid (69300485)	1.18	0.92	0.73	0.0699	0.0784	0.0675	0.8870	0.9690	0.3898
69300486	Unknown lipid (69300486)	1.08	0.73	0.75	0.0578	0.0648	0.0557	0.9507	0.8585	0.3536
69300487	Unknown lipid (69300487)	0.88	0.92	1.02	0.0314	0.0352	0.0303	0.7775	0.9690	0.9296
69300490	Unknown lipid (69300490)	1.08	0.96	1.31	0.0608	0.0682	0.0587	0.9507	0.9778	0.3898
69300491	Unknown lipid (69300491)	0.89	0.82	0.90	0.0352	0.0395	0.0340	0.7807	0.7264	0.5973
69300493	Unknown lipid (69300493)	1.46	1.27	0.75	0.0771	0.0865	0.0744	0.7775	0.9690	0.4727
69300494	Unknown lipid (69300494)	1.05	1.06	1.10	0.0358	0.0402	0.0346	0.9507	0.9690	0.6245
69300495	Unknown lipid (69300495)	1.29	1.07	0.79	0.0598	0.0670	0.0577	0.7775	0.9692	0.4583
69300496	Unknown lipid (69300496)	0.85	0.75	1.19	0.0503	0.0564	0.0485	0.7775	0.7185	0.5375
69300497	Unknown lipid (69300497)	1.49	0.78	0.61	0.1536	0.1723	0.1482	0.8546	0.9690	0.5477
69300498	Unknown lipid (69300498)	1.28	0.84	0.73	0.1520	0.1705	0.1467	0.9507	0.9732	0.6561
69300499	Unknown lipid (69300499)	1.50	0.92	0.87	0.1221	0.1370	0.1178	0.7775	0.9778	0.8397
69300500	Unknown lipid (69300500)	0.93	0.85	0.90	0.0386	0.0433	0.0372	0.9411	0.8869	0.6039
69300501	Unknown lipid (69300501)	0.99	1.15	0.95	0.0385	0.0432	0.0372	0.9818	0.8869	0.7957
69300502	Unknown lipid (69300502)	1.14	0.80	0.78	0.0859	0.0963	0.0829	0.9507	0.9690	0.5973
69300503	Unknown lipid (69300503)	1.06	1.11	0.98	0.0398	0.0446	0.0384	0.9507	0.9690	0.9296
69300506	Unknown lipid (69300506)	0.99	1.04	0.97	0.0133	0.0149	0.0128	0.9770	0.9690	0.6413
69300507	Unknown lipid (69300507)	0.81	0.89	1.06	0.0548	0.0615	0.0529	0.7775	0.9690	0.8470
69300509	Unknown lipid (69300509)	0.98	1.17	0.71	0.0767	0.0860	0.0740	0.9788	0.9690	0.3898
69300510	Unknown lipid (69300510)	0.95	1.06	1.08	0.0307	0.0345	0.0297	0.9507	0.9690	0.6163
69300511	Unknown lipid (69300511)	1.17	0.99	0.86	0.0767	0.0861	0.0741	0.9144	0.9982	0.6890
69300512	Unknown lipid (69300512)	0.99	1.00	1.02	0.0760	0.0852	0.0733	0.9886	0.9982	0.9748
69300513	Unknown lipid (69300513)	0.93	0.99	0.98	0.0431	0.0484	0.0416	0.9507	0.9982	0.9609
69300515	Unknown lipid (69300515)	0.78	0.78	0.94	0.0576	0.0646	0.0556	0.7775	0.8869	0.8603
69300516	Unknown lipid (69300516)	0.73	0.75	0.91	0.0627	0.0703	0.0605	0.7775	0.8869	0.7856
69300519	Unknown lipid (69300519)	0.93	0.84	0.90	0.0530	0.0595	0.0512	0.9507	0.9690	0.6530
69300520	Unknown lipid (69300520)	0.76	0.96	1.26	0.0979	0.1098	0.0945	0.8116	0.9893	0.6484
69300521	Unknown lipid (69300521)	0.98	0.92	0.97	0.0739	0.0828	0.0713	0.9790	0.9754	0.9646
69300522	Unknown lipid (69300522)	1.20	1.09	0.82	0.0554	0.0621	0.0535	0.7775	0.9690	0.5375
69300523	Unknown lipid (69300523)	1.02	0.98	1.00	0.0870	0.0975	0.0839	0.9810	0.9979	0.9947
69300524	Unknown lipid (69300524)	1.10	1.19	0.92	0.0483	0.0541	0.0466	0.9144	0.8869	0.7469
69300525	Unknown lipid (69300525)	1.01	1.17	0.81	0.1116	0.1252	0.1077	0.9886	0.9690	0.7059
69300526	Unknown lipid (69300526)	1.02	1.05	0.97	0.0533	0.0598	0.0515	0.9770	0.9778	0.9296
69300527	Unknown lipid (69300527)	1.04	1.11	0.97	0.0286	0.0321	0.0276	0.9507	0.9188	0.8551
69300528	Unknown lipid (69300528)	0.94	1.10	1.20	0.0639	0.0716	0.0617	0.9653	0.9690	0.5973
69300529	Unknown lipid (69300529)	1.06	1.12	0.93	0.0276	0.0310	0.0267	0.9061	0.8869	0.6453
69300530	Unknown lipid (69300530)	1.18	1.20	0.91	0.0426	0.0477	0.0411	0.7775	0.8869	0.6521
69300531	Unknown lipid (69300531)	0.98	1.00	0.95	0.0179	0.0200	0.0172	0.9608	0.9979	0.5588
69300532	Unknown lipid (69300532)	0.97	0.98	0.89	0.1029	0.1154	0.0993	0.9790	0.9979	0.8470
69300533	Unknown lipid (69300533)	0.95	0.96	1.13	0.0316	0.0355	0.0305	0.9507	0.9690	0.4875
69300534	Unknown lipid (69300534)	0.86	0.93	1.14	0.0432	0.0484	0.0417	0.7775	0.9690	0.5826
69300535	Unknown lipid (69300535)	1.60	1.22	0.50	0.1479	0.1659	0.1428	0.7827	0.9690	0.3898
69300536	Unknown lipid (69300536)	1.09	0.98	0.67	0.1084	0.1216	0.1046	0.9768	0.9979	0.4875
69300537	Unknown lipid (69300537)	0.95	1.12	1.04	0.0342	0.0383	0.0330	0.9507	0.9690	0.8353
69300538	Unknown lipid (69300538)	0.92	0.93	1.04	0.0264	0.0296	0.0254	0.7789	0.9690	0.7681
69300539	Unknown lipid (69300539)	1.02	1.02	0.90	0.0232	0.0260	0.0224	0.9608	0.9778	0.3898
69300540	Unknown lipid (69300540)	0.93	1.19	1.18	0.0385	0.0431	0.0371	0.9405	0.8869	0.3946
69300541	Unknown lipid (69300541)	1.16	0.97	0.84	0.0365	0.0409	0.0352	0.7775	0.9754	0.3898
69300542	Unknown lipid (69300542)	1.06	0.90	0.90	0.0624	0.0700	0.0602	0.9669	0.9690	0.7543
69300543	Unknown lipid (69300543)	0.92	0.96	0.97	0.0482	0.0541	0.0465	0.9507	0.9778	0.9152
69300544	Unknown lipid (69300544)	1.08	0.98	0.91	0.0319	0.0357	0.0307	0.8761	0.9836	0.5642
69300545	Unknown lipid (69300545)	0.75	0.93	1.27	0.0533	0.0598	0.0514	0.7775	0.9690	0.3898
69300546	Unknown lipid (69300546)	0.99	0.83	0.90	0.0497	0.0557	0.0479	0.9886	0.8869	0.6527
69300547	Unknown lipid (69300547)	0.89	1.00	1.38	0.0504	0.0566	0.0487	0.8870	0.9982	0.1395
69300549	Unknown lipid (69300549)	0.96	0.93	1.12	0.0419	0.0469	0.0404	0.9542	0.9690	0.6039
69300550	Unknown lipid (69300550)	0.95	1.06	1.24	0.0398	0.0446	0.0384	0.9507	0.9690	0.2547
69300551	Unknown lipid (69300551)	1.22	1.14	0.92	0.0475	0.0533	0.0459	0.7775	0.9690	0.7433
69300552	Unknown lipid (69300552)	1.07	1.03	0.91	0.0304	0.0341	0.0293	0.9144	0.9754	0.5765
69300553	Unknown lipid (69300553)	0.85	0.90	1.44	0.0437	0.0490	0.0421	0.7775	0.9690	0.0424
69300554	Unknown lipid (69300554)	1.05	1.03	0.90	0.0193	0.0216	0.0186	0.8847	0.9690	0.2472
69300555	Unknown lipid (69300555)	1.13	1.11	0.93	0.0692	0.0776	0.0667	0.9411	0.9690	0.8426
69300557	Unknown lipid (69300557)	1.16	0.94	1.03	0.0425	0.0476	0.0410	0.7775	0.9690	0.9296
69300558	Unknown lipid (69300558)	0.93	0.99	1.21	0.0243	0.0272	0.0234	0.8116	0.9924	0.0500
69300559	Unknown lipid (69300559)	0.97	0.89	1.01	0.0263	0.0295	0.0253	0.9577	0.8869	0.9606
69300560	Unknown lipid (69300560)	1.05	1.09	0.88	0.0317	0.0355	0.0306	0.9507	0.9690	0.4055
69300561	Unknown lipid (69300561)	1.34	1.11	0.92	0.0708	0.0794	0.0683	0.7775	0.9690	0.8351
69300562	Unknown lipid (69300562)	1.10	0.96	0.77	0.0364	0.0408	0.0351	0.8457	0.9754	0.0798
69300563	Unknown lipid (69300563)	1.00	1.05	1.12	0.0413	0.0463	0.0399	0.9886	0.9690	0.6039
69300564	Unknown lipid (69300564)	1.01	0.79	0.92	0.0307	0.0344	0.0296	0.9770	0.4388	0.6039
69300565	Unknown lipid (69300565)	1.85	1.98	0.61	0.2139	0.2397	0.2064	0.8116	0.9690	0.6484
69300566	Unknown lipid (69300566)	1.04	1.19	1.11	0.0434	0.0487	0.0419	0.9761	0.8869	0.6484
69300567	Unknown lipid (69300567)	1.04	0.77	0.84	0.0391	0.0438	0.0377	0.9542	0.4388	0.3898
69300568	Unknown lipid (69300568)	1.03	0.98	0.86	0.0393	0.0440	0.0379	0.9770	0.9884	0.4583
69300569	Unknown lipid (69300569)	1.04	0.75	0.88	0.0424	0.0475	0.0409	0.9669	0.4388	0.5651
69300570	Unknown lipid (69300570)	1.04	0.87	0.91	0.0287	0.0322	0.0277	0.9507	0.8869	0.5477
69300571	Unknown lipid (69300571)	1.92	1.84	0.64	0.2129	0.2388	0.2055	0.8083	0.9690	0.6527
69300572	Unknown lipid (69300572)	1.14	0.85	0.85	0.0478	0.0536	0.0461	0.8116	0.9235	0.5375
69300573	Unknown lipid (69300573)	0.84	1.08	1.06	0.0509	0.0571	0.0492	0.7775	0.9690	0.8423
69300574	Unknown lipid (69300574)	1.13	1.11	1.01	0.0269	0.0301	0.0259	0.7775	0.8869	0.9728
69300575	Unknown lipid (69300575)	1.02	0.78	0.92	0.0336	0.0377	0.0325	0.9770	0.4388	0.6396
69300576	Unknown lipid (69300576)	1.11	0.86	0.80	0.0426	0.0478	0.0411	0.8847	0.8869	0.3002
69300577	Unknown lipid (69300577)	1.07	0.80	0.65	0.0568	0.0638	0.0549	0.9507	0.8869	0.0624
69300578	Unknown lipid (69300578)	1.01	0.85	0.76	0.0477	0.0535	0.0461	0.9790	0.9188	0.2191
69300579	Unknown lipid (69300579)	1.16	0.99	0.97	0.1254	0.1406	0.1210	0.9507	0.9982	0.9728
69300581	Unknown lipid (69300581)	1.47	1.09	0.72	0.1228	0.1377	0.1185	0.7982	0.9778	0.6039
69300582	Unknown lipid (69300582)	1.28	1.08	0.85	0.0731	0.0819	0.0705	0.7775	0.9754	0.6521
69300583	Unknown lipid (69300583)	0.81	0.85	0.98	0.0359	0.0403	0.0347	0.7775	0.8869	0.9494
69300584	Unknown lipid (69300584)	1.01	1.00	0.87	0.0319	0.0358	0.0308	0.9790	0.9979	0.3898
69300585	Unknown lipid (69300585)	0.97	1.03	1.01	0.0449	0.0504	0.0433	0.9770	0.9778	0.9728
69300586	Unknown lipid (69300586)	1.60	1.08	0.77	0.1198	0.1344	0.1156	0.7775	0.9778	0.6527
69300587	Unknown lipid (69300587)	1.48	1.07	0.84	0.0807	0.0905	0.0779	0.7775	0.9774	0.6527
69300588	Unknown lipid (69300588)	1.25	1.04	0.74	0.0871	0.0977	0.0841	0.8761	0.9922	0.5375
69300589	Unknown lipid (69300589)	2.50	1.38	0.47	0.1713	0.1921	0.1653	0.7775	0.9690	0.3898
69300590	Unknown lipid (69300590)	1.19	0.95	0.79	0.0935	0.1049	0.0902	0.9411	0.9861	0.6245
69300591	Unknown lipid (69300591)	2.03	1.16	0.63	0.1336	0.1499	0.1290	0.7775	0.9754	0.5375
69300592	Unknown lipid (69300592)	1.81	1.03	0.52	0.1371	0.1537	0.1323	0.7775	0.9979	0.3898
69300593	Unknown lipid (69300593)	1.19	1.43	1.13	0.0856	0.0960	0.0826	0.9315	0.8869	0.7935
39300165	Unknown polar (39300165)	0.91	1.64	1.27	0.1358	0.1490	0.1356	0.9770	0.8869	0.7469
39300348	Unknown polar (39300348)	1.64	0.76	1.02	0.1474	0.1653	0.1422	0.7775	0.9690	0.9857
39300430	Unknown polar (39300430)	0.94	1.04	0.91	0.0360	0.0404	0.0347	0.9507	0.9754	0.6039
39300471	Unknown polar (39300471)	1.26	1.56	1.50	0.1690	0.1895	0.1631	0.9507	0.9690	0.6474
39300583	Unknown polar (39300583)	0.69	0.97	0.62	0.1967	0.2206	0.1898	0.9409	0.9979	0.6474
39300992	Unknown polar (39300992)	0.64	1.18	1.01	0.0734	0.0823	0.0708	0.7775	0.9690	0.9844
39301137	Unknown polar (39301137)	1.17	0.72	0.86	0.1550	0.1696	0.1460	0.9608	0.9690	0.8642
39301139	Unknown polar (39301139)	0.88	0.73	1.13	0.0579	0.0649	0.0559	0.8893	0.8727	0.6527
39301147	Unknown polar (39301147)	0.86	1.17	1.08	0.0708	0.0795	0.0684	0.9043	0.9690	0.8527
39301150	Unknown polar (39301150)	0.92	1.44	1.03	0.0893	0.1002	0.0862	0.9669	0.8869	0.9675
39301255	Unknown polar (39301255)	0.96	1.05	0.97	0.0202	0.0226	0.0195	0.9411	0.9690	0.8053
39301275	Unknown polar (39301275)	1.06	1.43	0.99	0.0919	0.1030	0.0887	0.9770	0.8869	0.9857
39301564	Unknown polar (39301564)	1.38	2.01	0.89	0.0798	0.0895	0.0770	0.7775	0.4388	0.7795
39301907	Unknown polar (39301907)	0.82	1.22	0.81	0.0599	0.0672	0.0578	0.7775	0.9690	0.5202
39302075	Unknown polar (39302075)	0.79	1.33	1.12	0.0856	0.0959	0.0842	0.8116	0.9690	0.8152
39302124	Unknown polar (39302124)	0.78	1.97	0.75	0.0840	0.0942	0.0811	0.8116	0.4388	0.5375
39302219	Unknown polar (39302219)	0.84	1.03	1.08	0.0597	0.0669	0.0576	0.8116	0.9893	0.8018
39302325	Unknown polar (39302325)	1.16	1.34	1.13	0.0776	0.0870	0.0748	0.9359	0.8869	0.7681
59300137	Unknown polar (59300137)	1.09	1.22	1.08	0.0669	0.0750	0.0646	0.9507	0.9690	0.8353
59300141	Unknown polar (59300141)	0.98	1.14	0.81	0.0658	0.0738	0.0635	0.9790	0.9690	0.5588
59300162	Unknown polar (59300162)	1.00	1.15	0.93	0.0578	0.0648	0.0558	0.9996	0.9690	0.8353
59300805	Unknown polar (59300805)	1.10	0.95	1.27	0.0996	0.1117	0.0961	0.9669	0.9893	0.6484
59300810	Unknown polar (59300810)	1.57	0.90	1.13	0.1244	0.1395	0.1200	0.7775	0.9766	0.8642
59300812	Unknown polar (59300812)	1.56	0.76	0.87	0.1189	0.1333	0.1147	0.7775	0.9690	0.8423
59300822	Unknown polar (59300822)	0.97	1.05	1.23	0.0838	0.0939	0.0808	0.9770	0.9778	0.6396
59300828	Unknown polar (59300828)	0.94	0.93	0.84	0.0938	0.1052	0.0906	0.9770	0.9778	0.7125
59300829	Unknown polar (59300829)	1.05	1.16	0.82	0.0797	0.0894	0.0769	0.9770	0.9690	0.6306
59300830	Unknown polar (59300830)	1.05	1.14	0.93	0.0489	0.0548	0.0472	0.9574	0.9690	0.7913
59300831	Unknown polar (59300831)	0.90	1.13	1.01	0.0802	0.0899	0.0774	0.9507	0.9690	0.9855
59300833	Unknown polar (59300833)	1.08	0.89	0.71	0.0761	0.0853	0.0734	0.9574	0.9690	0.3898
59300834	Unknown polar (59300834)	1.07	0.89	0.95	0.0836	0.0938	0.0807	0.9770	0.9690	0.9296
59300835	Unknown polar (59300835)	1.30	1.23	0.84	0.0554	0.0621	0.0535	0.7775	0.8869	0.5651
59300837	Unknown polar (59300837)	0.84	0.83	1.22	0.0466	0.0523	0.0450	0.7775	0.8869	0.3946
59300838	Unknown polar (59300838)	1.02	1.16	0.63	0.0765	0.0858	0.0738	0.9810	0.9690	0.1825

59300839	Unknown polar (59300839)	0.72	0.73	1.30	0.0802	0.0900	0.0774	0.7775	0.8869	0.5477
59300840	Unknown polar (59300840)	1.15	0.92	0.79	0.1109	0.1244	0.1070	0.9507	0.9778	0.6530
59300841	Unknown polar (59300841)	1.20	1.04	1.00	0.0803	0.0901	0.0775	0.8893	0.9893	0.9947
59300842	Unknown polar (59300842)	1.00	1.03	1.06	0.0650	0.0729	0.0628	0.9894	0.9893	0.8887
59300844	Unknown polar (59300844)	0.70	0.86	1.24	0.1011	0.1134	0.0976	0.7775	0.9690	0.6527
59300845	Unknown polar (59300845)	1.01	1.19	0.97	0.1077	0.1208	0.1040	0.9886	0.9690	0.9728
59300847	Unknown polar (59300847)	0.93	1.09	1.22	0.1096	0.1229	0.1057	0.9770	0.9778	0.7238
59300848	Unknown polar (59300848)	1.01	0.82	1.35	0.0762	0.0855	0.0736	0.9886	0.9690	0.4621
59300850	Unknown polar (59300850)	1.13	0.85	0.78	0.0714	0.0800	0.0689	0.9507	0.9690	0.5375
59300857	Unknown polar (59300857)	0.74	1.18	0.96	0.1245	0.1397	0.1202	0.8847	0.9690	0.9728
59300859	Unknown polar (59300859)	1.13	0.82	1.00	0.0790	0.0886	0.0762	0.9507	0.9690	0.9999
59300860	Unknown polar (59300860)	1.16	1.44	0.59	0.0873	0.0979	0.0843	0.9507	0.8869	0.1769
59300861	Unknown polar (59300861)	1.18	1.95	0.78	0.1060	0.1189	0.1023	0.9507	0.5102	0.6484
59300862	Unknown polar (59300862)	0.84	0.99	0.75	0.0863	0.0968	0.0833	0.9144	0.9982	0.5375
59300863	Unknown polar (59300863)	0.66	0.85	1.58	0.1011	0.1134	0.0976	0.7775	0.9690	0.3898
59300865	Unknown polar (59300865)	1.37	1.88	0.51	0.1633	0.1831	0.1576	0.9377	0.8869	0.4234
19300225	Pantothenic acid	1.24	0.82	1.06	0.1567	0.1758	0.1513	0.9507	0.9690	0.9606
39300159	Ascorbic acid (additional: Glucose)	0.81	0.96	0.94	0.0838	0.0939	0.0808	0.8805	0.9893	0.9082
39300083	Threonic acid	0.92	1.06	0.96	0.0363	0.0407	0.0350	0.8847	0.9690	0.8351
19300051	beta-Carotene	1.56	0.82	0.89	0.1208	0.1355	0.1166	0.7775	0.9690	0.8704
19300120	Coenzyme Q10	1.28	1.06	0.76	0.0641	0.0719	0.0619	0.7775	0.9754	0.3898
19300279	Coenzyme Q6	1.17	1.05	0.86	0.0692	0.0776	0.0668	0.9043	0.9778	0.6527
19300280	Coenzyme Q7	0.98	1.08	1.10	0.0601	0.0674	0.0580	0.9790	0.9692	0.7719
19300281	Coenzyme Q9	0.98	1.04	1.05	0.0436	0.0489	0.0421	0.9770	0.9754	0.8366
19300306	Flavin adenine dinucleotide (FAD)	0.95	0.96	1.02	0.0224	0.0251	0.0216	0.9069	0.9690	0.9127
19300044	Glutathione (GSH)	0.57	0.94	1.11	0.0871	0.0976	0.0840	0.7775	0.9778	0.8353
19300412	Glutathione disulfide (GSSG)	0.84	1.11	1.12	0.0442	0.0496	0.0427	0.7775	0.9690	0.6163
39301735	Nicotinamide	0.96	1.02	1.07	0.0225	0.0252	0.0217	0.9321	0.9774	0.5572
19300372	Nicotinamide adenine dinucleotide (NAD)	1.01	1.37	1.24	0.0759	0.0851	0.0733	0.9886	0.8869	0.6039
19300474	Nicotinamide adenine dinucleotide phosphate, reduced (NADPH)	1.36	1.31	1.01	0.0995	0.1115	0.0960	0.7994	0.9690	0.9857
19300373	Nicotinamide adenine dinucleotide, reduced (NADH)	0.93	1.11	1.10	0.0886	0.0993	0.0855	0.9768	0.9692	0.8551
29301199	Nicotinic acid	0.93	0.88	0.95	0.0602	0.0675	0.0629	0.9538	0.9690	0.9124
19300228	Riboflavin	1.04	1.14	0.86	0.0496	0.0556	0.0479	0.9768	0.9690	0.5765
19300217	alpha-Tocopherol	1.03	0.95	0.94	0.0388	0.0435	0.0375	0.9669	0.9690	0.7560
